# Supplementary material for: Aging and metabolism contribute separately to brain–body health
Source: PLoS Biol. 2026 Jun 15;24(6):e3003856. doi: 10.1371/journal.pbio.3003856 (PMC13293518; doi:10.1371/journal.pbio.3003856)
Supplement: S1 Fig — Relationship between participants’ age (x-axis) and raw biomarker values (y-axis) (blue: male, red: female). (PDF) [file pbio.3003856.s001.pdf]

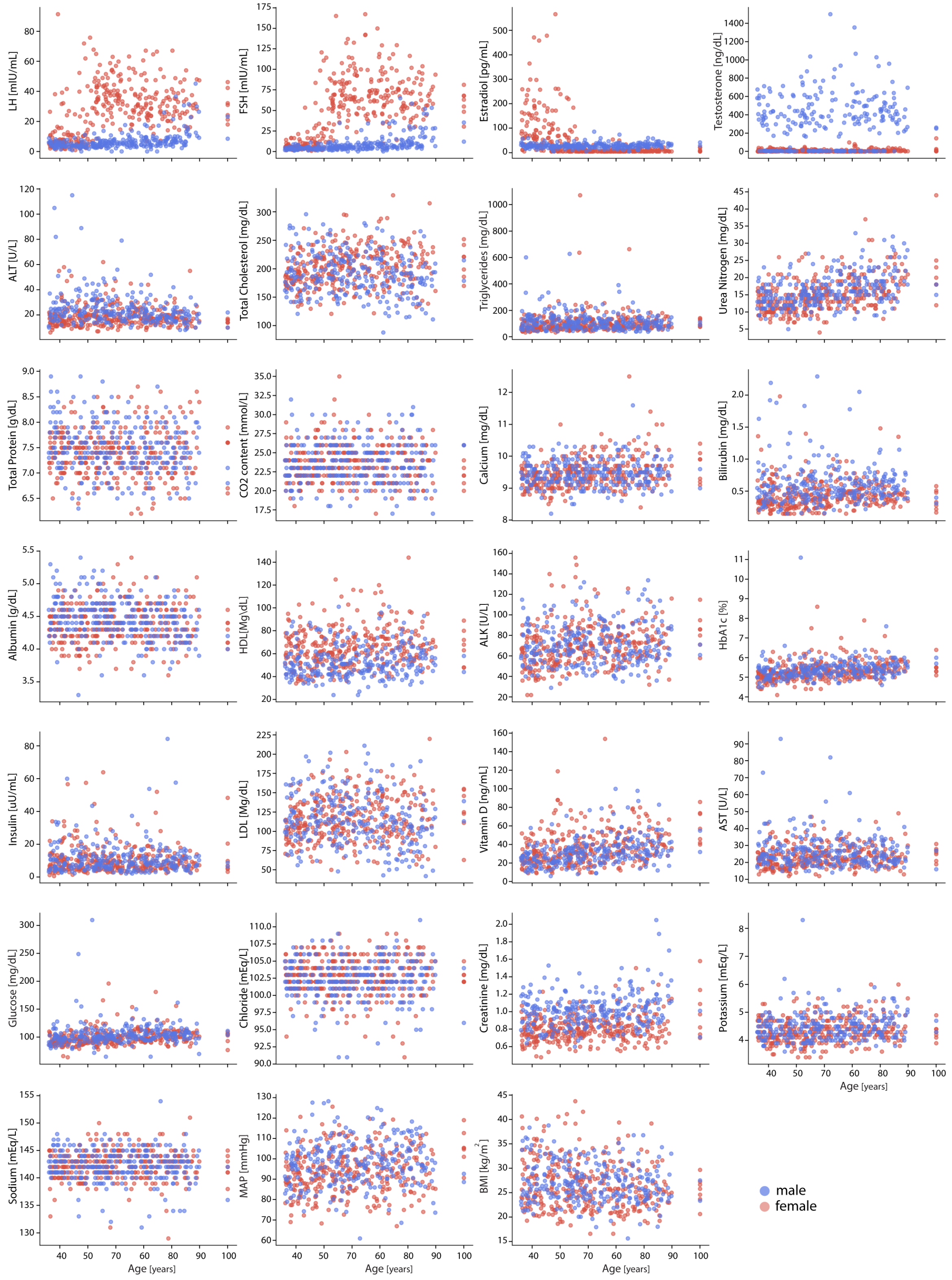

Figure S1. **HCP–A biomarkers versus age.** Relationship between participants' age (x-axis) and raw biomarker values (y-axis) (blue: male, red: female).
