# Supplementary material for: Aging and metabolism contribute separately to brain–body health
Source: PLoS Biol. 2026 Jun 15;24(6):e3003856. doi: 10.1371/journal.pbio.3003856 (PMC13293518; doi:10.1371/journal.pbio.3003856)
Supplement: S2 Fig — Relationship between participants’ age at the time of brain imaging (x-axis) and raw biomarker values (y-axis) (blue: male, red: female). For biomarkers measured at both initial assessment and follow-up imaging visits, values corresponding to the imaging visit are denoted with the subscript “2”. (PDF) [file pbio.3003856.s002.pdf]

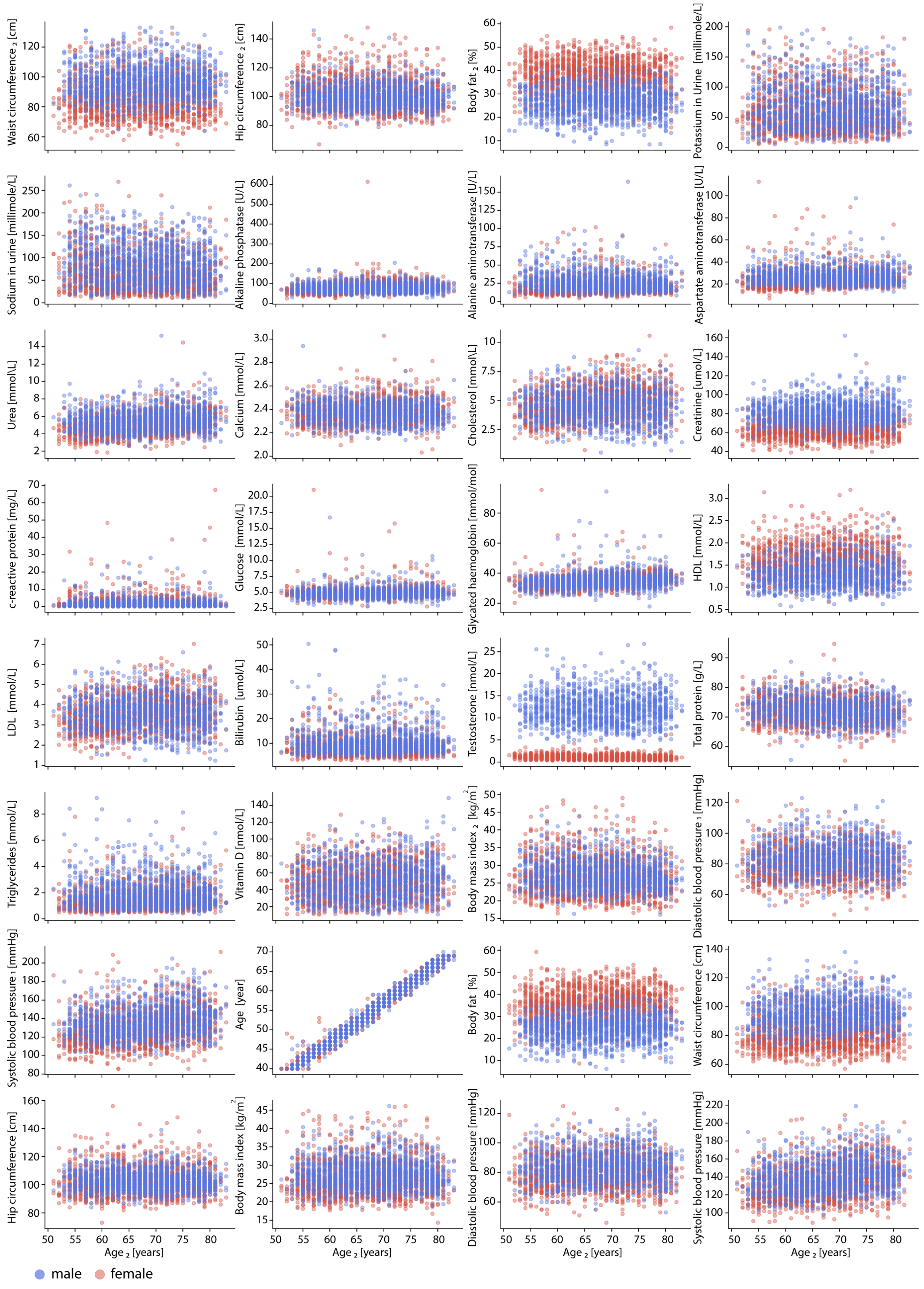

Figure S2. **UK Biobank biomarkers versus age.** Relationship between participants' age at the time of brain imaging (x-axis) and raw biomarker values (y-axis) (blue: male, red: female). For biomarkers measured at both initial assessment and follow-up imaging visits, values corresponding to the imaging visit are denoted with the subscript "2".
