# Supplementary material for: Aging and metabolism contribute separately to brain–body health
Source: PLoS Biol. 2026 Jun 15;24(6):e3003856. doi: 10.1371/journal.pbio.3003856 (PMC13293518; doi:10.1371/journal.pbio.3003856)
Supplement: S4 Fig — For each biological sex, we computed pairwise Spearman correlations between biomarkers across individuals. (PDF) [file pbio.3003856.s004.pdf]

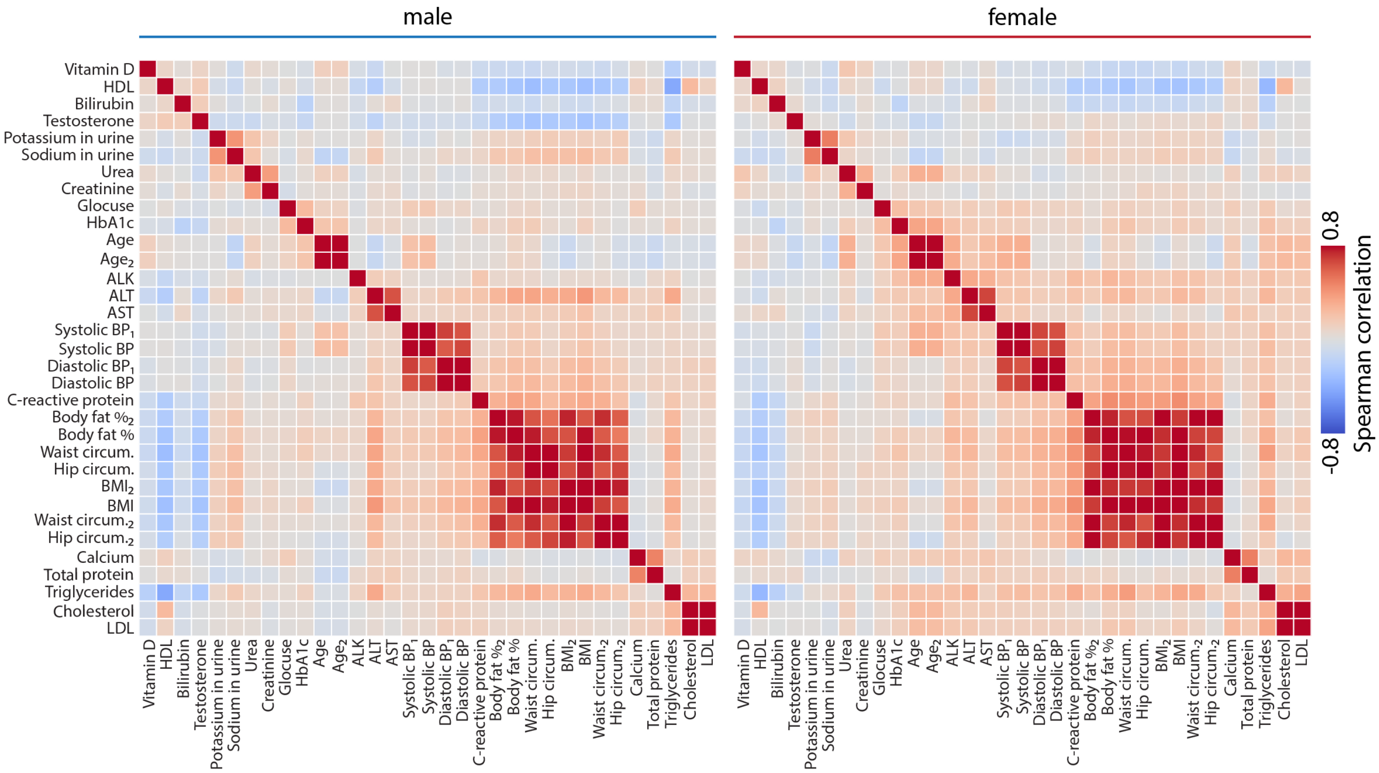

Figure S4. **Similarity of biomarkers across individuals in the UK Biobank dataset.** For each biological sex, we computed pairwise Spearman correlations between biomarkers across individuals.
