# Supplementary material for: Aging and metabolism contribute separately to brain–body health
Source: PLoS Biol. 2026 Jun 15;24(6):e3003856. doi: 10.1371/journal.pbio.3003856 (PMC13293518; doi:10.1371/journal.pbio.3003856)
Supplement: S7 Fig — (a) The heatmaps show similarity of first latent variable brain loadings across brain measurements. Associations that remain significant after controlling for spatial autocorrelation (using variogram-estimating null models) and multiple comparison correction—using false discovery rate (FDR)—are marked with their corresponding correlation values (ATT and MD: pSMASH = 0.014 (males), pSMASH=6.99×10^−3^ (females); FA and MD: pSMASH=9.32×10^−3^ (males), pSMASH=6.99×10^−3^ (females); MD and thickness: pSMASH=9.32×10^−3^ (males), pSMASH=6.99×10^−3^ (females); ATT and thickness: pSMASH = 0.04 (males), pSMASH=6.99×10^−3^ (females); perfusion and ATT: pSMASH=9.32×10^−3^ (males), pSMASH=3.15×10^−2^ (females); ATT and FA: pSMASH = 0.04 (males); myelin and SC: pSMASH = 0.04 (males); perfusion and thickness: pSMASH=1.68×10^−2^ (females); perfusion and FC: pSMASH = 0.02 (females); perfusion and MD: pSMASH=1.86×10^−2^ (females); thickness and FC: pSMASH=4.66×10^−2^ (females)). (b) The heatmaps show similarity of second latent variable brain loadings across brain measurements (FA and MD: pSMASH=6.99×10^−3^ (males), pSMASH = 0.014 (females); FA and SC: pSMASH=6.99×10^−3^ (males), pSMASH = 0.014 (females); myelin and SC: pSMASH=6.99×10^−3^ (males); ATT and thickness: pSMASH=6.99×10^−3^ (males)). (PDF) [file pbio.3003856.s007.pdf]

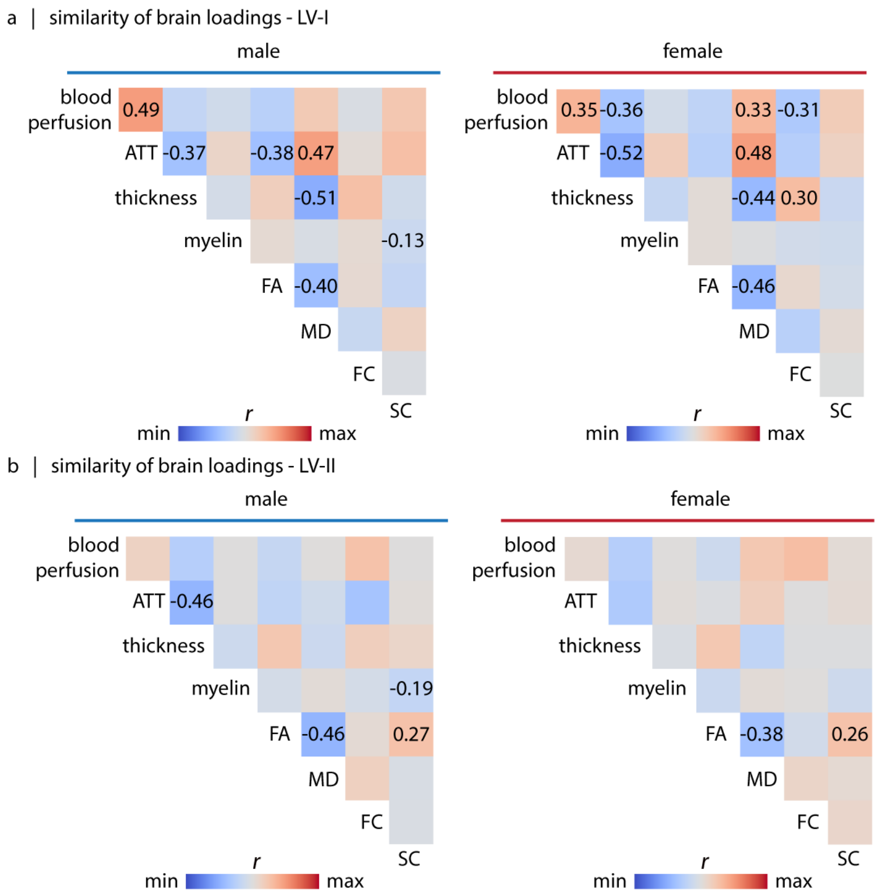

Figure S7. **Similarity of brain loadings for each latent variable.** (a) The heatmaps show similarity of first latent variable brain loadings across brain measurements. Associations that remain significant after controlling for spatial autocorrelation (using variogram-estimating null models) and multiple comparison correction—using false discovery rate (FDR)—are marked with their corresponding correlation values (ATT and MD: $p_{\text{SMASH}} = 0.014$ (males), $p_{\text{SMASH}} = 6.99 \times 10^{-3}$ (females); FA and MD: $p_{\text{SMASH}} = 9.32 \times 10^{-3}$ (males), $p_{\text{SMASH}} = 6.99 \times 10^{-3}$ (females); MD and thickness: $p_{\text{SMASH}} = 9.32 \times 10^{-3}$ (males), $p_{\text{SMASH}} = 6.99 \times 10^{-3}$ (females); ATT and thickness: $p_{\text{SMASH}} = 0.04$ (males), $p_{\text{SMASH}} = 6.99 \times 10^{-3}$ (females); perfusion and ATT: $p_{\text{SMASH}} = 9.32 \times 10^{-3}$ (males), $p_{\text{SMASH}} = 3.15 \times 10^{-2}$ (females); ATT and FA: $p_{\text{SMASH}} = 0.04$ (males); myelin and SC: $p_{\text{SMASH}} = 0.04$ (males); perfusion and thickness: $p_{\text{SMASH}} = 1.68 \times 10^{-2}$ (females); perfusion and FC: $p_{\text{SMASH}} = 0.02$ (females); perfusion and MD: $p_{\text{SMASH}} = 1.86 \times 10^{-2}$ (females); thickness and FC: $p_{\text{SMASH}} = 4.66 \times 10^{-2}$ (females)). (b) The heatmaps show similarity of second latent variable brain loadings across brain measurements (FA and MD: $p_{\text{SMASH}} = 6.99 \times 10^{-3}$ (males), $p_{\text{SMASH}} = 0.014$ (females); FA and SC: $p_{\text{SMASH}} = 6.99 \times 10^{-3}$ (males), $p_{\text{SMASH}} = 0.014$ (females); myelin and SC: $p_{\text{SMASH}} = 6.99 \times 10^{-3}$ (males); ATT and thickness: $p_{\text{SMASH}} = 6.99 \times 10^{-3}$ (males)).
