# Supplementary material for: Aging and metabolism contribute separately to brain–body health
Source: PLoS Biol. 2026 Jun 15;24(6):e3003856. doi: 10.1371/journal.pbio.3003856 (PMC13293518; doi:10.1371/journal.pbio.3003856)
Supplement: S10 Fig — Brain loadings are shown on both right and left hemispheres. Loadings are shown on the fsLR inflated cortical surfaces. (PDF) [file pbio.3003856.s010.pdf]

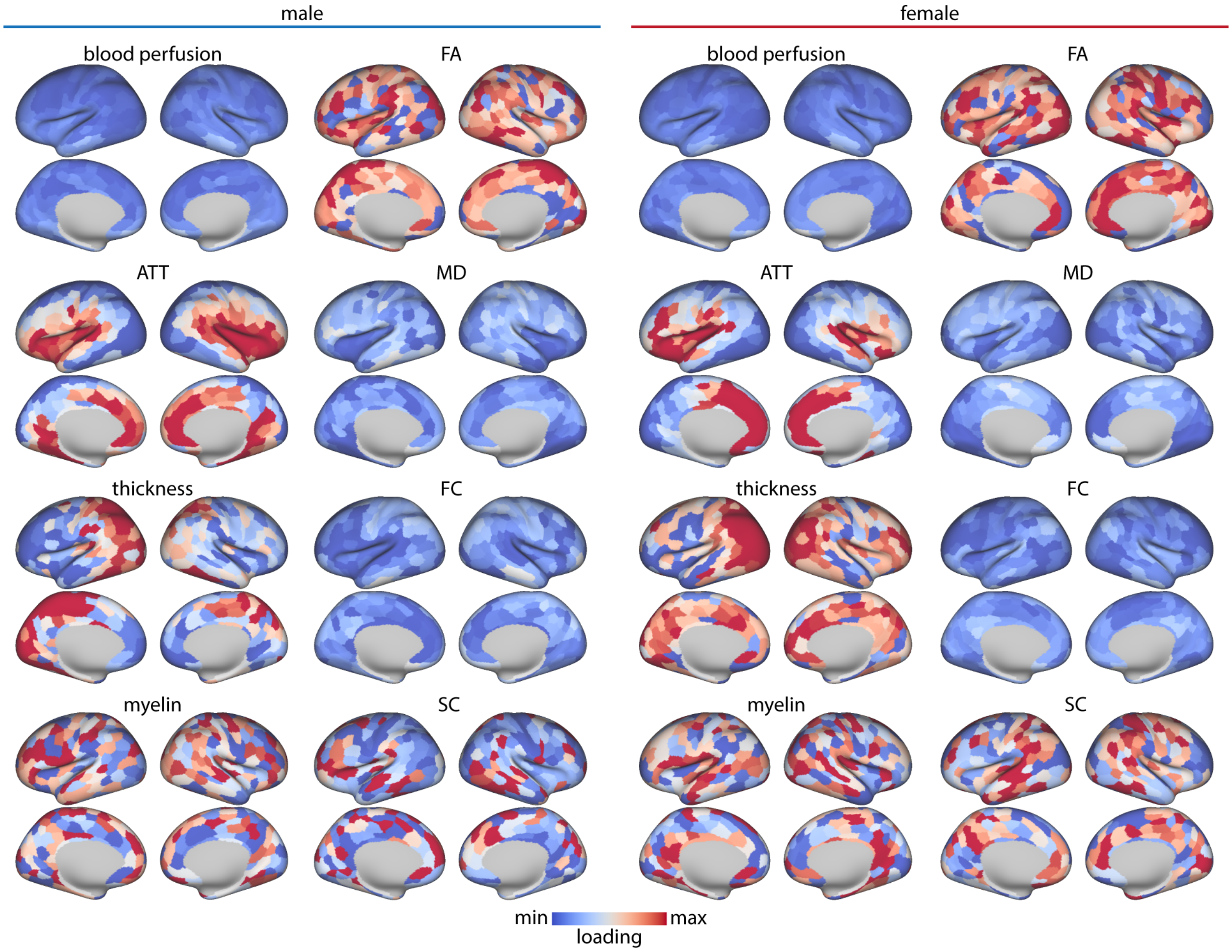

Figure S10. **LV–II Brain loadings.** Brain loadings are shown on both right and left hemispheres. Loadings are shown on the fsLR inflated cortical surfaces.
