# Supplementary material for: Aging and metabolism contribute separately to brain–body health
Source: PLoS Biol. 2026 Jun 15;24(6):e3003856. doi: 10.1371/journal.pbio.3003856 (PMC13293518; doi:10.1371/journal.pbio.3003856)
Supplement: S11 Fig — (a) Cortical brain loadings comparison. In the scatter plots, each dot represents a cortical brain region defined by the Schaefer-400 parcellation; the dots are color-coded based on the Yeo-7 functional resting-state networks. The x-axis shows male brain loadings and the y-axis shows female brain loadings. Green asterisks indicate significant associations where the significance of empirical correlation is assessed using spin tests (pspin<0.05) and orange asterisks indicate significant associations where the significance of empirical correlation is assessed using variogram-estimating null models (pSMASH<0.05). (b) White matter tract loadings comparison. The scatter plots show the similarity of brain loadings between males (x-axis) and females (y-axis) for white matter tract measures, including blood perfusion, arterial transit time (ATT), fractional anisotropy (FA), and mean diffusivity (MD). (c) White matter hyperintensity loadings comparison. The scatter plot shows the similarity of brain loadings between males (x-axis) and females (y-axis) for white matter hyperintensity measure. (PDF) [file pbio.3003856.s011.pdf]

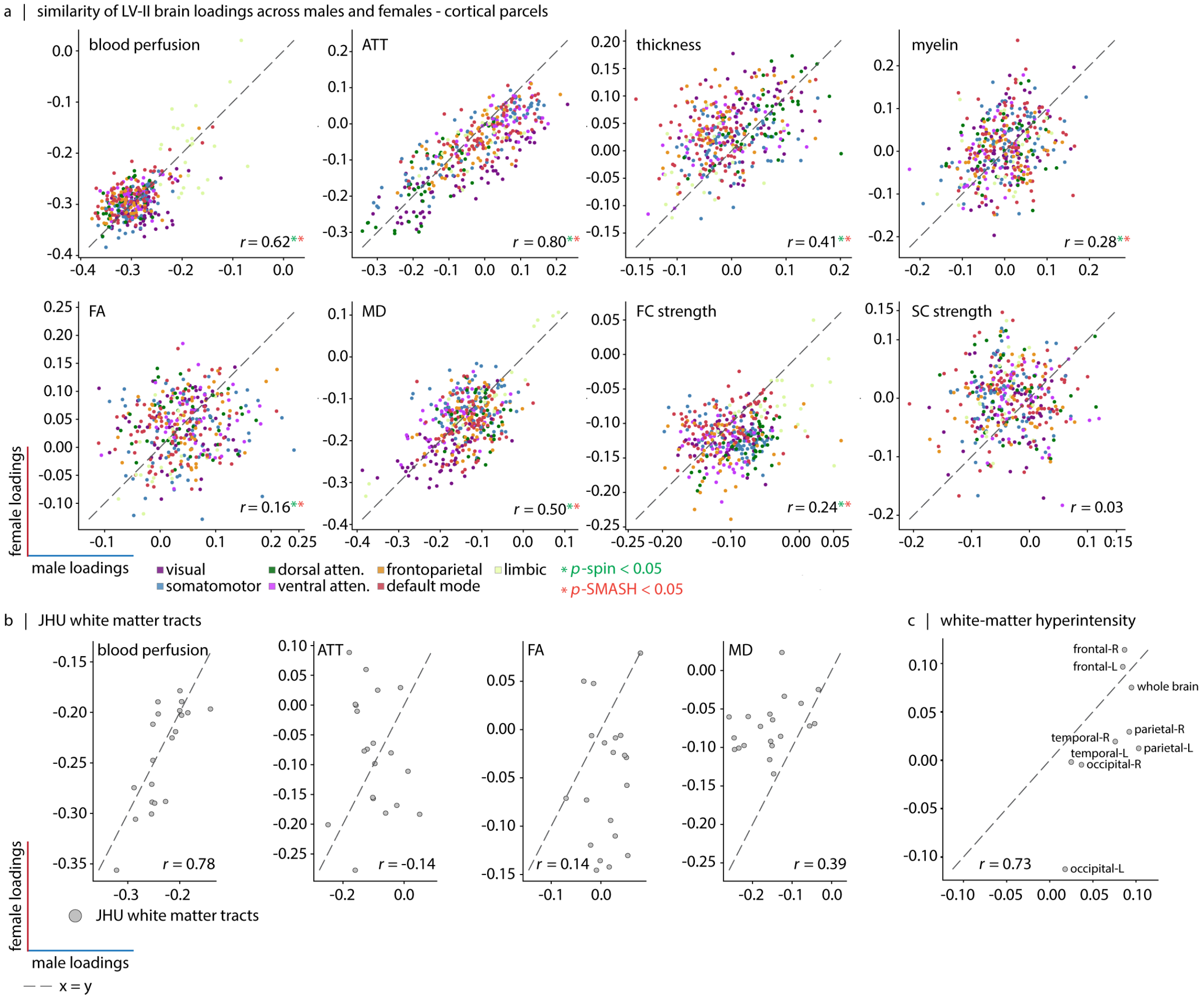

Figure S11. **Similarity of second latent variable brain loadings between males and females.** (a) Cortical brain loadings comparison. In the scatter plots, each dot represents a cortical brain region defined by the Schaefer-400 parcellation; the dots are color-coded based on the Yeo-7 functional resting-state networks. The x-axis shows male brain loadings and the y-axis shows female brain loadings. Green asterisks indicate significant associations where the significance of empirical correlation is assessed using spin tests ($p_{\text{spin}} < 0.05$) and orange asterisks indicate significant associations where the significance of empirical correlation is assessed using variogram-estimating null models ($p_{\text{SMASH}} < 0.05$). (b) White matter tract loadings comparison. The scatter plots show the similarity of brain loadings between males (x-axis) and females (y-axis) for white matter tract measures, including blood perfusion, arterial transit time (ATT), fractional anisotropy (FA), and mean diffusivity (MD). (c) White matter hyperintensity loadings comparison. The scatter plot shows the similarity of brain loadings between males (x-axis) and females (y-axis) for white matter hyperintensity measure.
