# Supplementary material for: Aging and metabolism contribute separately to brain–body health
Source: PLoS Biol. 2026 Jun 15;24(6):e3003856. doi: 10.1371/journal.pbio.3003856 (PMC13293518; doi:10.1371/journal.pbio.3003856)
Supplement: S12 Fig — (a) Brain loadings for each included brain feature. This indicates that participants who more strongly express the biomarker pattern illustrated in Fig 5A (e.g., those with greater BMI) have lower blood perfusion. Brain maps are shown on the fsLR inflated cortical surfaces. (b) Similarity of brain loadings across included brain features. Associations that remain significant after controlling for spatial autocorrelation and false discovery rate (FDR) correction are marked with their corresponding correlation values. Green asterisks indicate significant associations where the significance of empirical correlation is assessed using spin tests (pspin<0.05) and orange asterisks indicate significant associations where the significance of empirical correlation is assessed using variogram-estimating null models (pSMASH<0.05) (FC and blood perfusion: pspin = 0.042, pSMASH>0.05 (males), pspin=1.86×10^−2^, pSMASH>0.05 (females); FA and MD: pspin=6.99×10^−3^, pSMASH=9.32×10^−3^ (males), pspin = 0.014, pSMASH = 0.014 (females); FA and SC: pspin=6.99×10^−3^, pSMASH=9.32×10^−3^ (males), pspin = 0.014, pSMASH = 0.014 (females); myelin and SC: pspin=6.99×10^−3^, pSMASH=9.32×10^−3^ (males); ATT and FC: pspin=6.99×10^−3^, pSMASH>0.05 (males); thickness and myelin: pspin = 0.042, pSMASH>0.05 (males); thickness and FA: pspin = 0.042, pSMASH>0.05 (females); ATT and thickness: pspin>0.05, pSMASH = 0.021 (males)). (PDF) [file pbio.3003856.s012.pdf]

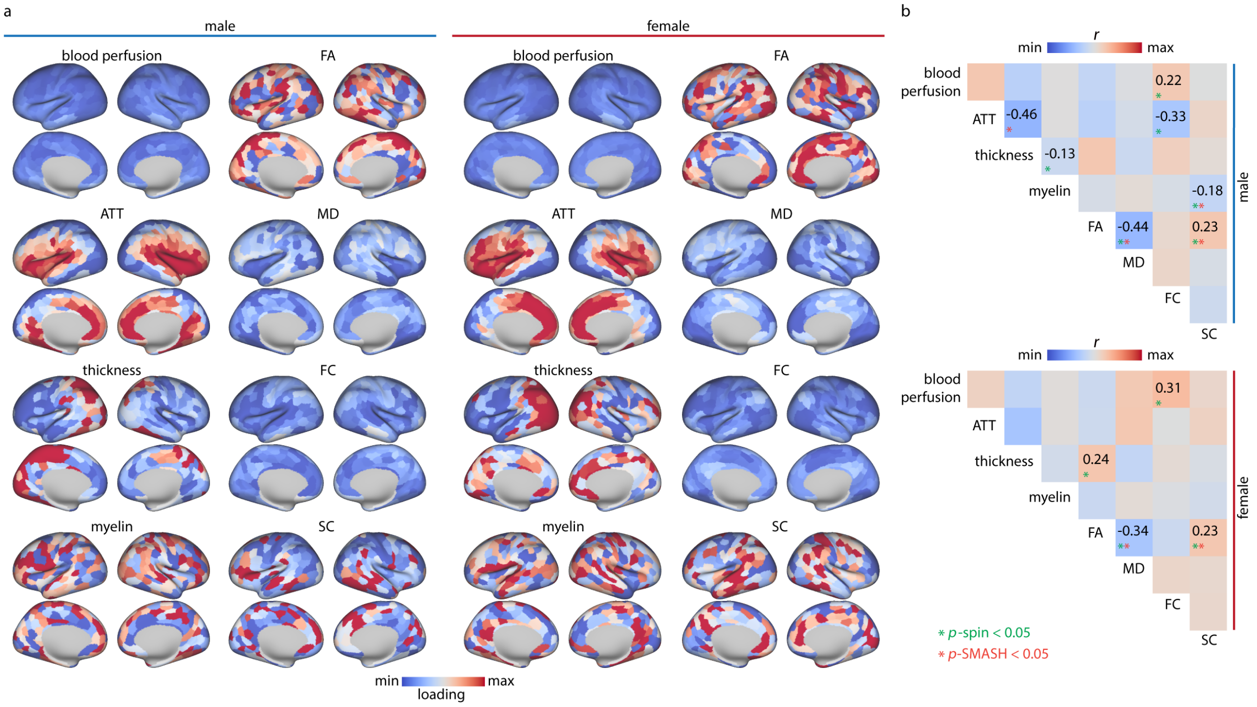

Figure S12. **LV–I′ brain loadings - no age effect.** (a) Brain loadings for each included brain feature. This indicates that participants who more strongly express the biomarker pattern illustrated in Fig 5A (e.g., those with greater BMI) have lower blood perfusion. Brain maps are shown on the fsLR inflated cortical surfaces. (b) Similarity of brain loadings across included brain features. Associations that remain significant after controlling for spatial autocorrelation and false discovery rate (FDR) correction are marked with their corresponding correlation values. Green asterisks indicate significant associations where the significance of empirical correlation is assessed using spin tests ($p_{\text{spin}} < 0.05$) and orange asterisks indicate significant associations where the significance of empirical correlation is assessed using variogram-estimating null models ($p_{\text{SMASH}} < 0.05$) (FC and blood perfusion: $p_{\text{spin}} = 0.042$, $p_{\text{SMASH}} > 0.05$ (males), $p_{\text{spin}} = 1.86 \times 10^{-2}$, $p_{\text{SMASH}} > 0.05$ (females); FA and MD: $p_{\text{spin}} = 6.99 \times 10^{-3}$, $p_{\text{SMASH}} = 9.32 \times 10^{-3}$ (males), $p_{\text{spin}} = 0.014$, $p_{\text{SMASH}} = 0.014$ (females); FA and SC: $p_{\text{spin}} = 6.99 \times 10^{-3}$, $p_{\text{SMASH}} = 9.32 \times 10^{-3}$ (males), $p_{\text{spin}} = 0.014$, $p_{\text{SMASH}} = 0.014$ (females); myelin and SC: $p_{\text{spin}} = 6.99 \times 10^{-3}$, $p_{\text{SMASH}} = 9.32 \times 10^{-3}$ (males); ATT and FC: $p_{\text{spin}} = 6.99 \times 10^{-3}$, $p_{\text{SMASH}} > 0.05$ (males); thickness and myelin: $p_{\text{spin}} = 0.042$, $p_{\text{SMASH}} > 0.05$ (males); thickness and FA: $p_{\text{spin}} = 0.042$, $p_{\text{SMASH}} > 0.05$ (females); ATT and thickness: $p_{\text{spin}} > 0.05$, $p_{\text{SMASH}} = 0.021$ (males)).
