# Supplementary material for: Aging and metabolism contribute separately to brain–body health
Source: PLoS Biol. 2026 Jun 15;24(6):e3003856. doi: 10.1371/journal.pbio.3003856 (PMC13293518; doi:10.1371/journal.pbio.3003856)
Supplement: S19 Fig — Using PLS analysis, we identify a significant latent variable that accounts for 65.31% (males) and 60.37% (females) of the covariance in the data. The PLS model includes 33 features on the biomarker side and 490 features on the brain side. (a) Biomarker loadings. Bootstrap resampling is used to estimate the stability of each individual biomarker’s contribution to the overall multivariate pattern. Each biomarker loading is divided by its bootstrap-estimated standard error, yielding a measure called “bootstrap ratio”. Bootstrap ratio is high for biomarkers with large weights and small standard errors. Stable biomarkers for which the estimated 95% confidence intervals do not cross zero, are shown in red. BMI, body fat percentage, age, hip and waist circumference were collected at both initial assessment and imaging visits. Variables measured in the imaging visit are denoted with a subscript 2 (e.g., BMI2), while baseline values are written without subscripts. Subscript 1 is used to indicate repeated measurements (e.g., systolic blood pressure1). (b) Brain loadings. Each dot represents a brain region (cortical, subcortical, or a white matter tract). (c) Correlation between brain (x-axis) and biomarker scores (y-axis) for males (top; r = 0.40) and females (bottom; r = 0.38). Each dot represents an individual participant, colored by their BMI at imaging visit. The score per participant shows the extent to which the participant expresses the brain–biomarker association captured by LV–I ′. Score correlation values passed cross-validation in both sex groups. (PDF) [file pbio.3003856.s019.pdf]

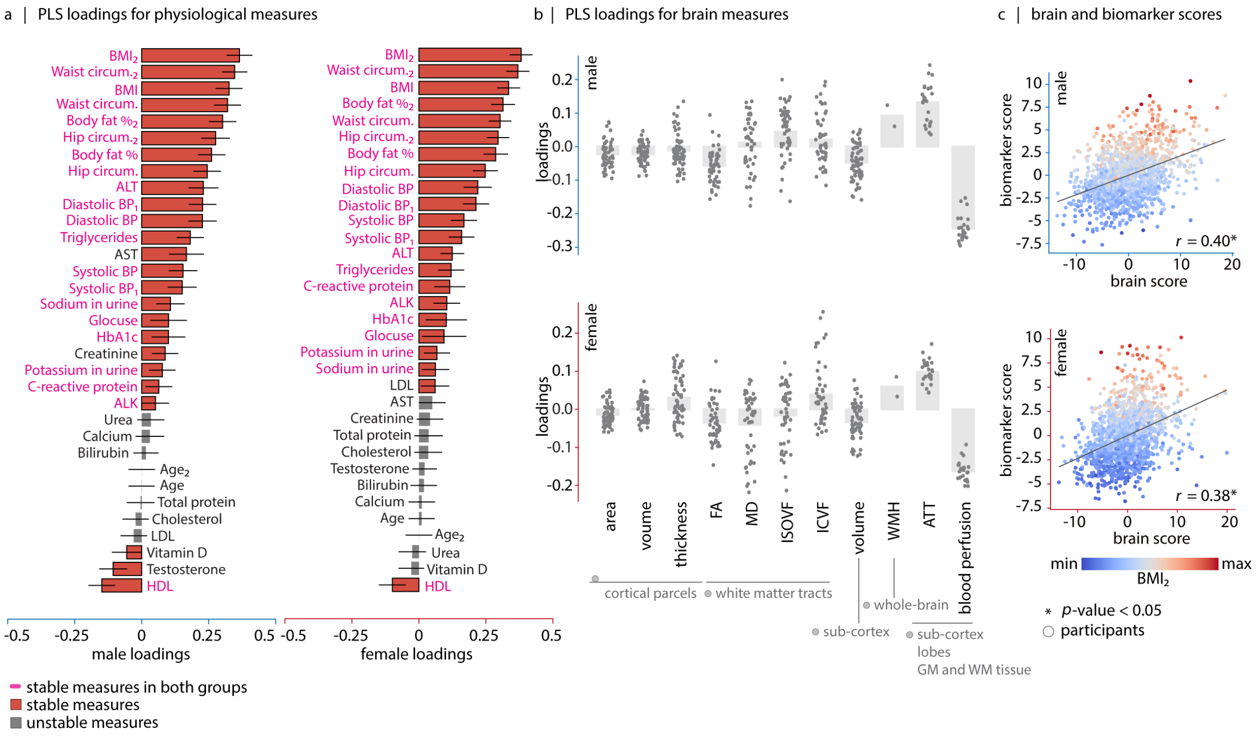

Figure S19. **Mapping biomarkers to brain features after regressing out the age effect in the UK Biobank dataset: first latent variable (LV–I′) captures the metabolic axis.** Using PLS analysis, we identify a significant latent variable that accounts for 65.31% (males) and 60.37% (females) of the covariance in the data. The PLS model includes 33 features on the biomarker side and 490 features on the brain side. (a) Biomarker loadings. Bootstrap resampling is used to estimate the stability of each individual biomarker's contribution to the overall multivariate pattern. Each biomarker loading is divided by its bootstrap-estimated standard error, yielding a measure called "bootstrap ratio". Bootstrap ratio is high for biomarkers with large weights and small standard errors. Stable biomarkers for which the estimated 95% confidence intervals do not cross zero, are shown in red. BMI, body fat percentage, age, hip and waist circumference were collected at both initial assessment and imaging visits. Variables measured in the imaging visit are denoted with a subscript $_2$ (e.g., BMI_2), while baseline values are written without subscripts. Subscript $_1$ is used to indicate repeated measurements (e.g., systolic blood pressure$_1$). (b) Brain loadings. Each dot represents a brain region (cortical, subcortical, or a white matter tract). (c) Correlation between brain (x-axis) and biomarker scores (y-axis) for males (top; $r = 0.40$) and females (bottom; $r = 0.38$). Each dot represents an individual participant, colored by their BMI at imaging visit. The score per participant shows the extent to which the participant expresses the brain–biomarker association captured by LV–I′. Score correlation values passed cross-validation in both sex groups.
