# Supplementary material for: Aging and metabolism contribute separately to brain–body health
Source: PLoS Biol. 2026 Jun 15;24(6):e3003856. doi: 10.1371/journal.pbio.3003856 (PMC13293518; doi:10.1371/journal.pbio.3003856)
Supplement: S20 Fig — Blood perfusion loadings have the greatest absolute values among all brain measures examined. Horizontal lines indicate statistical significance (p < 0.05, FDR-corrected). Black dots represent mean absolute loading values. Also see S19B Fig. (PDF) [file pbio.3003856.s020.pdf]

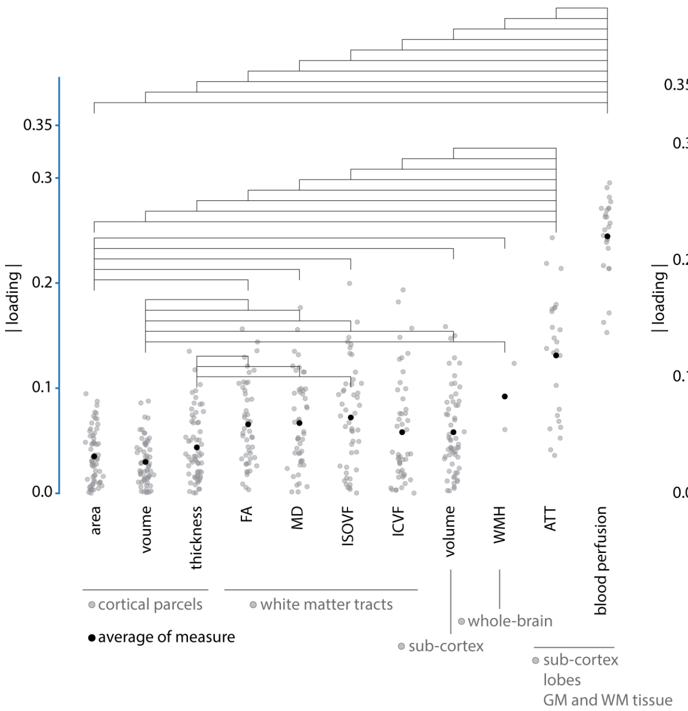

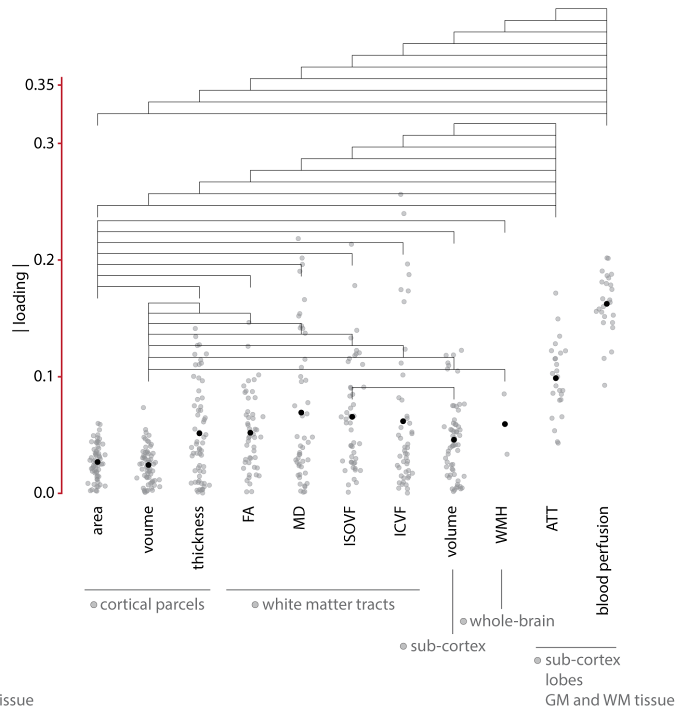

Figure S20. **Statistical comparison of absolute brain loading values for the first latent variable in UK Biobank - no age effect.** Blood perfusion loadings have the greatest absolute values among all brain measures examined. Horizontal lines indicate statistical significance ($p < 0.05$, FDR-corrected). Black dots represent mean absolute loading values. Also see Fig S19B.
