# Supplementary material for: Aging and metabolism contribute separately to brain–body health
Source: PLoS Biol. 2026 Jun 15;24(6):e3003856. doi: 10.1371/journal.pbio.3003856 (PMC13293518; doi:10.1371/journal.pbio.3003856)
Supplement: S21 Fig — The left scatter plot shows data for males (pperm=1.50×10^−2^) and the right scatter plot shows data for females (pperm=9.99×10^−4^) (Nperm = 1,000). Brain scores are derived from the analysis presented in S19 Fig. Fitted regression lines are shown in blue for males and in red for females. (PDF) [file pbio.3003856.s021.pdf]

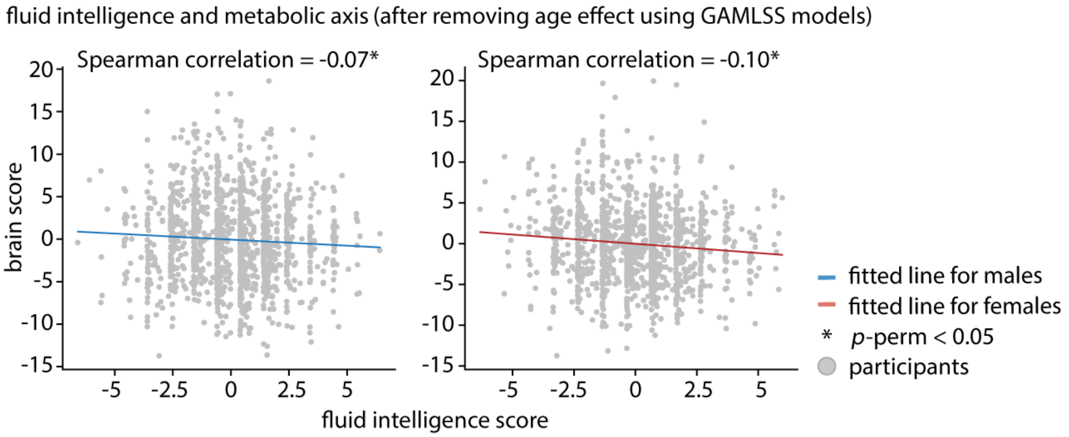

Figure S21. **Cognitive relevance of metabolic axis in UK Biobank.** The left scatter plot shows data for males ($p_{\text{perm}} = 1.50 \times 10^{-2}$) and the right scatter plot shows data for females ($p_{\text{perm}} = 9.99 \times 10^{-4}$) ($N_{\text{perm}} = 1\,000$). Brain scores are derived from the analysis presented in Fig S19. Fitted regression lines are shown in blue for males and in red for females.
