# Supplementary material for: Aging and metabolism contribute separately to brain–body health
Source: PLoS Biol. 2026 Jun 15;24(6):e3003856. doi: 10.1371/journal.pbio.3003856 (PMC13293518; doi:10.1371/journal.pbio.3003856)
Supplement: S22 Fig — We identify brain features and biomarkers shared across the HCP–A and UK Biobank datasets. We use these features to construct sex-stratified PLS models based on the HCP–A data. (a) Biomarker loadings of the first latent variable. Bootstrap resampling is used to estimate the stability of each individual biomarker’s contribution to the overall multivariate pattern. Stable biomarkers are shown in red. (b) Brain loadings of the first latent variable. Each bar represents a global brain feature. (c) Using the weights of the PLS models, we mapped the UK Biobank data into the HCP–A-derived latent space. For the first latent variable, significant correlations between projected brain and biomarker scores are observed for males (top; r = 0.41, pperm=9.999×10^−5^) and females (bottom; r = 0.46, pperm=9.999×10^−5^). (d) Biomarker loadings of the second latent variable. (e) Brain loadings of the second latent variable. (f) Using the weights of the PLS models, we mapped the UK Biobank data into the HCP–A-derived latent space. For the second latent variable, significant correlations between projected brain and biomarker scores are observed for males (top; r = 0.12, pperm=9.999×10^−5^) and females (bottom; r = 0.13, pperm=9.999×10^−5^). (PDF) [file pbio.3003856.s022.pdf]

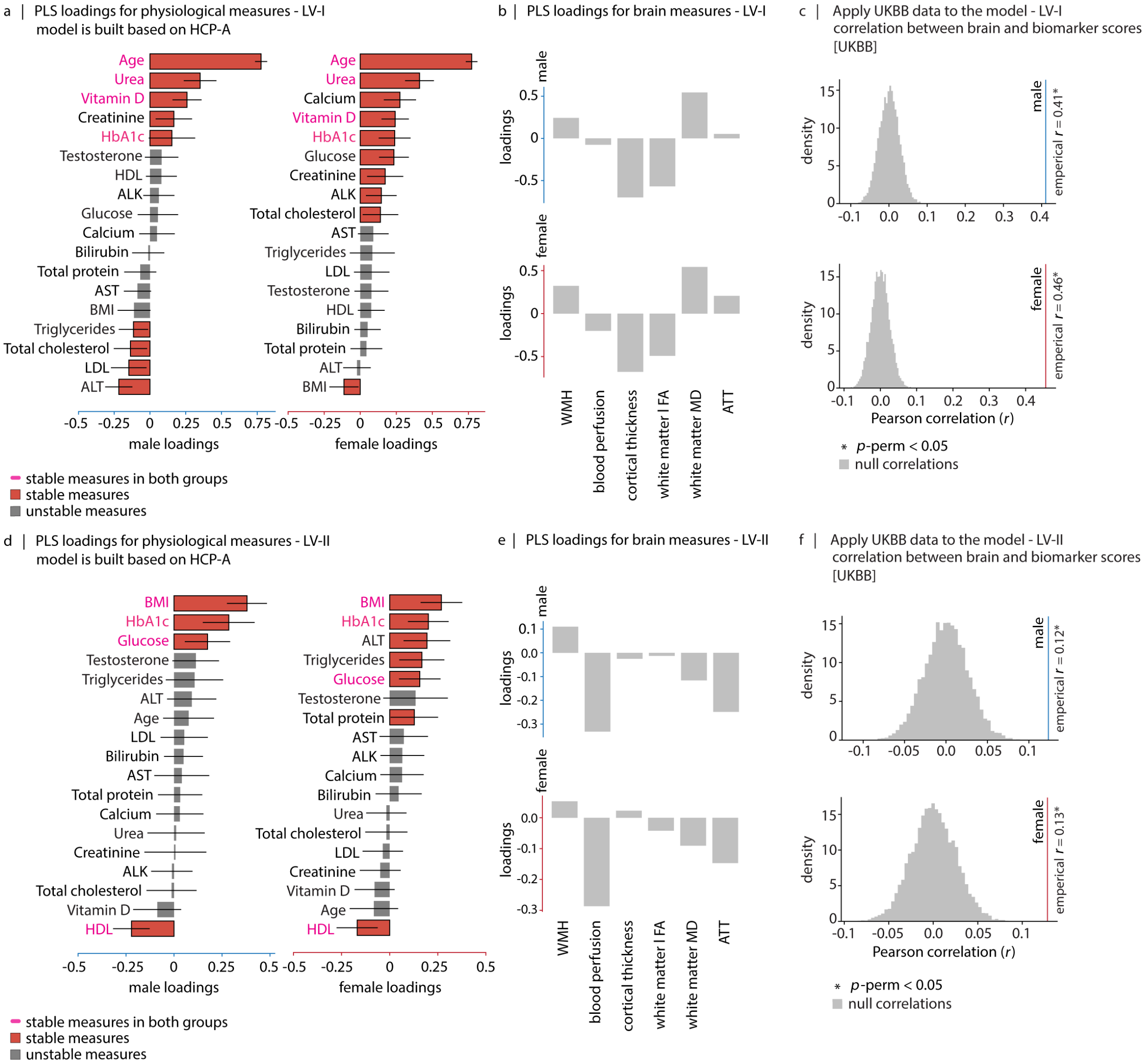

Figure S22. **Generalization of HCP–A-derived PLS latent variables to the UK Biobank cohort.** We identify brain features and biomarkers shared across the HCP–A and UK Biobank datasets. We use these features to construct sex-stratified PLS models based on the HCP–A data. (a) Biomarker loadings of the first latent variable. Bootstrap resampling is used to estimate the stability of each individual biomarker's contribution to the overall multivariate pattern. Stable biomarkers are shown in red. (b) Brain loadings of the first latent variable. Each bar represents a global brain feature. (c) Using the weights of the PLS models, we mapped the UK Biobank data into the HCP–A-derived latent space. For the first latent variable, significant correlations between projected brain and biomarker scores are observed for males (top; $r = 0.41$, $p_{\text{perm}} = 9.999 \times 10^{-5}$) and females (bottom; $r = 0.46$, $p_{\text{perm}} = 9.999 \times 10^{-5}$). (d) Biomarker loadings of the second latent variable. (e) Brain loadings of the second latent variable. (f) Using the weights of the PLS models, we mapped the UK Biobank data into the HCP–A-derived latent space. For the second latent variable, significant correlations between projected brain and biomarker scores are observed for males (top; $r = 0.12$, $p_{\text{perm}} = 9.999 \times 10^{-5}$) and females (bottom; $r = 0.13$, $p_{\text{perm}} = 9.999 \times 10^{-5}$).
