# Supplementary material for: Aging and metabolism contribute separately to brain–body health
Source: PLoS Biol. 2026 Jun 15;24(6):e3003856. doi: 10.1371/journal.pbio.3003856 (PMC13293518; doi:10.1371/journal.pbio.3003856)
Supplement: S24 Fig — Using PLS analysis, we identify a significant latent variable that accounts for 10.29% (males) and 10.79% (females) of the covariance between brain measurements and biomarkers. The PLS model includes 28 features on the biomarker side and 13 features on the brain side. (a) Biomarker loadings. Bootstrap resampling is used to estimate the stability of each individual biomarker’s contribution to the overall multivariate pattern. Stable biomarkers for which the estimated 95% confidence intervals do not cross zero, are shown in red. (b) Brain loadings. Each bar represents a global brain measure. (c) Correlation between brain (x-axis) and biomarker scores (y-axis) for males (top; r = 0.48) and females (bottom; r = 0.50). Each dot represents an individual participant, colored by their BMI. Score correlation values passed cross-validation in both sex groups (for both: p=9.90×10^−3^). (PDF) [file pbio.3003856.s024.pdf]

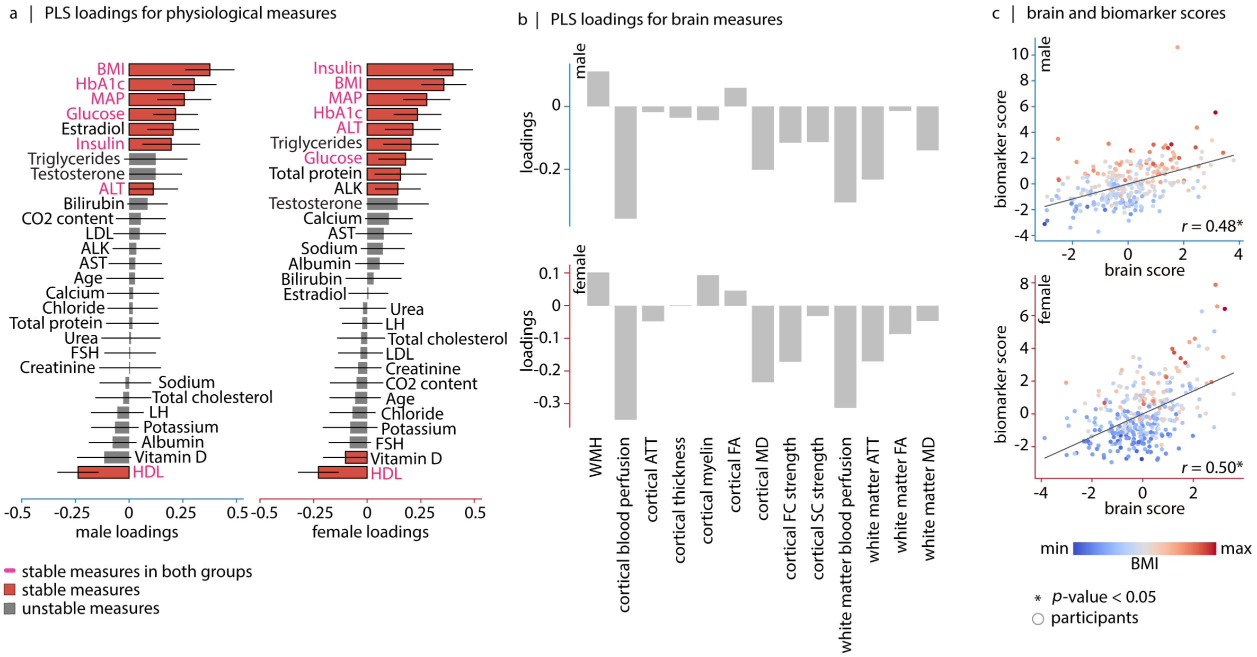

Figure S24. **Mapping biomarkers to reduced brain features in the HCP–A dataset: second latent variable (LV–II) captures the metabolic axis.** Using PLS analysis, we identify a significant latent variable that accounts for 10.29% (males) and 10.79% (females) of the covariance between brain measurements and biomarkers. The PLS model includes 28 features on the biomarker side and 13 features on the brain side. (a) Biomarker loadings. Bootstrap resampling is used to estimate the stability of each individual biomarker's contribution to the overall multivariate pattern. Stable biomarkers for which the estimated 95% confidence intervals do not cross zero, are shown in red. (b) Brain loadings. Each bar represents a global brain measure. (c) Correlation between brain (x-axis) and biomarker scores (y-axis) for males (top; $r = 0.48$) and females (bottom; $r = 0.50$). Each dot represents an individual participant, colored by their BMI. Score correlation values passed cross-validation in both sex groups (for both: $p = 9.90 \times 10^{-3}$).
