# Supplementary material for: Aging and metabolism contribute separately to brain–body health
Source: PLoS Biol. 2026 Jun 15;24(6):e3003856. doi: 10.1371/journal.pbio.3003856 (PMC13293518; doi:10.1371/journal.pbio.3003856)
Supplement: S27 Fig — Not all individuals exposed to metabolic dysfunction experience equivalent brain outcomes. (a) Brain and biomarker scores for LV–II in HCP–A, (b) and in UK Biobank. Some individuals show elevated metabolic dysfunction on the biomarker side but relatively preserved or less affected brain signatures. These individuals fall below the main brain–biomarker regression line (pink dots), and represent a resilient phenotype, in which the brain appears comparatively protected relative to metabolic risk. Conversely, some individuals show adverse brain metabolic signatures despite only moderate peripheral risk (gray dots). These cases reflect a vulnerable phenotype, in which the brain is particularly sensitive to metabolic perturbations. On the leftmost panel, we highlight two male participants from the HCP–A cohort with comparable biomarker scores but divergent brain scores. The participant shown in blue has a smaller brain score and the one in red has a higher brain score, meaning that despite similar peripheral metabolic profiles, the participant shown in red has greater brain vulnerability. (PDF) [file pbio.3003856.s027.pdf]

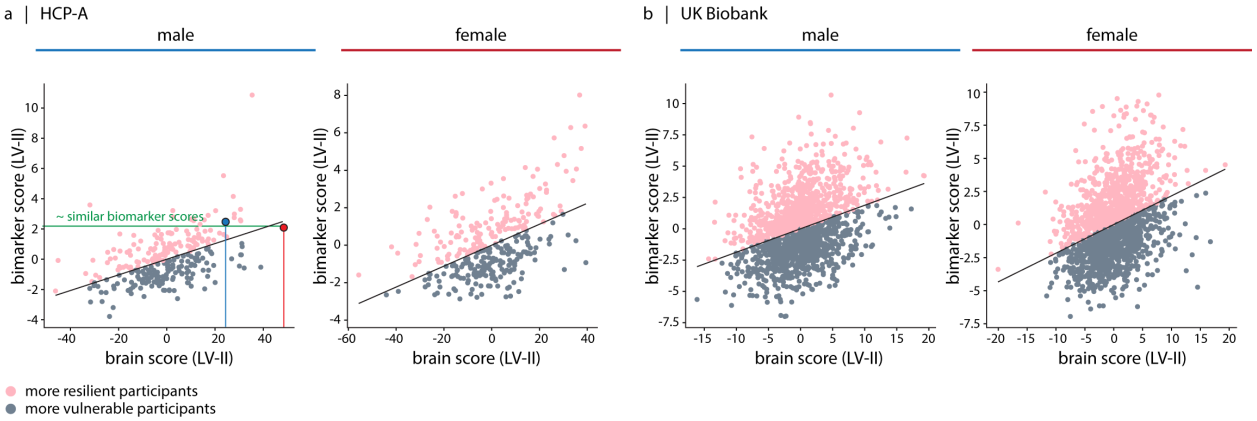

Figure S27. **Individual variability in brain outcomes in metabolic syndrome.** Not all individuals exposed to metabolic dysfunction experience equivalent brain outcomes. (a) Brain and biomarker scores for LV–II in HCP–A, (b) and in UK Biobank. Some individuals show elevated metabolic dysfunction on the biomarker side but relatively preserved or less affected brain signatures. These individuals fall below the main brain–biomarker regression line (pink dots), and represent a resilient phenotype, in which the brain appears comparatively protected relative to metabolic risk. Conversely, some individuals show adverse brain metabolic signatures despite only moderate peripheral risk (gray dots). These cases reflect a vulnerable phenotype, in which the brain is particularly sensitive to metabolic perturbations. On the leftmost panel, we highlight two male participants from the HCP–A cohort with comparable biomarker scores but divergent brain scores. The participant shown in blue has a smaller brain score and the one in red has a higher brain score, meaning that despite similar peripheral metabolic profiles, the participant shown in red has greater brain vulnerability.
