# Supplementary material for: Aging and metabolism contribute separately to brain–body health
Source: PLoS Biol. 2026 Jun 15;24(6):e3003856. doi: 10.1371/journal.pbio.3003856 (PMC13293518; doi:10.1371/journal.pbio.3003856)
Supplement: S1 Table — For each biomarker, the table reports the number of participants with missing values (not-a-number). Total number of females is 329, and total number of males is 268. (PDF) [file pbio.3003856.s028.pdf]

**Biomarker**	**Number of NaN - female**	**Number of NaN - male**
Age	0	0
Alanine aminotransferase (ALT)	0	0
Albumin	0	0
Alkaline phosphatase (ALK)	0	0
Aspartate aminotransferase (AST)	0	0
Bilirubin	0	0
Body mass index (BMI)	2	0
Calcium	0	0
Chloride	0	0
Creatinine	0	0
CO2 content	0	0
Estradiol	1	2
FSH	1	2
Glucose	0	0
Glycosylated haemoglobin (HbA1c)	3	1
HDL	0	0
Insulin	0	0
LDL	0	0
LH	1	2
Mean arterial pressure (MAP)	4	2
Potassium	0	0
Sodium	0	0
Testosterone	1	2
Total cholesterol	0	0
Total protein	0	0
Triglycerides	0	0
Urea	0	0
Vitamin D	0	0

TABLE S1. **Biomarkers in HCP–Aging.** For each biomarker, the table reports the number of participants with missing values (not-a-number). Total number of females is 329, and total number of males is 268.
