# Supplementary material for: Aging and metabolism contribute separately to brain–body health
Source: PLoS Biol. 2026 Jun 15;24(6):e3003856. doi: 10.1371/journal.pbio.3003856 (PMC13293518; doi:10.1371/journal.pbio.3003856)
Supplement: S2 Table — For each biomarker, the table reports the acquisition session and the number of participants with missing values (not-a-number). Body mass index, body fat percentage, age, hip and waist circumference were measured at both the initial assessment and the imaging visits. Variables acquired during the imaging visit are denoted as 2.0 in the “Time point” column, while values acquired during the initial assessment are denoted as 0.0. A value of 0.1 indicates values acquired during the repeated assessment visit. (PDF) [file pbio.3003856.s029.pdf]

Biomarker	Time point - instance	Number of NaN - female	Number of NaN - male
Age	0.0	0	0
Age	2.0	0	0
Alanine aminotransferase (ALT)	0.0	131	105
Alkaline phosphatase (ALK)	0.0	130	103
Aspartate aminotransferase (AST)	0.0	136	108
Bilirubin	0.0	135	110
Body fat percentage	0.0	22	8
Body fat percentage	2.0	42	55
Body mass index (BMI)	0.0	4	2
Body mass index (BMI)	2.0	7	9
C-reactive protein	0.0	136	105
Calcium	0.0	255	105
Cholesterol	0.0	131	103
Creatinine	0.0	132	105
Diastolic blood pressure	0.0	128	115
Diastolic blood pressure	0.1	154	129
Glucose	0.0	257	197
Glycosylated haemoglobin (HbA1c)	0.0	143	116
HDL	0.0	254	195
Hip circumference	0.0	2	1
Hip circumference	2.0	3	7
LDL	0.0	133	106
Potassium in urine	0.0	67	46
Sodium in urine	0.0	65	44
Systolic blood pressure	0.0	128	115
Systolic blood pressure	0.1	154	129
Testosterone	0.0	325	117
Triglycerides	0.0	132	104
Total protein	0.0	255	195
Urea	0.0	131	104
Vitamin D	0.0	181	138
Waist circumference	0.0	2	1
Waist circumference	2.0	3	7

TABLE S2. **Biomarkers in UK Biobank.** For each biomarker, the table reports the acquisition session and the number of participants with missing values (not-a-number). Body mass index, body fat percentage, age, hip and waist circumference were measured at both the initial assessment and the imaging visits. Variables acquired during the imaging visit are denoted as 2.0 in the “Time point” column, while values acquired during the initial assessment are denoted as 0.0. A value of 0.1 indicates values acquired during the repeated assessment visit.
