# Supplementary material for: Aging and metabolism contribute separately to brain–body health
Source: PLoS Biol. 2026 Jun 15;24(6):e3003856. doi: 10.1371/journal.pbio.3003856 (PMC13293518; doi:10.1371/journal.pbio.3003856)
Supplement: Attachment — Submitted filename: Response_letter.pdf [file pbio.3003856.s030.pdf]

Dear Dr. Smith and Reviewers,

Thank you for the constructive feedback on our first submission, and for the opportunity to improve the manuscript. Following your comments and suggestions, we have substantially revised the manuscript. In this letter, we respond to each of the reviewers' comments in detail. Reviewer comments are in **bold,** and our responses are in regular font.

Major changes in the manuscript include:

- **Additional robustness and sensitivity analyses:** We performed three complementary analyses to further evaluate the stability and validity of the latent variables:

    1. We reduced the dimensionality of the brain features by computing global measures for each brain imaging feature (e.g., overall cortical thickness rather than parcel-wise thickness across 400 regions).
    2. We assessed generalizability across cohorts and showed that the latent brain–biomarker profiles identified in HCP-A generalize to an independent cohort (UK Biobank).
    3. We applied sparse PLS and recovered the same dominant latent structure, indicating that the observed associations are not driven by weak or redundant features.

- **Improved figure interpretability:** We have swapped the directionality of latent variable loadings shown in the figures of the manuscript to ensure that readers follow the narrative of the paper more easily.

- **Expanded statistical reporting:** We have extended our reported statistics in the paper. Specifically, we now report p-values derived using the null model introduced by Burt *et al.* (2020), which generates variogram-matched surrogate maps and accounts for spatial autocorrelation when assessing the similarity of cortical maps.

Reviewer #1, Frauke Beyer (reviewer 1 has signed this review)

In this manuscript, Farahani et al. derive latent components describing the association of anthropometric and metabolic measures and brain imaging phenotypes. The first component pair is dominated by age and age-related metabolic changes relating to decreased structural brain integrity. The second component, independent of age, shows an association of metabolic features like HDL, obesity, mean arterial pressure, HbA1c with primarily cerebral perfusion. The patterns are consistent in men and women and across two population-based cohorts.

The manuscript is well written, the analyses are clear and can be thoroughly followed in the provided code. The results are clearly presented and discussed. I also liked that all analyses are performed sex-stratified.

I only have a few comments which may help to improve this nice manuscript.

Results:
- Throughout the manuscript, there sometimes is confusion about the direction of the axis, e.g. in the abstract you write about reduced perfusion, and what is described in this paragraph: "LV-II biomarker profile is highlighting low HDL and age, along with high BMI, hip and waist circumferences, body fat percentage, diastolic blood pressure, ALT, aspartate aminotransferase (AST), triglyceride, c-reactive protein—previously linked to metabolic syndrome and inflammatory disorders [227]—and sodium in urine. This metabolic profile is dominantly associated with lower brain blood perfusion (Fig. S14b and Fig. S15)" is shown inversely in Figure S14 (i.e. low BMI, high age...). Maybe all directions could be swapped so that higher scores in the metabolic component are associated with higher metabolic risk?

We have now swapped the directionality of latent variable loadings presented in the figures to ensure that readers follow the narrative of the paper more easily. In the following, we have included the updated versions of the main figures. The same modification has also been applied to the supplementary figures.

Figure 1:

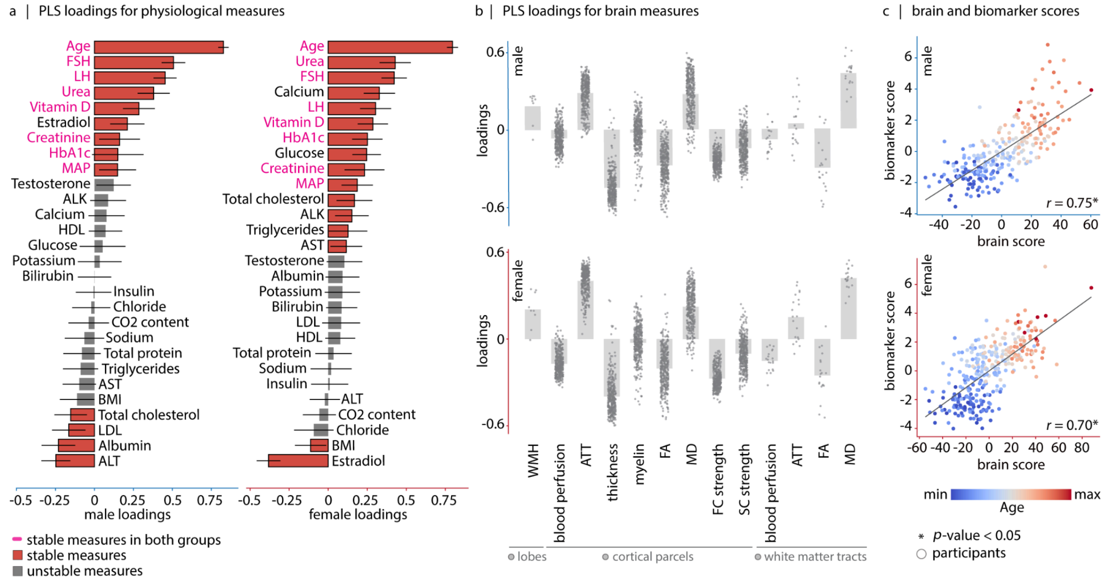

Figure 2:

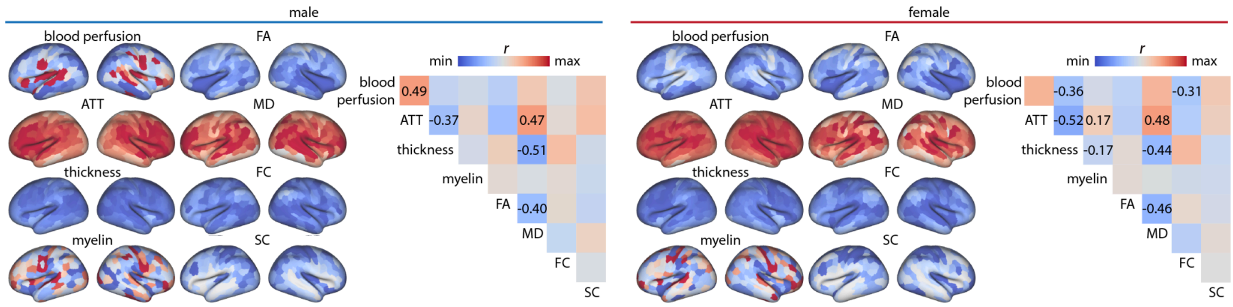

Figure 3:

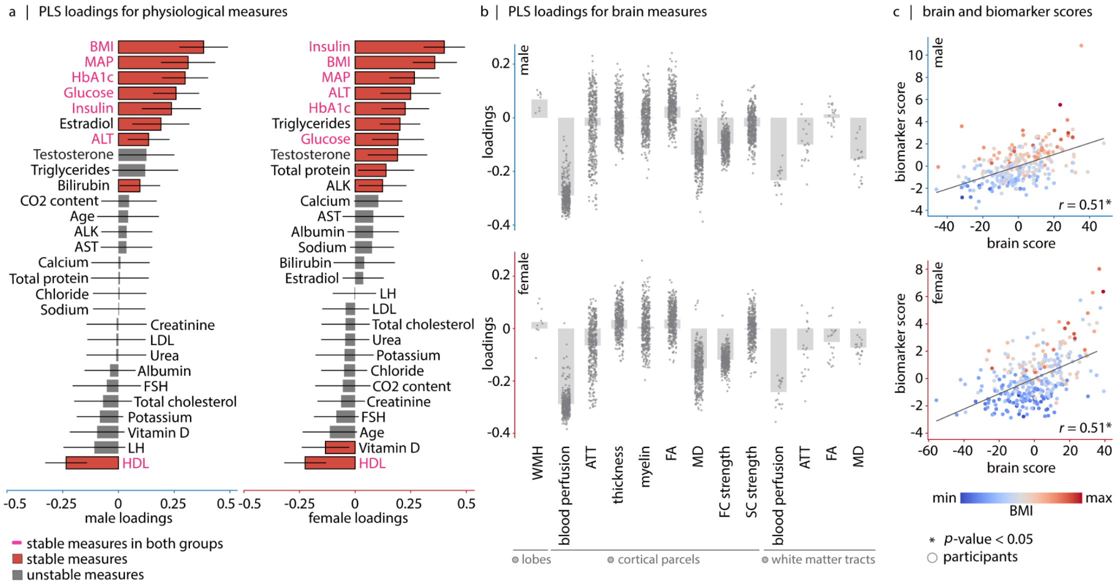

Figure 4:

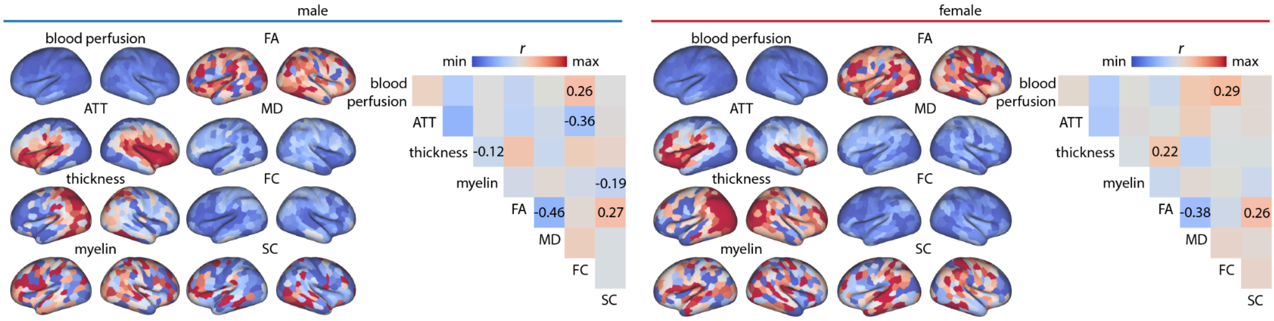

Figure 5:

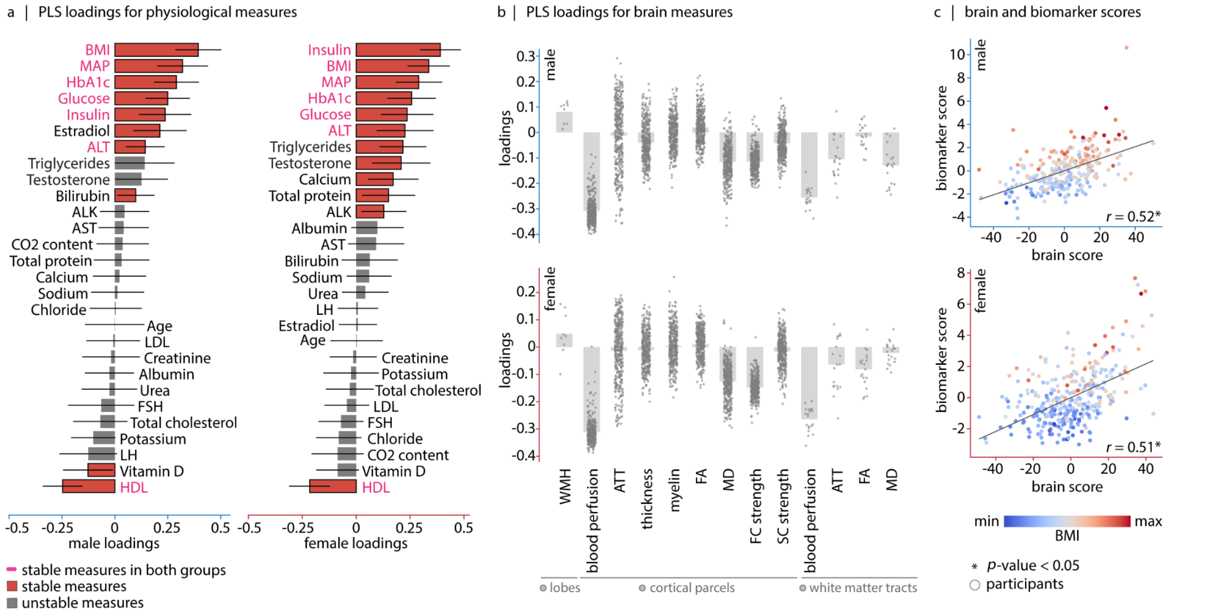

Figure 6:

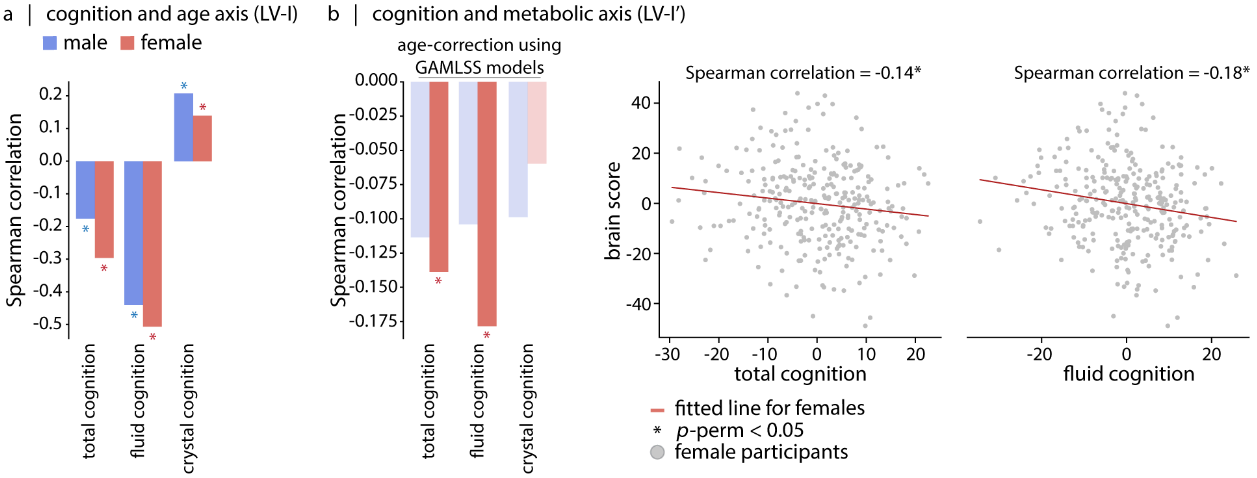

- Regarding the validation in UKB: maybe it would be feasible to use the HCP-A decomposition (potentially without indicators missing in UKB) and apply it to the UKB data to see agreement instead of performing completely new analysis? I am wondering whether you could even more clearly say that the same profiles are found if you followed this approach.'

In response to this comment, we performed an additional analysis to test whether the latent brain–biomarker profiles identified in one dataset (HCP-A) generalize to an independent cohort (UK Biobank). Specifically, we trained sex-stratified PLS models in HCP-A using only those brain features and biomarkers that are shared between the two datasets, and then projected UK Biobank data directly into the HCP-A-derived latent space. This approach allows us to assess whether the same multivariate covariance structure is preserved across cohorts. The full description of the validation analysis has now been incorporated into the *Results* section under a newly added subsection entitled "*Validation and sensitivity analysis*," as detailed below.

"**Validation and sensitivity analysis**

We next ask whether a PLS model trained on one dataset (HCP-A) generalizes to explain brain–biomarker relationships in an independent cohort (UK Biobank). To answer this question, we first indicate the brain features and biomarkers shared across the two datasets. Shared brain features include white matter FA, white matter MD, WMH, blood perfusion, ATT, and cortical thickness. Shared biomarkers include age, BMI, ALK, AST, ALT, urea, calcium, creatinine, bilirubin, glucose, HbA1c, HDL cholesterol, LDL cholesterol, triglycerides, total cholesterol, testosterone, total protein, and vitamin D. For each individual, brain features are averaged across all available parcels, yielding six global brain measures per individual. Using these shared features (6 brain measures and 18 biomarkers), we construct sex-stratified PLS models exclusively in the HCP-A dataset. Again, in both males and females, the first latent variable (LV-I) predominantly captures age-related covariations and the second latent variable (LV-II) captures metabolic-related covariations (Fig. S22).

In males, LV-I indicates that greater age is associated with lower cortical thickness, FA, and blood perfusion, greater MD and WMH load, and longer ATT (brain and biomarker score correlation $r = 0.68$, $p_{perm} = 9.99 \times 10^{-4}$, $N_{perm} = 1,000$). LV-II indicates that worse metabolic function is associated with lower blood perfusion and shorter ATT (brain and biomarker score correlation $r = 0.43$, $p_{perm} = 9.99 \times 10^{-4}$, $N_{perm} = 1,000$). Next, we project the UK Biobank brain and biomarker data onto the HCP-A-derived PLS weight vectors. We find a significant correlation between projected brain and biomarker scores in the UK Biobank cohort. We assess the significance of the correlation value by permuting the order of UK Biobank subjects on the biomarker side (

$N_{perm} = 10,000$), while keeping brain data fixed, and constructing a null distribution of correlations from the projected data. For LV-I, the Pearson correlation between projected brain and biomarker scores in UK Biobank is equal to 0.41 ($p_{perm} = 9.999 \times 10^{-5}$). For LV-II, the Pearson correlation between projected brain and biomarker scores in UK Biobank is equal to 0.12 ($p_{perm} = 9.999 \times 10^{-5}$).

In females, LV-I indicates that greater age is associated with lower cortical thickness, FA, and blood perfusion, greater MD, and WMH load (brain and biomarker score correlation $r = 0.68$, $p_{perm} = 9.99 \times 10^{-4}$, $N_{perm} = 1,000$). LV-II indicates that worse metabolic function is associated with lower blood perfusion, lower MD, and shorter ATT (brain and biomarker score correlation $r = 0.36$, $p_{perm} = 3.996 \times 10^{-3}$, $N_{perm} = 1,000$). Next, we project the UK Biobank data into the HCP-A-derived PLS space. For LV-I, the Pearson correlation between projected brain and biomarker scores in UK Biobank is equal to 0.46 ($p_{perm} = 9.999 \times 10^{-5}$). For LV-II, the Pearson correlation between projected brain and biomarker scores in UK Biobank is equal to 0.13 ($p_{perm} = 9.999 \times 10^{-5}$). Together, these results demonstrate that the brain–biomarker covariance structure identified in HCP-A—including both the age-dominated and metabolic latent variables—generalizes to an independent dataset."

In addition, we have added a new supplementary figure (Figure S22) to the manuscript. The figure and its corresponding caption are provided below.

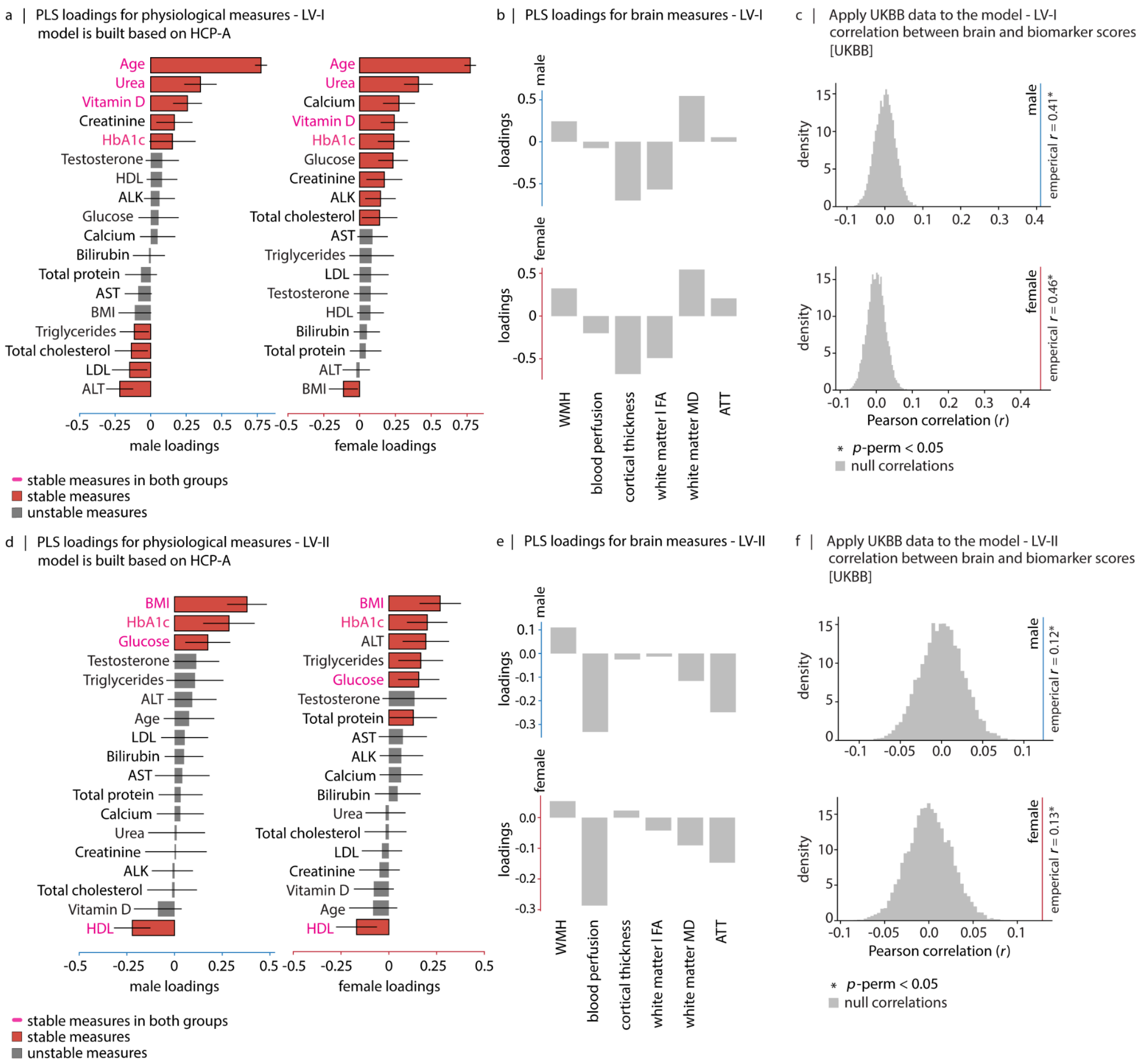

"**Figure S22. Generalization of HCP-A–derived PLS latent variables to the UK Biobank cohort |** We identify brain features and biomarkers shared across the HCP-A and UK Biobank datasets. We use these features to construct sex-stratified PLS models based on the HCP-A data. (a) Biomarker loadings of the first latent variable. Bootstrap resampling is used to estimate the stability of each individual biomarker's contribution to the overall multivariate pattern. Stable biomarkers are shown in red. (b) Brain loadings of the first latent variable. Each bar represents a global brain feature. (c) Using the weights of the PLS models, we mapped the UK Biobank data into the HCP-A–derived latent space. For the first latent variable, significant correlations between projected brain and biomarker scores are observed for males (top; $r = 0.41$, $p_{perm} = 9.999 \times 10^{-5}$) and females (bottom; $r = 0.46$, $p_{perm} = 9.999 \times 10^{-5}$). (d) Biomarker loadings of the second latent variable. (e) Brain loadings of the second latent variable. (f) Using the weights of the PLS models, we mapped the UK Biobank data into the

HCP-A–derived latent space. For the second latent variable, significant correlations between projected brain and biomarker scores are observed for males (top; $r = 0.12, p_{perm} = 9.999 \times 10^{-5}$) and females (bottom; $r = 0.13, p_{perm} = 9.999 \times 10^{-5}$)."

- It is not clear to me what Figures S6 etc. are supposed to show, could you make it more visible (that highest loadings are for perfusion, I guess). Could you provide a comparison between sexes for the metabolic factors too, to convince readers the components are really similar between sexes?

The purpose of Figure S9 (previous Figure S6) is to show the relative contribution of different brain features to each latent variable (LV). In the main text of the manuscript, we argue that brain blood perfusion is the brain feature with the strongest contribution to LV-II (the metabolic-related LV). Figure S9 provides a statistical confirmation of this observation by explicitly comparing the loadings across brain features (see text below).

> "On the brain side, this biomarker pattern is prominently associated with lower blood perfusion. Additional associations include lower FC strength and MD; the inverse association between BMI and MD potentially reflects the presence of gliosis [citation]. Other cortical features make relatively smaller contributions (Fig. 3b and S9)."

For completeness, we also report the same analysis for LV-I (the age-related LV) in Figure S5, although it is not the primary focus of the manuscript.

Regarding sex comparisons, males and females were analyzed using two separate PLS models. As a result, the raw loading magnitudes cannot be directly compared across sexes. Instead, we compare the cross-sex spatial pattern of brain loadings, which provides an interpretable basis for comparison across independent models. Accordingly, in Figures S8 and S11, we quantify cross-sex similarity by computing the spatial correlation between the brain loading maps. These analyses show that the brain loadings of latent components (LV-I and LV-II) exhibit high spatial similarity between males and females. Figures S8 and S11 are included in the response letter.

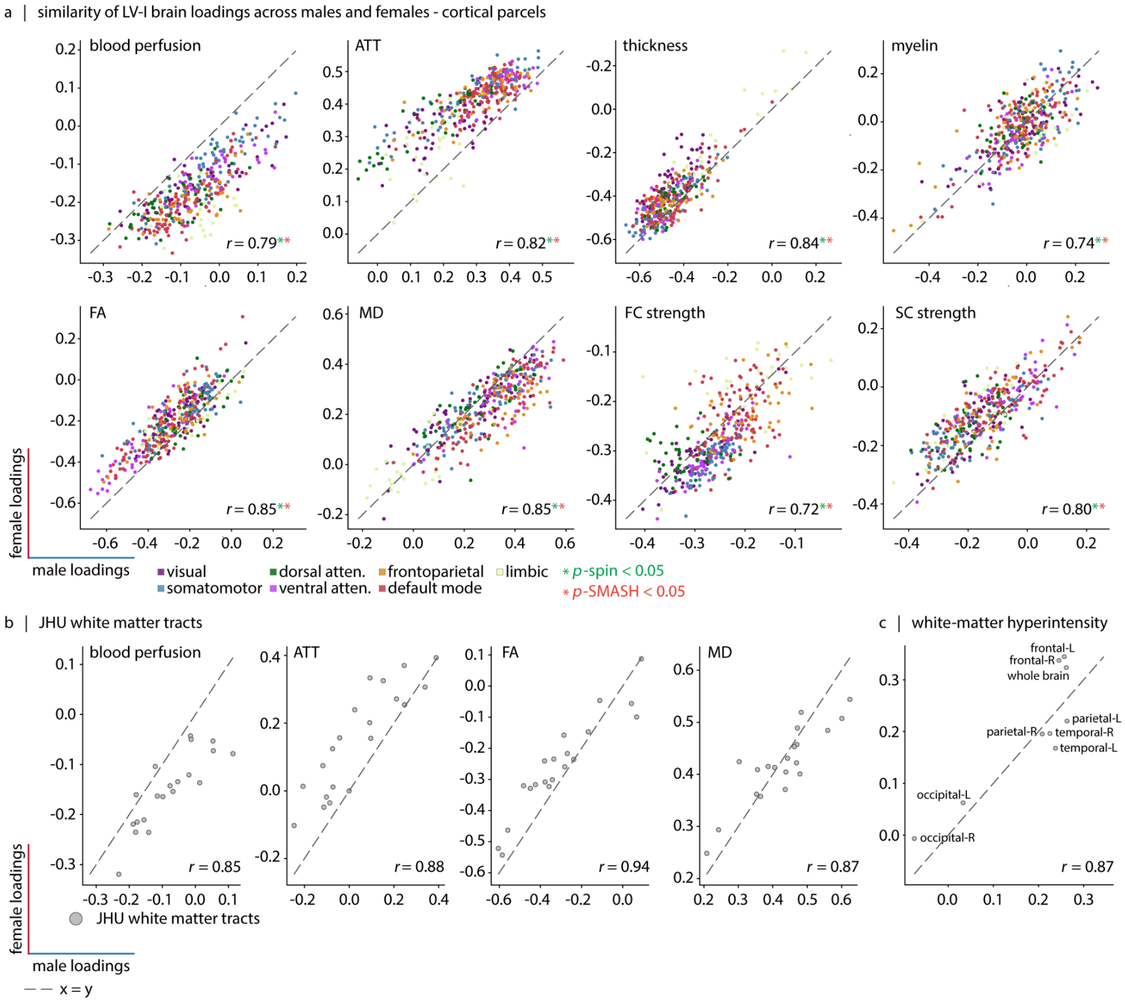

"**Figure S8. Similarity of first latent variable brain loadings between males and females |** (a) Cortical brain loadings comparison. In the scatter plots, each dot represents a cortical brain region defined by the Schaefer-400 parcellation; the dots are color-coded based on the Yeo-7 functional resting-state networks. The x-axis shows male brain loadings and the y-axis shows female brain loadings. Green asterisks indicate significant associations where the significance of empirical correlation is assessed using spin tests ($p_{spin} < 0.05$) and orange asterisks indicate significant associations where the significance of empirical correlation is assessed using variogram-estimating null models ($p_{SMASH} < 0.05$). (b) White matter tract loadings comparison. The scatter plots show the similarity of brain loadings between males (x-axis) and females (y-axis) for white matter tract measures, including blood perfusion, arterial transit time (ATT), fractional anisotropy (FA), and mean diffusivity (MD). (c) White matter hyperintensity loadings comparison. The scatter plot shows the similarity of brain loadings between males (x-axis) and females (y-axis) for the white matter hyperintensity measure."

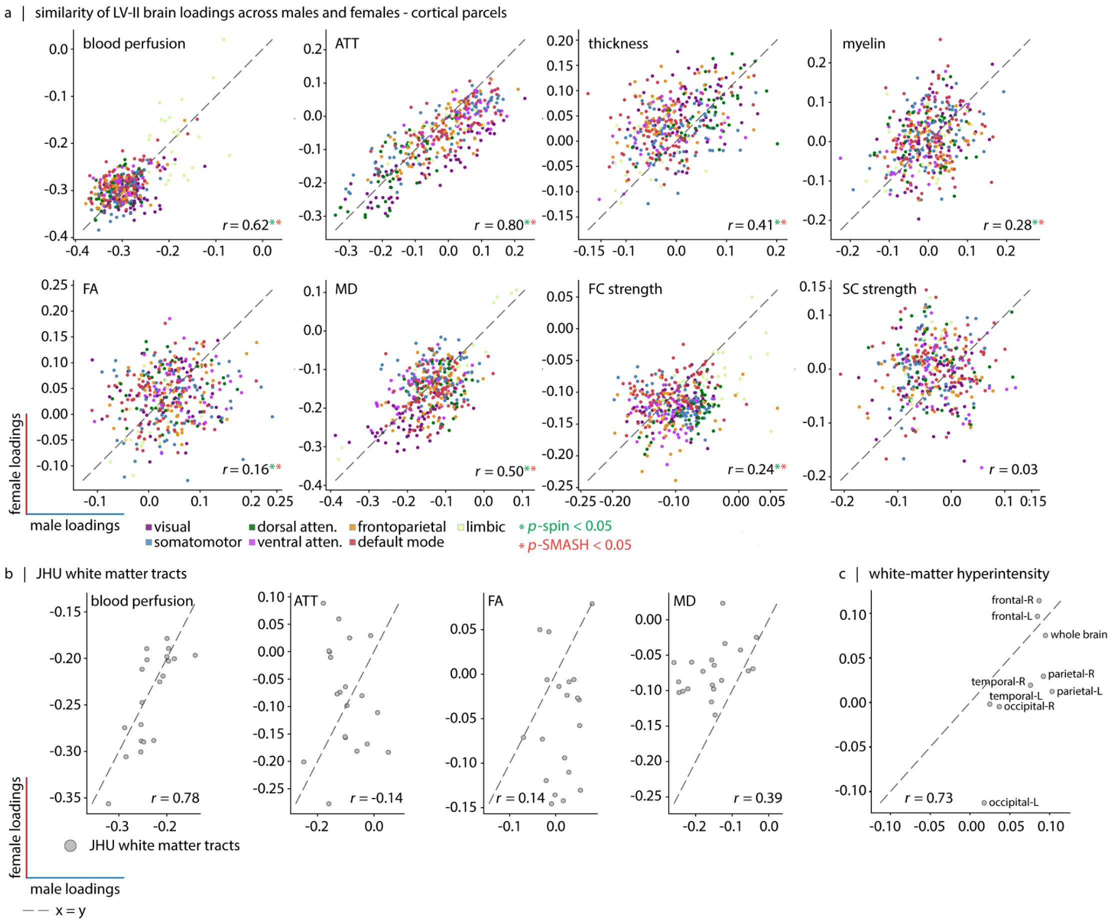

“**Figure S11. Similarity of second latent variable brain loadings between males and females |** (a) Cortical brain loadings comparison. In the scatter plots, each dot represents a cortical brain region defined by the Schaefer-400 parcellation; the dots are color-coded based on the Yeo-7 functional resting-state networks. The x-axis shows male brain loadings and the y-axis shows female brain loadings. Green asterisks indicate significant associations where the significance of empirical correlation is assessed using spin tests ($p_{spin} < 0.05$) and orange asterisks indicate significant associations where the significance of empirical correlation is assessed using variogram-estimating null models ($p_{SMASH} < 0.05$). (b) White matter tract loadings comparison. The scatter plots show the similarity of brain loadings between males (x-axis) and females (y-axis) for white matter tract measures, including blood perfusion, arterial transit time (ATT), fractional anisotropy (FA), and mean diffusivity (MD). (c) White matter hyperintensity loadings comparison. The scatter plot shows the similarity of brain loadings between males (x-axis) and females (y-axis) for the white matter hyperintensity measure.”

Discussion:
"Some individuals demonstrate resilience to expected metabolic brain correlates despite peripheral risk factors, while some show heightened vulnerability (discrepancy between brain and peripheral biomarker profiles; see Fig. 3c, 5c, S14c and S16c)." → explain in more detail, and highlight two examples in the figure.

Not all individuals exposed to metabolic dysfunction experience equivalent brain outcomes. Although metabolic brain scores and peripheral biomarker scores are positively associated at the group level, their correlation is below unity, indicating the potential inter-individual variability in how the brain may be affected by a given peripheral metabolic burden. Some individuals show elevated metabolic dysfunction on the biomarker side but relatively preserved or less affected brain signatures. These individuals fall above the main brain–biomarker regression line in Figure 3c and 5c, and represent a resilient phenotype, in which the brain appears comparatively protected relative to metabolic risk. Conversely, some individuals show pronounced adverse brain metabolic signatures despite only moderate peripheral risk (those below the regression line). These cases reflect a vulnerable phenotype, in which the brain is particularly sensitive to metabolic perturbations.

To clarify this point, we have now added a new supplementary figure (Figure S27) to further illustrate these patterns across datasets and latent variables, and we now refer to this point explicitly in the *Discussion* section:

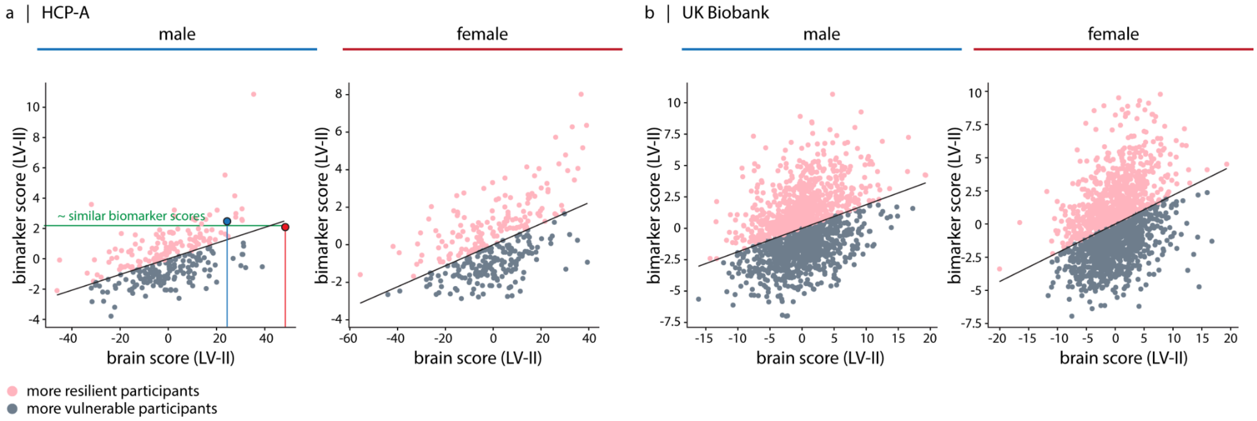

"**Figure S27. Individual variability in brain outcomes in metabolic syndrome |** Not all individuals exposed to metabolic dysfunction experience equivalent brain outcomes. (a) Brain and biomarker scores for LV-II in HCP-A, (b) and in UK Biobank. Some individuals show elevated metabolic dysfunction on the biomarker side but relatively preserved or less affected brain signatures. These individuals fall below the main brain–biomarker regression line (pink dots), and represent a resilient phenotype, in which the brain appears comparatively protected relative to metabolic risk. Conversely, some individuals show adverse brain metabolic signatures despite only moderate peripheral risk (gray dots). These cases reflect a vulnerable phenotype, in which the brain is particularly sensitive to metabolic perturbations. On the leftmost panel, we highlight two male

> participants from the HCP-A cohort with comparable biomarker scores but divergent brain scores. The participant shown in blue has a smaller brain score and the one in red has a higher brain score, meaning that despite similar peripheral metabolic profiles, the participant shown in red has greater brain vulnerability."

We further provide some explanation for why the discrepancy between brain and biomarker scores may exist. We have now expanded this point in the *Discussion* as follows:

> "This study provides a framework for studying the mechanisms of neuroprotection and vulnerability in metabolic dysfunction. Not all individuals exposed to metabolic dysfunction experience equivalent brain outcomes. Some individuals demonstrate resilience to expected metabolic brain correlates despite peripheral risk factors, while some show heightened vulnerability (discrepancy between brain and peripheral biomarker profiles; see Fig. S27). While part of this discrepancy may originate from measurement errors, it is also conceivable that genetic predisposition and environmental factors modulate the heterogeneity of interactions between brain and peripheral features [1]. Our framework quantifies this discrepancy and provides an opportunity for targeted genetic investigations to identify genetic variants that may confer resilience to brain alterations caused by metabolic risks and to test the contribution of behavioral and lifestyle factors—such as smoking, physical activity, socioeconomic status, and pollution exposure—on shaping this discrepancy. One plausible example of how genetics can alter the association between brain and bodily metabolic signature is detailed here: vascular dysfunction and arterial stiffness partially mediate the link between systemic metabolic conditions and brain blood flow, function and structure [2], meanwhile, specific genes are known to contribute to individuals' propensity to develop arterial stiffening [3, 4], which may, in return, amplify the downstream effects of metabolic abnormalities on the brains of these vulnerable individuals."

Added references:

[1] Assis, S. G., Tavares, P. H., Oliveira, N., Serpeloni, F., & Avanci, J. Q. (2025). Epigenetics, resilience, protective factors and factors promoting positive outcomes: A scoping review. *International Journal of Developmental Neuroscience*, *85*(6), e70042.

[2] Pasha, E. P., Birdsill, A. C., Oleson, S., Haley, A. P., & Tanaka, H. (2018). Impacts of metabolic syndrome scores on cerebrovascular conductance are mediated by arterial stiffening. *American Journal of Hypertension*, *31*(1), 72-79.

[3] Lacolley, P., Challande, P., Osborne-Pellegrin, M., & Regnault, V. (2009). Genetics and pathophysiology of arterial stiffness. *Cardiovascular research*, *81*(4), 637-648.

[4] Cecelja, M., & Chowienczyk, P. (2012). Role of arterial stiffness in cardiovascular disease. *JRSM cardiovascular disease*, *1*(4), 1-10.

I don't fully understand why you included this (interesting) paragraph "The present study is part of a wider effort to understand links between the brain and the body. These bidirectional interactions are increasingly explored in the context of the gut-brain axis [142] and the proteomics-brain axis [93, 155]. The gut-brain axis consists of two communication mechanisms: one mediated based on chemicals including, neurotransmitters, neuropeptides, hormones, and gut microbiome [159], and the other mediated by the physical connections via the vagus nerve. This axis is increasingly recognized as important for understanding the spread of proteinopathies in neurodegenerative diseases [181, 229]. Furthermore, there is evidence that this axis of interaction plays a role in obesity and metabolic health via serotonergic signaling [92, 98]. Concurrently, proteomics can also help to monitor brain health, and specifically to define protein signatures associated with obesity, type 2 diabetes, and neurodegenerative diseases. The identified protein profiles enable disease vulnerability predictions and identification of potential target molecules for effective intervention plans [93, 119, 155, 176, 203, 236]." - maybe shorten if you want to make a point that other "-brain axis" exist?

This paragraph was included to emphasize that brain–body interactions are mediated through multiple biological axes rather than a single pathway studied in our case which is the interaction between a confined set of brain features and plasma markers. More broadly, the present study focuses on a statistical model, and we thought it might be informative for readers to get a sense of the mechanistic links between the brain and periphery that underlie these statistical relationships. We also want to highlight that studying other axes can be informative for metabolic research. We now removed the statements related to the role of these pathways to neurodegenerative diseases, and retained only examples directly relevant to metabolism. The updated paragraph now reads as follows:

> "The present study is part of a wider effort to understand links between the brain and the body. These bidirectional interactions are increasingly explored in the context of the gut-brain axis [citation] and the proteomics-brain axis [citation]. The gut-brain axis consists of two communication mechanisms: one mediated by chemicals including neurotransmitters, neuropeptides, hormones, and gut microbiome [citation], and the other mediated by the physical connections via the vagus nerve. There is evidence that this axis of interaction plays a role in obesity and metabolic health via serotonergic signaling [citation]. Concurrently, proteomics can also help to monitor brain health by defining protein signatures associated with obesity and type 2 diabetes. The identified protein profiles enable the identification of potential target molecules for effective intervention plans [citation]."

- I don't agree with the term "powerful" here: "the present report shows that a composite metabolic brain-body profile may be a powerful way to identify links between

physiological measurements and cognitive outcome." as the associations are fairly weak (especially in the UKBB) and you don't show that the physiological scores derived from PLS are more associated with cognition than e.g. BMI alone.

We have revised the wording to adopt a more suitable tone, avoiding overstatement of effect size or predictive strength. Accordingly, the sentence in the *Discussion* now reads:

> "The present report shows that a composite metabolic brain–body profile can identify links between physiological measurements and cognitive outcomes."

- In the interpretation of the sex differences, you mention "Our results point to possible sex differences in metabolic-cognitive associations." I miss a statement about the similarity of the especially metabolic component as such (which are surprisingly similar, I find). It does not seem that body fat % or any hormonal measure (e.g. estradiol) has a significantly different weight in males vs females. Is this because differences would be rather expected within gender (i.e. women with higher levels of estradiol are protected against metabolic risk factors)? I would recommend to extend the discussion on this point.

When relating brain features to biomarkers within the metabolic dysfunction latent variable, biomarker loadings show substantial overlap between sexes. Indeed, on the brain loadings side, we observe some divergence in brain loadings across sexes. For example, blood-flow–related imaging measures (blood perfusion $r = 0.62$; ATT $r = 0.80$) exhibit highly similar spatial loading patterns in males and females, we find that structural measures show less complete overlap (thickness $r = 0.41$; cortical MD $r = 0.50$), indicating sex-dependent differences in how comparable metabolic dysfunction maps onto brain anatomy. These differences in how peripheral metabolic factors affect the brain may explain why we see more cognitive impairments linked to metabolism in females. It is possible that sex-related biological factors—such as hormonal milieu—modulate how a shared metabolic burden impacts the brain, leading to differential cognitive outcomes.

> "Our results point to possible sex differences in metabolic-cognitive associations. In the HCP-A dataset, greater metabolic deviation from health is associated with lower fluid cognition scores in females, whereas no significant negative associations survive multiple comparison correction in males. Consistent with prior report [1-5], we also find that metabolic burden affects brain structure and function differently in males and females (e.g., as reflected by partial divergence in brain loadings; see Fig. S11 and S13). Greater female cognitive susceptibility to metabolic dysregulation is also reported in multiple independent studies [citations]. The potentially greater cognitive effects observed in females may account for the higher Alzheimer's disease prevalence in females compared to males [citations]. Differential cognitive risk and resilience may be driven by sex-related factors ranging from physiological differences to acquired socioeconomic disparities [citations]. For example, sex hormones may influence

blood–brain barrier integrity, endothelial function, and vascular regulation [6-9] —systems that are also sensitive to metabolic dysfunction—thereby shaping sex-specific vulnerability to metabolic brain injury. Metabolic health and sex differences should be integrated in future aging and neurodegenerative disease studies to understand cognitive resilience disparities across individuals in greater detail."

The following references are now added to the manuscript:

[1] Horstmann, A., Busse, F. P., Mathar, D., Mueller, K., Lepsien, J., Schloegl, H., ... & Pleger, B. (2011). Obesity-related differences between women and men in brain structure and goal-directed behavior. *Frontiers in human neuroscience*, *5*, 58.

[2] Mueller, K., Anwander, A., Möller, H. E., Horstmann, A., Lepsien, J., Busse, F., ... & Pleger, B. (2011). Sex-dependent influences of obesity on cerebral white matter investigated by diffusion-tensor imaging. *PloS one*, *6*(4), e18544.

[3] Malpetti, M., Sala, A., Vanoli, E. G., Gianolli, L., Luzi, L., & Perani, D. (2018). Unfavourable gender effect of high body mass index on brain metabolism and connectivity. *Scientific Reports*, *8*(1), 12584.

[4] Dekkers, I. A., Jansen, P. R., & Lamb, H. J. (2019). Obesity, brain volume, and white matter microstructure at MRI: a cross-sectional UK biobank study. *Radiology*, *291*(3), 763-771.

[5] Hohl, R., de Morais, F. G. F., Taporoski, T. P., Negrão, A. B., Evans, S. L., De Oliveira, C. M., ... & Alvim, R. D. O. (2025). Sex-Specific Impact of Metabolic Syndrome on Brain Structures Vulnerable to Alzheimer's Disease: A Cross-Sectional Study in a Brazilian Cohort. *Brain sciences*, *15*(12), 1341.

[6] Collignon, A., Dion-Albert, L., Ménard, C., & Coelho-Santos, V. (2024). Sex, hormones and cerebrovascular function: from development to disorder. *Fluids and Barriers of the CNS*, *21*(1), 2.

[7] Cipolla, M. J., Godfrey, J. A., & Wiegman, M. J. (2009). The effect of ovariectomy and estrogen on penetrating brain arterioles and blood-brain barrier permeability. *Microcirculation*, *16*(8), 685-693.

[8] Krause, D. N., Duckles, S. P., & Pelligrino, D. A. (2006). Influence of sex steroid hormones on cerebrovascular function. *Journal of applied physiology*, *101*(4), 1252-1261.

[9] Miller, V. M., & Mulvagh, S. L. (2007). Sex steroids and endothelial function: translating basic science to clinical practice. *Trends in pharmacological sciences*, *28*(6), 263-270.

Methods:

How did you select the biomarkers used in the UKBB? (mention somewhere these were all biomarkers you used for HCP-A & available in UKBB if that's accurate)

A similar statement has now been added to the *Methods* section, "*UK Biobank: Biological samples and vital signs"* subsection:

> "The UK Biobank dataset included a comprehensive array of participant-specific blood and urine laboratory results in addition to obesity-related measures, blood pressure and age. The laboratory-based assessments included C-reactive protein, total protein, testosterone, glucose, HbA1c, cholesterol, HDL, LDL, triglycerides, urea, calcium, bilirubin, creatinine, vitamin D, AST, ALT, ALK, sodium urine, potassium urine, and systolic and diastolic blood pressures. Furthermore, participants' directory included data on biomarkers as age, BMI, body fat percentage, waist and hip circumference. These biomarkers were chosen to mirror those used in the HCP-A dataset, restricted to measures available in the UK Biobank, in order to maximize cross-dataset comparability."

"We also performed an additional PLS analysis in which the effect of age effect was regressed out from all brain imaging and physiological features." → remove one "effect", which age (initial or imaging assessment) was regressed?

We have removed the redundant use of the term *"effect"* and clarified which age variable was regressed out. The revised text now reads:

> "UK Biobank is a longitudinal study, laboratory results were acquired only at the initial assessment visit, while blood pressure and obesity-related measures (e.g., BMI, hip and waist circumference and body fat percentage) were acquired at both the initial assessment and the follow-up brain imaging visits. We included age at baseline and age at imaging visit in the original PLS model. We also performed an additional PLS analysis in which the effect of age (at imaging visit) was regressed out from all brain imaging and physiological features."

Reviewer #2

The paper by Farahani and co authors, titled " Separable contributions of aging and metabolism to brain-body health" and submitted to the journal PLOS Biology, presents a partial least square analyses to relate various physiological variables to various brain variables.

Age is a difficult variable to include in the analyses. The age regressed partial least square analyses should be used for the main results instead as a sensitive analyses. First, it is the dominant source of variance in the analyses. Second, it is conceptually different from any other measure used in the analyses. The other data on that side of the equation are physiological measurements. Age is not that. It is deterministic, continuously increasing during life, and absolute. You can only get older. But you can change all other measures (whether by lifestyle or drugs). Finally, given how much age impacts all aspect of life (from development to aging), it is likely a cause factor of changes in both brain and body. Finally, when age is removed, the 1st component looks like the 2nd component of the uncorrected model, again suggesting that the first component is just age.

Age indeed affects both biomarkers and brain measures at the same time and in a coordinated manner. The first latent variable is capturing the covariance between brain features and biomarkers that are systematically altered by aging. In the manuscript we have discussed how measures such as LDL, FSH, and HbA1c are known to change with age. Above and beyond this age-related pattern, metabolic health emerges as a separable axis of brain–body coupling. Presenting both components together allows us to distinguish the inevitable trajectory of biological aging from metabolic variation that is modifiable.

We agree with the reviewer that the first component of the age-regressed model closely resembles the second component of the full model. Rather than undermining our interpretation, we believe this consistency reinforces the robustness and separability of the two axes. In the interest of full transparency, we present both the uncorrected and age-regressed results side by side, so that readers interested specifically in metabolic effects on the brain independent of age can consult those results directly.

Some of the results are circular in the sense that most of the non brain variables used are metabolic measures. Thus I'm not surprised the largest component is a metabolic factor. Though in numerous places in the text, it is suggested that it is novel that a latent factor of metabolism was found through data driven methods.

We would like to clarify that the novel aspect of our findings lies not in the identification of a metabolic factor per se, but in its systematic and reproducible coupling with specific brain signatures, namely, the co-occurrence of low cerebral blood perfusion and high metabolic dysfunction. We have now expanded this point in the *Discussion* as follows:

> “One consistent and reproducible finding across this study is the identification of a metabolic axis linking peripheral physiological markers and the brain. Prior studies have shown that metabolic factors are associated with brain health; for example, increased BMI and abdominal fat have been linked to both functional [citation] and structural brain alterations [citations]; and increased HbA1c and adiposity-related insulin resistance is linked to cortical thickness changes. Extending beyond these previously documented associations, we show that cerebral blood perfusion is the primary brain feature that potentially declines as a function of elevated metabolic risk factors…”

The dominance of metabolic measures on the biomarker side does not guarantee that a coherent association with brain features will emerge. A data-driven multivariate analysis could yield diffuse, or non-reproducible brain associations. Instead, we observe robust, spatially structured brain patterns associated with the metabolic latent variable that are replicable across sexes, stable under cross-validation within cohorts, and reproducible across datasets. This indicates that specific combinations of peripheral metabolic measures consistently map onto specific brain signatures, which is not a foregone conclusion.

We agree with the reviewer that expanding the biomarker space—for example, to include metabolites, proteomics, or other molecular measures—can uncover additional dimensions of brain–body interaction. As datasets with both brain imaging and peripheral bodily measures become increasingly available, this represents an important direction for future work. To reflect this point more explicitly, we have now added the following paragraph to the *Discussion* section.

> “Last, many of the biomarkers included in this study are directly linked to the metabolic status of the body and are highly interconnected (e.g., HDL, LDL, BMI, total cholesterol). Indeed, the choice of biomarkers shapes the reported axes of brain–body covariance in this report and contributes to the prominence of the metabolic latent variable. Future work incorporating more diverse and comprehensive plasma and urine measures may reveal latent brain–body dimensions that extend beyond the metabolic axis.”

Was age regressed from the cognitive variables?

Yes. When the goal was to assess whether cognitive measures are associated with metabolic health, the linear and nonlinear effects of age were removed from the cognitive variables. Specifically, for analyses focusing on the metabolic axis, cognitive variables were corrected for age effects using GAMLSS before computing their association with brain scores. To avoid ambiguity, we have clarified this point in the caption of Figure 6.

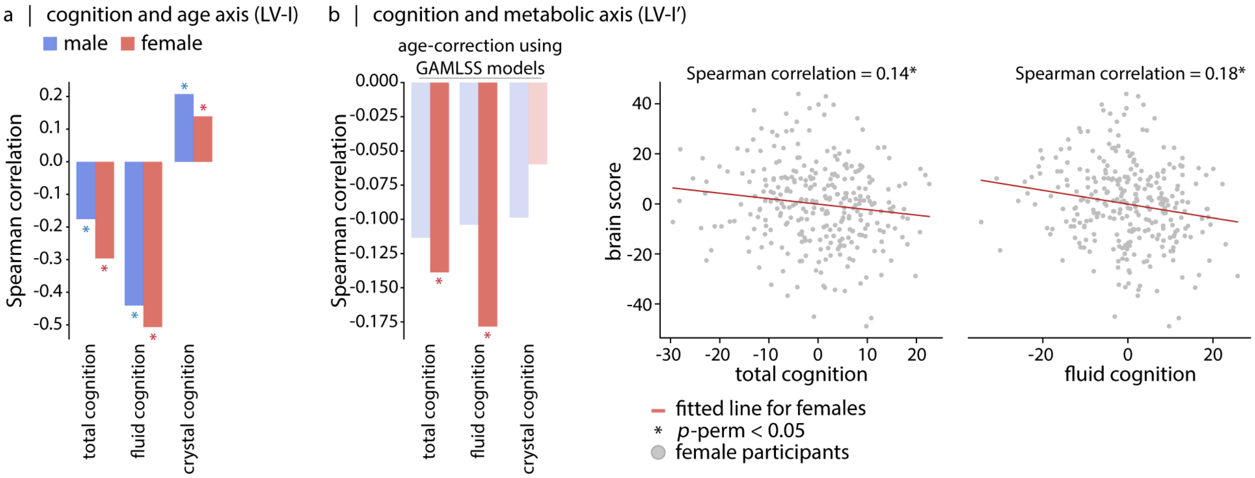

"Figure 6. Cognitive relevance of aging and metabolic axes | (a) Spearman's rank correlation between LV-I brain scores and total, fluid, and crystallized cognition composite scores (total cognition: $\rho =- 0.18$, $p_{perm} = 7.99 \times 10^{-3}$ (males), $\rho =- 0.30$, $p_{perm} = 1.49 \times 10^{-3}$ (females); fluid cognition: $\rho =- 0.44$, $p_{perm} = 2.99 \times 10^{-3}$ (males), $\rho =- 0.51$, $p_{perm} = 1.49 \times 10^{-3}$ (females); crystallized cognition: $\rho = 0.21$, $p_{perm} = 4.49 \times 10^{-3}$ (males), $\rho = 0.14$, $p_{perm} = 1.69 \times 10^{-2}$ (females)). (b) Spearman's rank correlation between LV-I′ brain score and cognitive measures, while GAMLSS models are used to correct for non-linear age effects across the cognitive variables. In this case, LV-I′ is capturing the metabolic axis (see Fig. 5). Total and fluid cognition composite scores are negatively associated with brain scores in females (total cognition: $\rho =- 0.14$, $p_{perm} = 2.55 \times 10^{-2}$ (females); fluid cognition: $\rho =- 0.18$, $p_{perm} = 1.20 \times 10^{-2}$(females)). Reported p-values are corrected for multiple comparisons using FDR within each biological sex-group."

The validation analyses is not "validation". It is replication. The authors repeated their analytical approach in a second dataset. Validation would be more along the lines how using latent factors found in the HPC dataset in the UK biobank dataset.

In response to this comment, we have performed the validation analysis as suggested and have shown that a PLS model trained on one dataset (HCP-A) generalizes to explain brain–biomarker relationships in an independent cohort (UK Biobank). The full description of the validation analysis has now been incorporated into the *Results* section under a newly added subsection entitled "*Validation and sensitivity analysis*," as detailed below.

“**Validation and sensitivity analysis**

We next ask whether a PLS model trained on one dataset (HCP-A) generalizes to explain brain–biomarker relationships in an independent cohort (UK Biobank). To answer this question, we first indicate the brain features and biomarkers shared across the two datasets. Shared brain features include white matter FA, white matter MD, WMH, blood perfusion, ATT, and cortical thickness. Shared biomarkers include age, BMI, ALK, AST, ALT, urea, calcium, creatinine, bilirubin, glucose, HbA1c, HDL cholesterol, LDL cholesterol, triglycerides, total cholesterol, testosterone, total protein, and vitamin D. For each individual, brain features are averaged across all available parcels, yielding six global brain measures per individual. Using these shared features (6 brain measures and 18 biomarkers), we construct sex-stratified PLS models exclusively in the HCP-A dataset. Again, in both males and females, the first latent variable (LV-I) predominantly captures age-related covariations and the second latent variable (LV-II) captures metabolic-related covariations (Fig. S22).

In males, LV-I indicates that greater age is associated with lower cortical thickness, FA, and blood perfusion, greater MD and WMH load, and longer ATT (brain and biomarker score correlation $r = 0.68$, $p_{perm} = 9.99 \times 10^{-4}$, $N_{perm} = 1,000$). LV-II indicates that worse metabolic function is associated with lower blood perfusion and shorter ATT (brain and biomarker score correlation $r = 0.43$, $p_{perm} = 9.99 \times 10^{-4}$, $N_{perm} = 1,000$). Next, we project the UK Biobank brain and biomarker data onto the HCP-A-derived PLS weight vectors. We find a significant correlation between projected brain and biomarker scores in the UK Biobank cohort. We assess the significance of the correlation value by permuting the order of UK Biobank subjects on the biomarker side ($N_{perm} = 10,000$), while keeping brain data fixed, and constructing a null distribution of correlations from the projected data. For LV-I, the Pearson correlation between projected brain and biomarker scores in UK Biobank is equal to 0.41 ($p_{perm} = 9.999 \times 10^{-5}$). For LV-II, the Pearson correlation between projected brain and biomarker scores in UK Biobank is equal to 0.12 ($p_{perm} = 9.999 \times 10^{-5}$).

In females, LV-I indicates that greater age is associated with lower cortical thickness, FA, and blood perfusion, greater MD, and WMH load (brain and biomarker score correlation $r = 0.68$, $p_{perm} = 9.99 \times 10^{-4}$, $N_{perm} = 1,000$). LV-II indicates that worse metabolic function is associated with lower blood perfusion, lower MD, and shorter ATT (brain and biomarker score correlation

$r = 0.36$, $p_{perm} = 3.996 \times 10^{-3}$, $N_{perm} = 1,000$). Next, we project the UK Biobank data into the HCP-A-derived PLS space. For LV-I, the Pearson correlation between projected brain and biomarker scores in UK Biobank is equal to 0.46 ($p_{perm} = 9.999 \times 10^{-5}$). For LV-II, the Pearson correlation between projected brain and biomarker scores in UK Biobank is equal to 0.13 ($p_{perm} = 9.999 \times 10^{-5}$). Together, these results demonstrate that the brain–biomarker covariance structure identified in HCP-A—including both the age-dominated and metabolic latent variables—generalizes to an independent dataset."

In addition, we have added a new supplementary figure (Figure S22) to the manuscript. The figure and its corresponding caption are provided below.

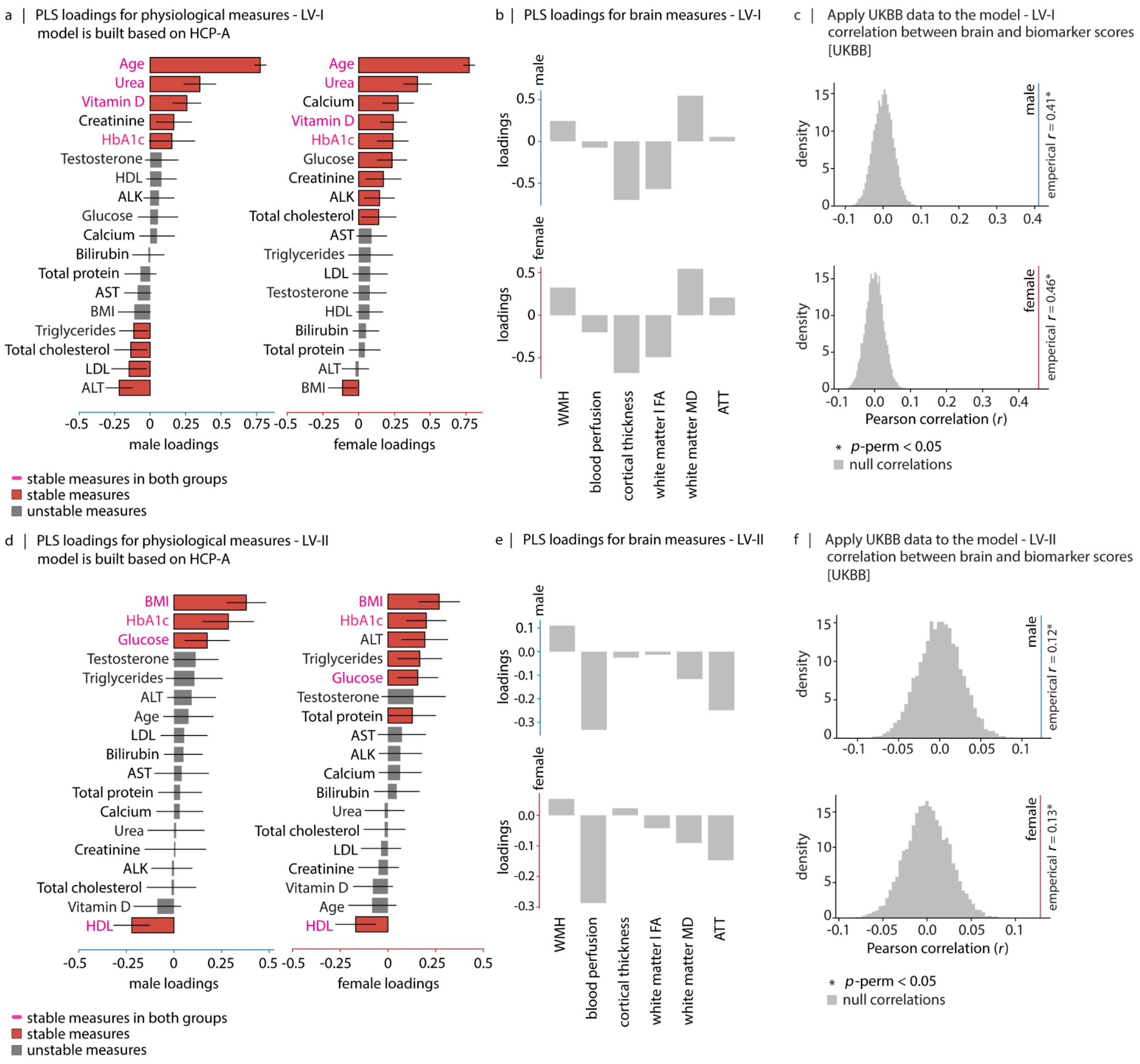

"**Figure S22. Generalization of HCP-A–derived PLS latent variables to the UK Biobank cohort |** We identify brain features and biomarkers shared across the HCP-A and UK Biobank datasets. We use these features to construct sex-stratified PLS models based on the HCP-A data. (a) Biomarker loadings of the first latent variable. Bootstrap resampling is used to estimate the stability of each individual biomarker's contribution to the overall multivariate pattern. Stable biomarkers are shown in red. (b) Brain loadings of the first latent variable. Each bar represents a global brain feature. (c) Using the weights of the PLS models, we mapped the UK Biobank data into the HCP-A–derived latent space. For the first latent variable, significant correlations between projected brain and biomarker scores are observed for males (top; $r = 0.41$, $p_{perm} = 9.999 \times 10^{-5}$) and females (bottom; $r = 0.46$, $p_{perm} = 9.999 \times 10^{-5}$). (d) Biomarker loadings

of the second latent variable. (e) Brain loadings of the second latent variable. (f) Using the weights of the PLS models, we mapped the UK Biobank data into the HCP-A–derived latent space. For the second latent variable, significant correlations between projected brain and biomarker scores are observed for males (top; $r = 0.12, p_{perm} = 9.999 \times 10^{-5}$) and females (bottom; $r = 0.13, p_{perm} = 9.999 \times 10^{-5}$)."

Additionally, we agree with the reviewer on the terminological distinction between validation and replication, and have revised the manuscript accordingly, renaming the section "*Validation: aging and metabolic axes in UK Biobank*" to "*Replication: aging and metabolic axes in UK Biobank*" to more accurately reflect the nature of analysis described in that subsection.

It would be helpful to state the number of features on each side of the plsr analyses in the main text. It is unclear at the start of the paper how many imaging features are being used.

We have now added this information both to the figure captions accompanying the presentation of PLS latent variables and to the *Methods* section.

In the captions for Figures 1, 3, and 5, we now state:

"The PLS model includes 28 features on the biomarker side and 3 289 features on the brain side."

In the captions for Figures S15, S17, and S19, we now state:

"The PLS model includes 33 features on the biomarker side and 490 features on the brain side."

In the *Methods* section, in the "*HCP-A: Brain imaging measurements*" subsection, we state:

"Altogether, the brain feature set included 3 289 values."

In the "*HCP-A: Biological samples and vital signs*" subsection, we state:

"In total, 28 biomarkers were included in the analysis for each participant."

In the "*UK Biobank: Brain imaging measurements*" subsection, we state:

"Altogether, the brain feature set included 490 values."

Finally, in the "*UK Biobank: Biological samples and vital signs*" subsection, we state:

"In total, 33 biomarkers were included in the analysis for each participant."

Along those lines, why was the HCP aging dataset used as the discovery dataset? The dimensions are very high compared to the sample size: 300 subjects (since males and females had separate models) compared to 28 biological features and 3000 brain features. This leads to unstable loading estimates (see helmer et al). It would be more logical to use the larger UKB as the main dataset with the HCP as the replication dataset. Helmer M, Warrington S, Mohammadi-Nejad AR, Ji JL, Howell A, Rosand B, Anticevic A, Sotiropoulos SN, Murray JD. On the stability of canonical correlation analysis and partial least squares with application to brain-behavior associations. Commun Biol. 2024 Feb 21;7(1):217. doi: 10.1038/s42003-024-05869-4. PMID: 38383808; PMCID: PMC11245620.

Regarding the choice of HCP-A as the primary discovery dataset, we would like to clarify that this decision was driven by several practical and methodological considerations listed below:

- HCP-A provides a more detailed multimodal brain imaging dataset than UK Biobank. In particular, the modalities most central to our findings—cerebral blood flow (CBF) and arterial transit time (ATT), derived from ASL imaging—are acquired at considerably higher resolution in HCP-A (2.5 mm whole-brain maps, later parcellated using the 400-region Schaefer atlas) compared to UK Biobank, where the equivalent measures are summarized only at the level of brain anatomical lobes (e.g., occipital, frontal). This difference in spatial resolution makes HCP-A the more appropriate dataset for characterizing the detailed brain signatures associated with each latent variable.
- As detailed in the Methods section and reported in Tables S1 and S2, the proportion of missing values is substantially higher in UK Biobank than in HCP-A. Any imputation strategy, regardless of the approach taken introduces assumptions that may obscure or distort the brain–biomarker covariance structure that our analysis aims to recover.
- As acknowledged in the *Limitations* section of the *Discussion*, In UK Biobank laboratory biomarkers were acquired at the baseline visit while neuroimaging data were collected at a follow-up session, with an interval that can span several years (see Figure S2). Although we mitigated this discrepancy by using GAMLSS models to regress out age effects from all features prior to PLS analysis, this temporal mismatch represents an additional source of noise.

In response to concerns regarding the stability of the latent variables, we note that several validation analyses were already included in the original manuscript, and we have conducted additional tests in this revision to further strengthen the robustness of the results.

In the first version of the manuscript, we showed that similar latent variables were recovered across biological sexes and replicated across two independent datasets (HCP-A and UK Biobank). Within each dataset, we also assessed the stability of covariance patterns found across brain and biomarker using cross-validation: the PLS model and latent variable space were estimated using 80% of the data and then validated on the remaining 20% held-out sample. Cross-validation was done in both biological sex groups and in both datasets.

In the revised manuscript, we performed three additional analyses to further evaluate the robustness and stability of the results:

- We reduced the dimensionality of the brain features by computing global measures for each brain imaging feature (e.g., overall cortical thickness instead of parcel-wise thickness across 400 regions). Despite this substantial reduction in spatial detail, we recovered highly similar latent variables, which again showed strong within-dataset cross-validation and consistency across sexes.
- We assessed generalizability across cohorts by first fitting the model in the HCP-A dataset and then showing that the model generalizes to the UK Biobank dataset.
- We applied sparse PLS and recovered the same dominant latent structure, indicating that the observed associations are not driven by weak or redundant features (see pages 40-43; response to Reviewer #3).

Together, these analyses provide converging evidence that the reported brain–biomarker relationships are stable, reproducible, and not dependent on specific modeling choices.

The details of the mentioned analysis are added to the manuscript. Specifically, a subsection called "*Validation and sensitivity analysis*" has been added to the *Results* section of the manuscript and is also included in the response letter:

"**Validation and sensitivity analysis**

We next ask whether a PLS model trained on one dataset (HCP-A) generalizes to explain brain–biomarker relationships in an independent cohort (UK Biobank). To answer this question, we first indicate the brain features and biomarkers shared across the two datasets. Shared brain features include white matter FA, white matter MD, WMH, blood perfusion, ATT, and cortical thickness. Shared biomarkers include age, BMI, ALK, AST, ALT, urea, calcium, creatinine, bilirubin, glucose, HbA1c, HDL cholesterol, LDL cholesterol, triglycerides, total cholesterol, testosterone, total protein, and vitamin D. For each individual, brain features are averaged across all available parcels, yielding six global brain measures per individual. Using these shared features (6 brain measures and 18 biomarkers), we construct sex-stratified PLS models exclusively in the HCP-A dataset. Again, in both males and females, the first latent variable (LV-I) predominantly captures age-related covariations and the second latent variable (LV-II) captures metabolic-related covariations (Fig. S22).

In males, LV-I indicates that greater age is associated with lower cortical thickness, FA, and blood perfusion, greater MD and WMH load, and longer ATT (brain and biomarker score correlation $r = 0.68$, $p_{perm} = 9.99 \times 10^{-4}$, $N_{perm} = 1,000$). LV-II indicates that worse metabolic function is associated with lower blood perfusion and shorter ATT (brain and biomarker score correlation

$r = 0.43$, $p_{perm} = 9.99 \times 10^{-4}$, $N_{perm} = 1,000$). Next, we project the UK Biobank brain and biomarker data onto the HCP-A-derived PLS weight vectors. We find a significant correlation between projected brain and biomarker scores in the UK Biobank cohort. We assess the significance of the correlation value by permuting the order of UK Biobank subjects on the biomarker side ($N_{perm} = 10,000$), while keeping brain data fixed, and constructing a null distribution of correlations from the projected data. For LV-I, the Pearson correlation between projected brain and biomarker scores in UK Biobank is equal to 0.41 ($p_{perm} = 9.999 \times 10^{-5}$). For LV-II, the Pearson correlation between projected brain and biomarker scores in UK Biobank is equal to 0.12 ($p_{perm} = 9.999 \times 10^{-5}$).

In females, LV-I indicates that greater age is associated with lower cortical thickness, FA, and blood perfusion, greater MD, and WMH load (brain and biomarker score correlation $r = 0.68$, $p_{perm} = 9.99 \times 10^{-4}$, $N_{perm} = 1,000$). LV-II indicates that worse metabolic function is associated with lower blood perfusion, lower MD, and shorter ATT (brain and biomarker score correlation $r = 0.36$, $p_{perm} = 3.99 \times 10^{-3}$, $N_{perm} = 1,000$). Next, we project the UK Biobank data into the HCP-A-derived PLS space. For LV-I, the Pearson correlation between projected brain and biomarker scores in UK Biobank is equal to 0.46 ($p_{perm} = 9.999 \times 10^{-5}$). For LV-II, the Pearson correlation between projected brain and biomarker scores in UK Biobank is equal to 0.13 ($p_{perm} = 9.999 \times 10^{-5}$). Together, these results demonstrate that the brain–biomarker covariance structure identified in HCP-A—including both the age-dominated and metabolic latent variables—generalizes to an independent dataset.

We also assess the potential instability of PLS results arising from the high dimensionality of the brain feature space relative to sample size (Helmer, *et al.*). To this end, each brain feature is averaged across all parcels, yielding a single global value per brain measure per participant and substantially reducing the PLS input dimensionality. Rebuilding the PLS models using this reduced feature set yields latent variables that are highly consistent with those obtained from the original analysis. Across both biological sexes and both datasets (HCP-A and UK Biobank), LV-I captures brain–body covariation related to aging and LV-II captures covariation related to metabolic dysfunction, confirming that the main findings are not artifacts of the high dimensionality of the PLS model input.

In the HCP-A dataset, LV-I indicates that greater age is associated with low cortical thickness, myelin, FA, functional and structural connectivity strength, and

blood perfusion, alongside high MD, WMH load, and ATT (score correlation: males $= 0.72$, females $= 0.68$; variance explained: 82.87% and 83.89%, respectively;$p_{perm} = 9.99 \times 10^{-4}$ for both) (Fig. S23). LV-II is characterized by lower HDL, higher BMI, MAP, insulin, and HbA1c, and worse liver enzyme profiles on the biomarker side, and is associated with low blood perfusion and MD on the brain side (score correlation: males $= 0.48$, females $= 0.50$; variance explained: 10.29% and 10.79%, respectively; $p_{perm} = 9.99 \times 10^{-4}$ for both) (Fig. S24).

In the UK Biobank dataset, LV-I indicates that greater age is associated with low cortical thickness, volume, FA, ICVF, and cortical area, alongside high MD, ISOVF, WMH load, and ATT (score correlation: males $= 0.60$, females $= 0.57$; variance explained: 84.16% and 87.21%, respectively; $p_{perm} = 9.99 \times 10^{-4}$ for both) (Fig. S25). LV-II is characterized by higher BMI, body fat percentage, waist and hip circumference, and worse liver enzyme profiles on the biomarker side, and is associated with low blood perfusion and MD on the brain side (score correlation: males $= 0.27$, females $= 0.24$; variance explained: 11.12% and 9.10%, respectively; $p_{perm} = 9.99 \times 10^{-4}$ for both) (Fig. S26).

The correspondence between biomarker and brain scores is cross-validated and is generalizable across participants in each biological sex group and both datasets ($p = 9.90 \times 10^{-3}$, $N = 100$)."

The added supplementary figures and their captions are included in the following:

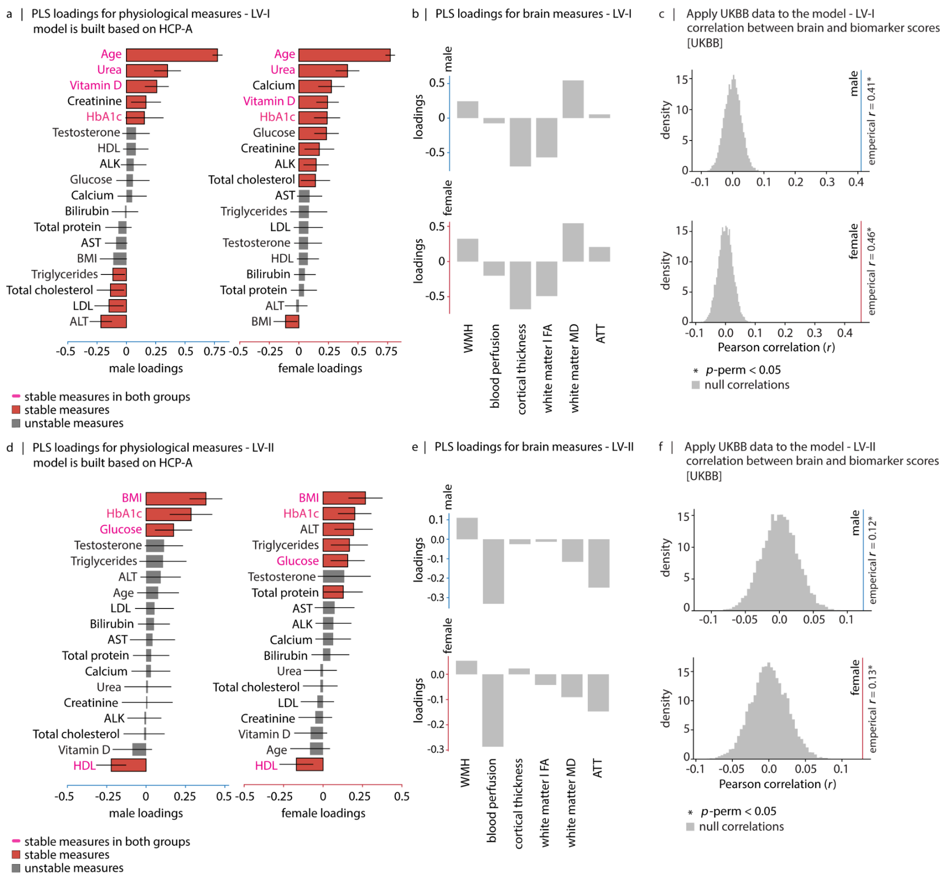

“**Figure S22. Generalization of HCP-A–derived PLS latent variables to the UK Biobank cohort |** We identify brain features and biomarkers shared across the HCP-A and UK Biobank datasets. We use these features to construct sex-stratified PLS models based on the HCP-A data. (a) Biomarker loadings of the first latent variable. Bootstrap resampling is used to estimate the stability of each individual biomarker's contribution to the overall multivariate pattern. Stable biomarkers are shown in red. (b) Brain loadings of the first latent variable. Each bar represents a global brain feature. (c) Using the weights of the PLS models, we mapped the UK Biobank data into the HCP-A–derived latent space. For the first latent variable, significant correlations between projected brain and biomarker scores are observed for males (top; $r = 0.41$, $p_{perm} = 9.999 \times 10^{-5}$) and females (bottom; $r = 0.46$, $p_{perm} = 9.999 \times 10^{-5}$). (d) Biomarker loadings of the second latent variable. (e) Brain loadings of the second latent variable. (f) Using the weights of the PLS models, we mapped the UK Biobank data into the HCP-A–derived latent space. For the second latent variable, significant correlations between projected brain and biomarker scores are observed for males (top; $r = 0.12$, $p_{perm} = 9.999 \times 10^{-5}$) and females (bottom; $r = 0.13$, $p_{perm} = 9.999 \times 10^{-5}$).”

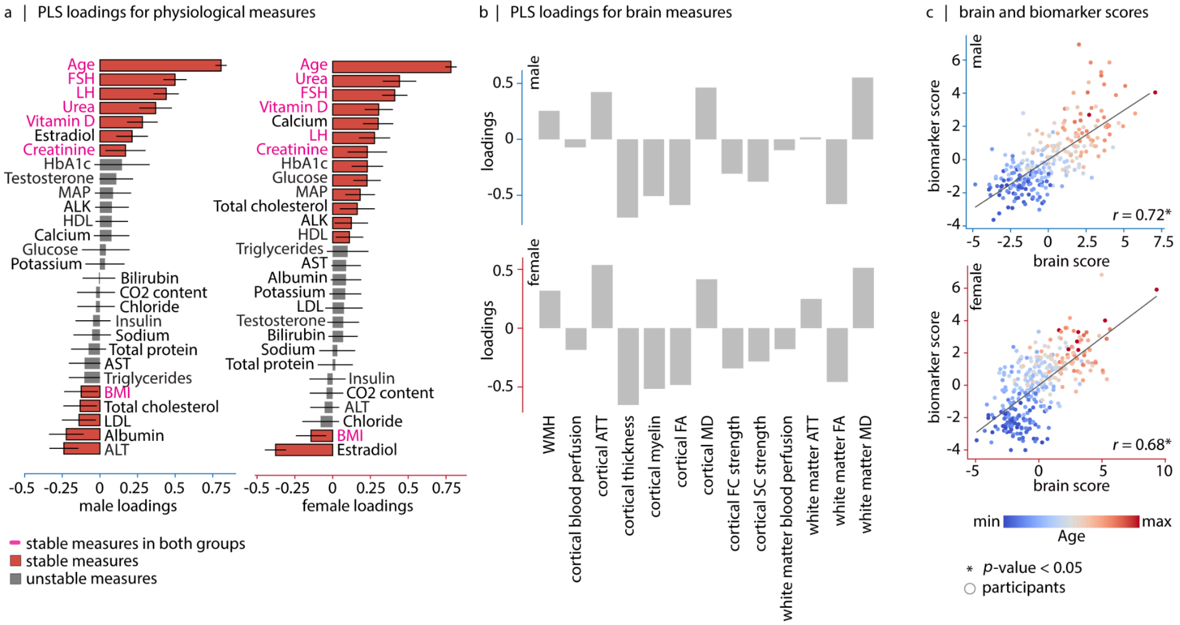

“**Figure S23. Mapping biomarkers to reduced brain features in the HCP-A dataset: first latent variable (LV-I) captures the aging axis |** Using PLS analysis, we identify a significant latent variable that accounts for 82.87% (males) and 83.89% (females) of the covariance between brain measurements and biomarkers. The PLS model includes 28 features on the biomarker side and 13 features on the brain side. (a) Biomarker loadings. Bootstrap resampling is used to estimate the stability of each individual biomarker's contribution to the overall multivariate pattern. Stable biomarkers for which the estimated 95% confidence intervals do not cross zero, are shown in red. (b) Brain loadings. Each bar represents a global brain measure. (c) Correlation between brain (x-axis) and biomarker scores (y-axis) for males (top; $r = 0.72$) and females (bottom; $r = 0.68$). Each dot represents an individual participant, colored by their age. Score correlation values passed cross-validation in both sex groups (for both: $p = 9.90 \times 10^{-3}$).”

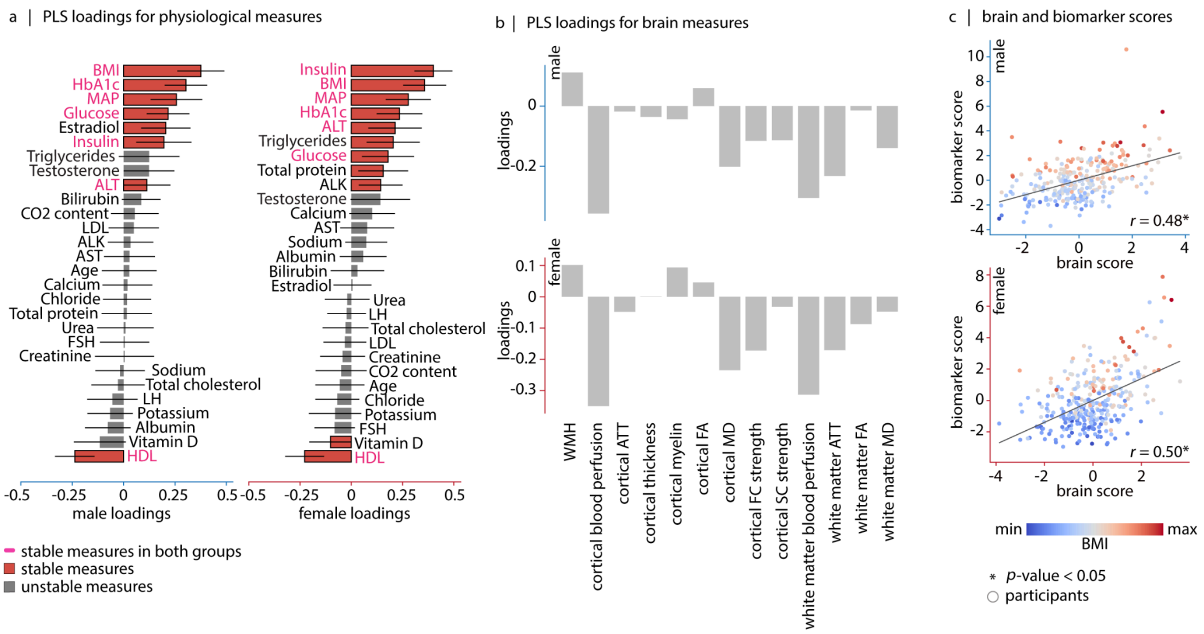

“Figure S24. Mapping biomarkers to reduced brain features in the HCP-A dataset: second latent variable (LV-II) captures the metabolic axis | Using PLS analysis, we identify a significant latent variable that accounts for 10.29% (males) and 10.79% (females) of the covariance between brain measurements and biomarkers. The PLS model includes 28 features on the biomarker side and 13 features on the brain side. (a) Biomarker loadings. Bootstrap resampling is used to estimate the stability of each individual biomarker's contribution to the overall multivariate pattern. Stable biomarkers for which the estimated 95% confidence intervals do not cross zero, are shown in red. (b) Brain loadings. Each bar represents a global brain measure. (c) Correlation between brain (x-axis) and biomarker scores (y-axis) for males (top; $r = 0.48$) and females (bottom; $r = 0.50$). Each dot represents an individual participant, colored by their BMI. Score correlation values passed cross-validation in both sex groups (for both: $p = 9.90 \times 10^{-3}$).”

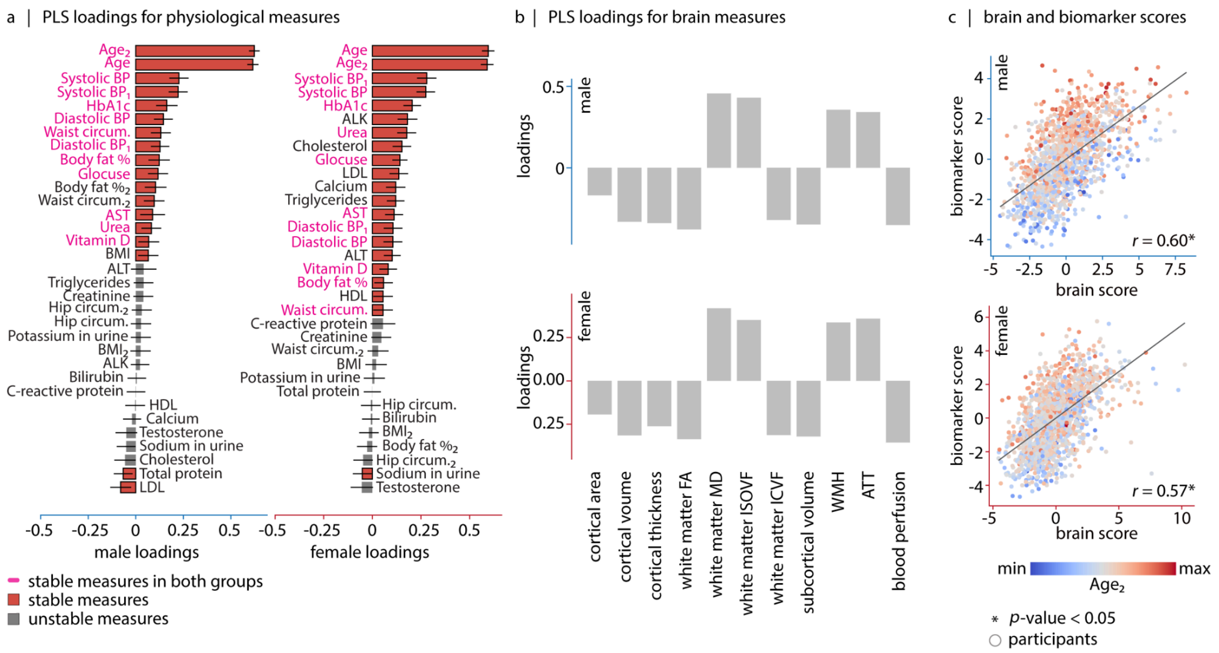

“**Figure S25. Mapping biomarkers to reduced brain features in the UK Biobank dataset: first latent variable (LV-I) captures the aging axis |** Using PLS analysis, we identify a significant latent variable that accounts for 84.16% (males) and 87.21% (females) of the covariance between brain measurements and biomarkers. The PLS model includes 33 features on the biomarker side and 11 features on the brain side. (a) Biomarker loadings. Bootstrap resampling is used to estimate the stability of each individual biomarker's contribution to the overall multivariate pattern. Stable biomarkers for which the estimated 95% confidence intervals do not cross zero, are shown in red. (b) Brain loadings. Each bar represents a global brain measure. (c) Correlation between brain (x-axis) and biomarker scores (y-axis) for males (top; $r = 0.60$) and females (bottom; $r = 0.57$). Each dot represents an individual participant, colored by their age. Score correlation values passed cross-validation in both sex groups (for both: $p = 9.90 \times 10^{-3}$).”

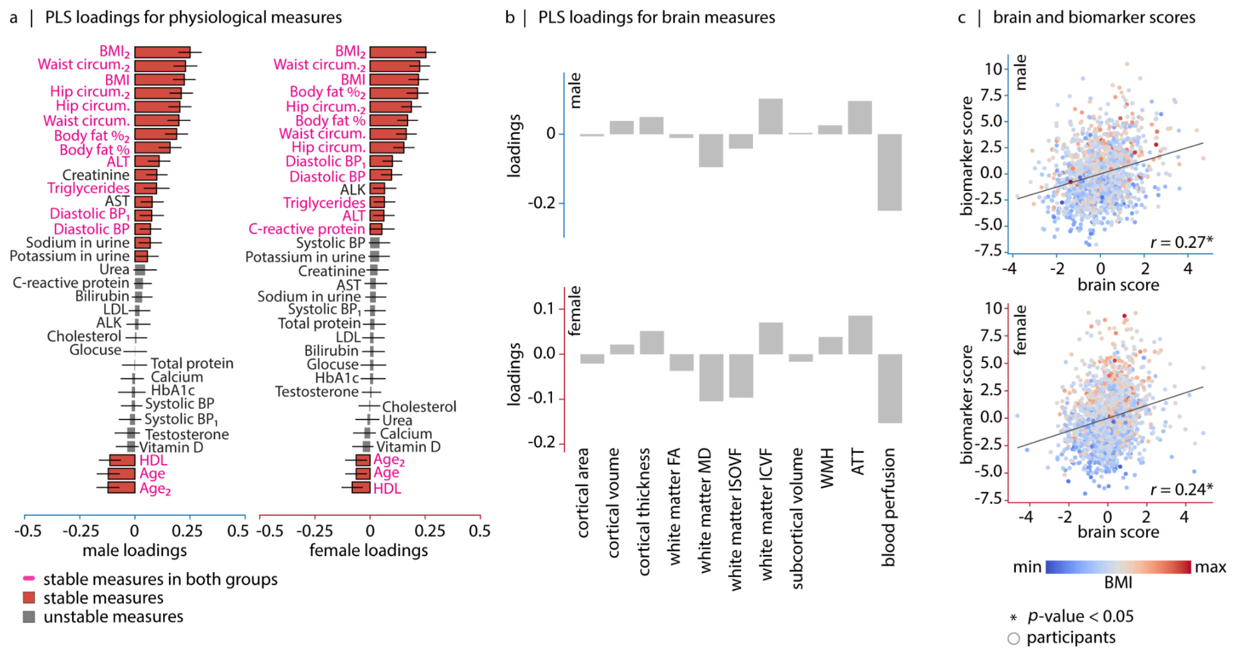

"**Figure S26. Mapping biomarkers to reduced brain features in the UK Biobank dataset: second latent variable (LV-II) captures the metabolic axis |** Using PLS analysis, we identify a significant latent variable that accounts for 11.12% (males) and 9.10% (females) of the covariance between brain measurements and biomarkers. The PLS model includes 33 features on the biomarker side and 11 features on the brain side. (a) Biomarker loadings. Bootstrap resampling is used to estimate the stability of each individual biomarker's contribution to the overall multivariate pattern. Stable biomarkers for which the estimated 95% confidence intervals do not cross zero, are shown in red. (b) Brain loadings. Each bar represents a global brain measure. (c) Correlation between brain (x-axis) and biomarker scores (y-axis) for males (top; $r = 0.27$) and females (bottom; $r = 0.24$). Each dot represents an individual participant, colored by their BMI. Score correlation values passed cross-validation in both sex groups (for both: $p = 9.90 \times 10^{-3}$)."

The following reference is also added to the manuscript:

"Helmer, M., Warrington, S., Mohammadi-Nejad, A. R., Ji, J. L., Howell, A., Rosand, B., ... & Murray, J. D. (2024). On the stability of canonical correlation analysis and partial least squares with application to brain-behavior associations. *Communications biology*, *7*(1), 217."

The spin test has numerous statistical issues centered around exchangeability violation. Something like Brain Smash has much better statistical properties and should be used.

Burt JB, Helmer M, Shinn M, Anticevic A, Murray JD. Generative modeling of brain maps with spatial autocorrelation. Neuroimage. 2020 Oct 15;220:117038. doi: 10.1016/j.neuroimage.2020.117038. Epub 2020 Jun 22. PMID: 32585343.

We have extended our reported statistics, and have further reported the p-values derived using the method introduced by Burt *et al.* (2020), which generates variogram-estimating null models. The corresponding p-values, along with a detailed description of the methodology, have now been added to the manuscript.

Specifically, we have updated the captions of Figure 2 and 4 to clarify that the significance of spatial similarity was reassessed using variogram-estimating null models. We also provide references to newly added supplementary figures presenting the BrainSMASH results.

In Figure caption 2, we have added the following statement:

> “In Fig. S7a, we reassess the significance of spatial similarity of the maps using the variogram-estimating null models.”

And in Figure caption 4, we have added the following statement:

> “In Fig. S7b, we reassess the significance of spatial similarity of the maps using the variogram-estimating null models.”

The new supplementary figure (Figure S7) and the corresponding figure caption are provided below:

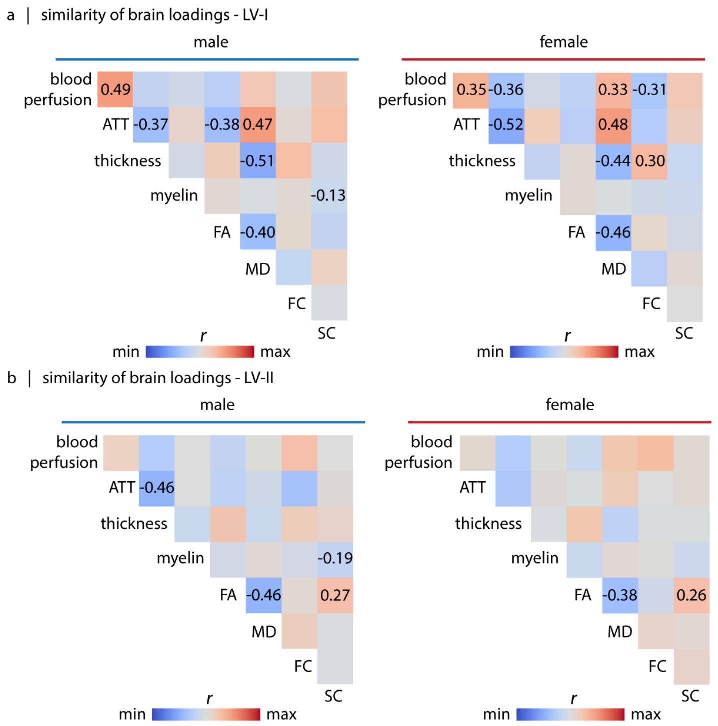

"**Figure S7. Similarity of brain loadings for each latent variable |** (a) The heatmaps show similarity of first latent variable brain loadings across brain measurements. Associations that remain significant after controlling for spatial autocorrelation (using variogram-estimating null models) and multiple comparison correction—using false discovery rate (FDR)—are marked with their corresponding correlation values (ATT and MD: $p_{SMASH} = 0.014$ (males), $p_{SMASH} = 6.99 \times 10^{-3}$ (females); FA and MD: $p_{SMASH} = 9.32 \times 10^{-3}$ (males), $p_{SMASH} = 6.99 \times 10^{-3}$ (females); MD and thickness: $p_{SMASH} = 9.32 \times 10^{-3}$ (males), $p_{SMASH} = 6.99 \times 10^{-3}$ (females); ATT and thickness: $p_{SMASH} = 0.04$ (males), $p_{SMASH} = 6.99 \times 10^{-3}$ (females); perfusion and ATT: $p_{SMASH} = 9.32 \times 10^{-3}$ (males), $p_{SMASH} = 3.15 \times 10^{-2}$ (females), ATT and FA: $p_{SMASH} = 0.04$ (males); myelin and SC: $p_{SMASH} = 0.04$ (males); perfusion and thickness: $p_{SMASH} = 1.68 \times 10^{-2}$ (females); perfusion and FC: $p_{SMASH} = 0.02$ (females); perfusion and MD: $p_{SMASH} = 1.86 \times 10^{-2}$ (females); thickness and FC: $p_{SMASH} = 4.66 \times 10^{-2}$ (females)). (b) The heatmaps show similarity of second latent variable brain loadings across brain measurements (FA and MD: $p_{SMASH} = 6.99 \times 10^{-3}$ (males), $p_{SMASH} = 0.014$ (females); FA and SC: $p_{SMASH} = 6.99 \times 10^{-3}$ (males), $p_{SMASH} = 0.014$ (females); myelin and SC: $p_{SMASH} = 6.99 \times 10^{-3}$ (males); ATT and thickness: $p_{SMASH} = 6.99 \times 10^{-3}$ (males))."

In addition, in Figure S12 (showing LV-I brain loadings after age regression), we have updated the corresponding supplementary figure and revised its caption as follows:

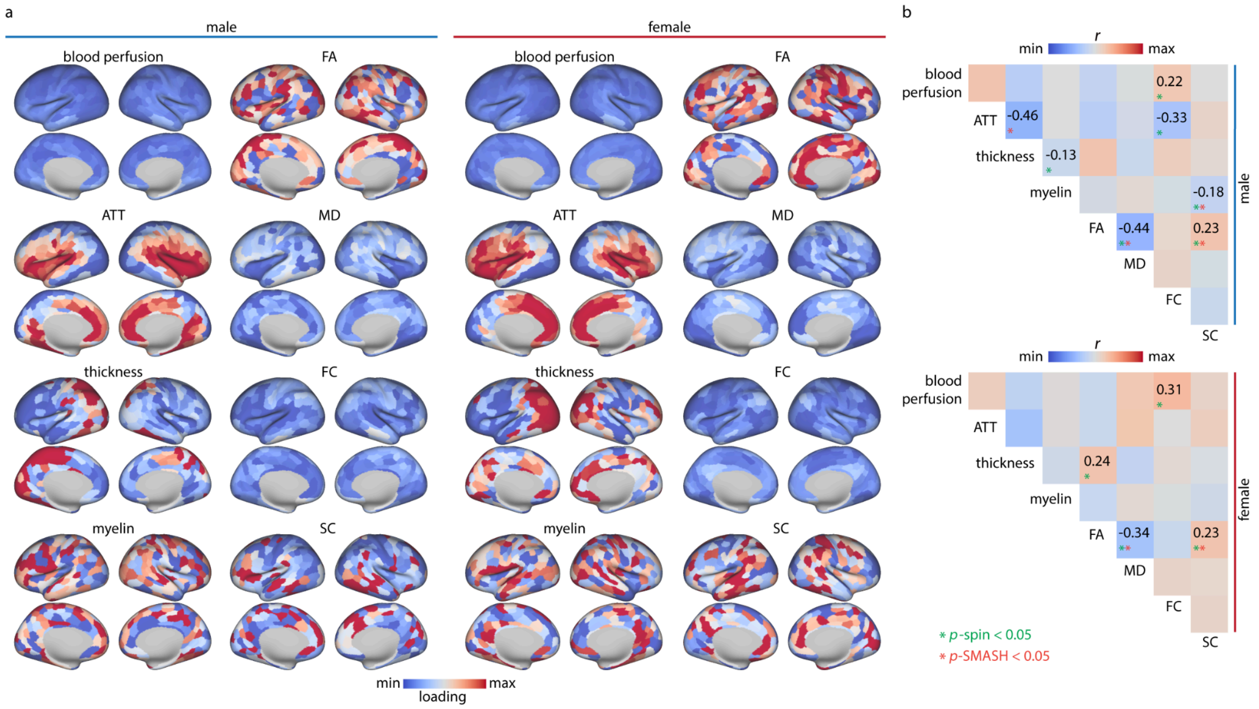

"**Figure S12. LV-I' brain loadings - no age effect |** (a) Brain loadings for each included brain feature. This indicates that participants who more strongly express the biomarkers' pattern illustrated in Fig. 5a (e.g., those with greater BMI) have lower blood perfusion. Brain maps are shown on the fsLR inflated cortical surfaces. (b) Similarity of brain loadings across included brain features. Associations that remain significant after controlling for spatial autocorrelation and false discovery rate (FDR) correction are marked with their corresponding correlation values. Green asterisks indicate significant associations where the significance of empirical correlation is assessed using spin tests ($p_{spin} < 0.05$) and orange asterisks indicate significant associations where the significance of empirical correlation is assessed using variogram-estimating null models ($p_{SMASH} < 0.05$) (FC and blood perfusion: $p_{spin} = 0.042$, $p_{SMASH} > 0.05$ (males), $p_{spin} = 1.86 \times 10^{-2}$, $p_{SMASH} > 0.05$ (females); FA and MD: $p_{spin} = 6.99 \times 10^{-3}$, $p_{SMASH} = 9.32 \times 10^{-3}$ (males), $p_{spin} = 0.014$, $p_{SMASH} = 0.014$ (females); FA and SC: $p_{spin} = 6.99 \times 10^{-3}$, $p_{SMASH} = 9.32 \times 10^{-3}$ (males), $p_{spin} = 0.014$, $p_{SMASH} = 0.014$ (females); myelin and SC: $p_{spin} = 6.99 \times 10^{-3}$, $p_{SMASH} = 9.32 \times 10^{-3}$ (males); ATT and FC: $p_{spin} = 6.99 \times 10^{-3}$, $p_{SMASH} > 0.05$ (males); thickness and myelin: $p_{spin} = 0.042$, $p_{SMASH} > 0.05$ (males); thickness and FA: $p_{spin} = 0.042$, $p_{SMASH} > 0.05$ (females); ATT and thickness: $p_{spin} > 0.5$, $p_{SMASH} = 0.021$ (males)."

Moreover, in Figure S8, S11, and S13, where we compare the similarity of brain loadings across biological sexes, we now report BrainSMASH p-values alongside spin-test p-values. For example, the caption of Figure S8 (shown below) now explicitly distinguishes significance assessed using spin tests (green asterisks) from significance assessed using variogram-estimating null models (orange asterisks). The same modification has been applied consistently across all relevant supplementary figures.

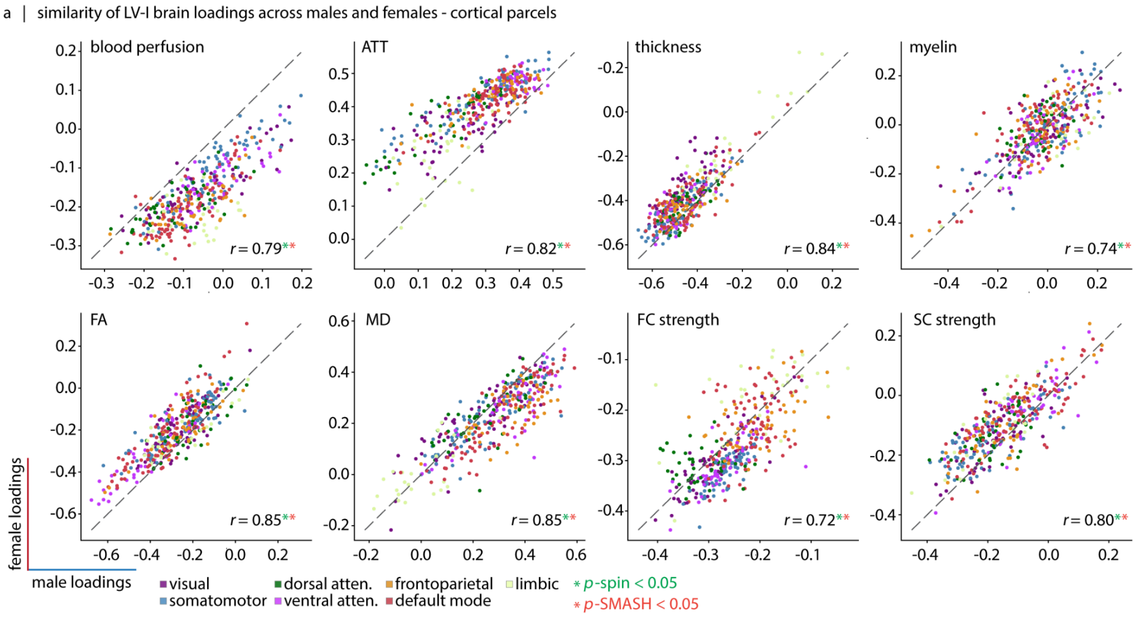

"Figure S8. **Similarity of first latent variable brain loadings between male and female |** (a) Cortical brain loadings comparison. In the scatter plots, each dot represents a brain region defined by the Schaefer-400 parcellation [citation]; the dots are color-coded based on the Yeo-7 functional resting-state networks [citation]. The x-axis shows male brain loadings and the y-axis shows female brain loadings. Green asterisks indicate significant associations where the significance of empirical correlation is assessed using spin tests ($p_{spin} < 0.05$) and orange asterisks indicate significant associations where the significance of empirical correlation is assessed using variogram-estimating null models ($p_{SMASH} < 0.05$). (b)..."

Correspondingly, in the *Results* section, whenever cortical spatial correspondence statistics are reported, we now provide both p_{spin} and p_{SMASH}:

"Given that the most extreme stable biomarker loadings are similar across males and females, we further assess the similarity of brain loadings across sexes. We find the highest correspondence for ATT ($r = 0.82$, FDR-corrected

$p_{perm} = 1.59 \times 10^{-3}$, $p_{SMASH} = 1.33 \times 10^{-3}$) and blood perfusion maps ($r = 0.63$, FDR-corrected $p_{perm} = 1.59 \times 10^{-3}$, $p_{SMASH} = 1.33 \times 10^{-3}$), respectively. Structural connectome strength ($r = 0.06$, FDR-corrected $p_{perm} > 0.05$, $p_{SMASH} > 0.05$), FA ($r = 0.20$, FDR-corrected $p_{perm} = 2.66 \times 10^{-3}$, $p_{SMASH} = 2.28 \times 10^{-3}$), and myelin maps ($r = 0.26$, FDR-corrected $p_{perm} = 1.59 \times 10^{-3}$, $p_{SMASH} = 1.33 \times 10^{-3}$) exhibit lower cross-sex spatial correspondence (Fig. S13)."

We have also added the description of the added null model to the *Methods* section ("*Statistics and null models*" subsection). Both spin tests and BrainSMASH are implemented using the neuromaps toolbox:

> "To assess the effect of spatial autocorrelation on spatial associations between two cortical brain maps, we used two null models, the so-called spatial autocorrelation preserving permutation tests, commonly referred to as "spin tests" [citation] and variogram-estimating null models, referred to as the BrainSMASH method. To implement spin tests, brain phenotypes were projected to spherical projection of the fsaverage surface. This involved selecting the coordinates of the vertex closest to the center of mass for each parcel. These parcel coordinates were then randomly rotated, and original parcels were reassigned to the value of the closest rotated parcel (N repetitions; throughout the manuscript $N = 1,000$). For parcels where the medial wall was the closest, we assigned the value of the next closest parcel instead. Following these steps, we obtained a series of randomized brain maps that have the same values and spatial autocorrelation as the original map but where the relationship between values and their spatial location had been permuted. These maps were then used to generate null distributions of desired statistics. To implement variogram-estimating null model, first the values in a given brain map were randomly permuted, then the permuted values were smoothed and rescaled to reintroduce spatial autocorrelation characteristic of the original, non-permuted map (throughout the manuscript $N = 1,000$) (Burt *et al.*, 2020). Reintroduction of spatial autocorrelation onto the permuted data is achieved via the following transformation $y = |\beta|^{1/2}x' + |\alpha|^{1/2}z$, where x' is the permuted data, $z \sim N(0,1)$ is Gaussian noise, and α and β are estimated via a least-squares optimization to match the variogram of the original data and the permuted data. The resulting maps were then used to generate null distributions of desired statistics. Both spin tests and BrainSMASH were implemented using the neuromaps toolbox (available at *https://github.com/netneurolab/neuromaps/*) [citation]."

Together, these additions demonstrate that our conclusions are relatively robust to the choice of spatial null model.

The following reference is also added to the manuscript:

> “Burt, J. B., Helmer, M., Shinn, M., Anticevic, A., and Murray, J. D. (2020). Generative modeling of brain maps with spatial autocorrelation. NeuroImage, 220:117038.”

Reviewer #3, Fanhua Guo (reviewer 3 has signed this review)

This study uses PLS to identify aging- and metabolism-related axes linking multimodal neuroimaging with peripheral biomarkers in HCP-A and UK Biobank. While the results are compelling, I have several major methodological concerns regarding feature dimensionality imbalance, spatial autocorrelation, and model robustness that warrant substantial revision.

Major:
- The author attempted to expand the number of features in the X matrix in several ways, but this expansion was uneven. For example, the number of features for perfusion, WMH, and myelin differed significantly. Could this introduce a systematic bias into the results? While it might not change the overall trend, could it exaggerate the influence of features like perfusion?

To show that the number of parcels/variables per brain feature is not biasing the PLS loadings, we repeated the PLS analysis using an averaged value per brain feature per participant. Rebuilding the PLS models with this reduced feature set produces latent variables that are highly consistent with those from the original parcel-wise analysis. In both datasets (HCP-A and UK Biobank) and both sexes, LV-I continues to capture age-related brain–body covariation and LV-II continues to capture metabolic dysfunction, confirming that our findings are not an artifact of high dimensionality of the model input. This analysis has been added to the Results section under the "Validation and Sensitivity Analysis" subsection, and the full methodological details and results are also described in the response to Reviewer #2 (see pages 24 to 32).

- Due to the strong spatial autocorrelation between brain regions (e.g., the cortical thickness of adjacent brain regions is extremely similar), does this mean that among the 3000 features in X, there may only be a few dozen effective independent pieces of information? Furthermore, this spatial autocorrelation might be detected by the model and confound some of the results?

Indeed, neuroimaging data is characterized by levels of spatial autocorrelation across adjacent brain regions, rendering them not independent. Importantly, the covariance captured by PLS is computed across individuals—not across brain regions—so spatial smoothness across neighboring parcels does not, by itself, inflate the estimated brain–biomarker relationships. Indeed, PLS has been widely applied in neuroimaging precisely because it accommodates correlated predictors more robustly than classical regression approaches, where multicollinearity can bias parameter estimates [1-3].

Nevertheless, to further address this concern, we repeated the analysis using spatially collapsed brain measures (i.e., a single value per brain feature per individual) and observed highly similar latent variables and score relationships (please see pages 25 to 28). This consistency indicates that the identified brain-biomarker associations are not driven by fine-grained spatial structure.

References:

[1] Wold, S., Ruhe, A., Wold, H., & Dunn, III, W. J. (1984). The collinearity problem in linear regression. The partial least squares (PLS) approach to generalized inverses. *SIAM Journal on Scientific and Statistical Computing*, *5*(3), 735-743.

[2] McIntosh, A. R., Bookstein, F. L., Haxby, J. V., & Grady, E. C. (1996). Spatial pattern analysis of functional brain images using partial least squares. *Neuroimage*, *3*(3), 143-157.

[3] McIntosh, A. R., & Lobaugh, N. J. (2004). Partial least squares analysis of neuroimaging data: applications and advances. *Neuroimage*, *23*, S250-S263.

- The huge dimensional imbalance between brain features (X > 3000) and peripheral biomarkers (Y ~ 20) is a significant concern. While the authors employed standard validation techniques (100 train-test splits and permutation tests) to quantify the overfitting risk, these post-hoc measures do not fundamentally address the inherent problem of too many model parameters in standard PLS under this high-dimensional setting. Standard PLS tends to assign non-zero weights to all features, which may introduce substantial spatial noise and autocorrelation artifacts. Why not use regularization methods, such as sparse PLS? sPLS performs feature selection through L1 penalty during the model fitting process, which not only mitigates the risk of overfitting by design but also yields more concise and biologically meaningful brain-body associations. I suggest the authors compare their current results with those obtained using a sparse modeling framework to demonstrate that the "axes" they identified are not driven by the cumulative effect of low signal-to-noise ratio features.

In response to concerns regarding the stability of the latent variables, we note that several validation analyses were already included in the original manuscript, and we have conducted additional tests in this revision to further show the stability of the results.

In the first version of the manuscript, we showed that similar latent variables were recovered across biological sexes and replicated across two independent datasets (HCP-A and UK Biobank). Within each dataset, we also assessed the stability of covariance patterns found across brain and biomarker using cross-validation: the PLS model and latent variable space were estimated using 80% of the data and then validated on the remaining 20% held-out sample. Cross-validation was done in both biological sex groups and in both datasets.

In the revised manuscript, we performed three additional analyses to further evaluate the robustness and stability of the results:

- We reduced the dimensionality of the brain features by computing global measures for each brain imaging feature (e.g., overall cortical thickness instead of parcel-wise thickness across 400 regions). Despite this substantial reduction in spatial detail, we

recovered highly similar latent variables, which again showed strong within-dataset cross-validation and consistency across sexes.

- We assessed generalizability across cohorts by first fitting the model in the HCP-A dataset and then showing that the model generalizes to the UK Biobank dataset.
- We applied sparse PLS and recovered the same dominant latent structure, indicating that the observed associations are not driven by weak or redundant features (details are followed).

The details of the mentioned second and third analysis are added to the manuscript. Specifically, a subsection called "*Validation and sensitivity analysis*" has been added to the *Results* section of the manuscript and is also included in the response letter (see pages 24 to 32).

As suggested by the reviewer, we replicated the main results of the paper by applying sparse PLS (for HCP-A). Specifically, we implemented the sparse PLS model as described by Monteiro *et al*. Sparse PLS model introduces sparsity on the brain-side weight vector, such that only a subset of brain features receives non-zero weights. In practice, this "feature removal" occurs via soft-thresholding during model fitting, which shrinks small weights to zero and retains only features that contribute most strongly to the multivariate brain–biomarker coupling under the chosen sparsity constraint. We extracted two latent variables separately in males and females.

Across both sexes, LV-I captured an age-related axis of the brain–biomarker covariation. The set of non-zero brain weights for LV-I was dominated by features linked to cortical thickness, arterial arrival time, and diffusion measures (MD and FA). Correlation between brain and biomarker scores for males is equal to $r = 0.75$ ($p_{perm} = 9.99 \times 10^{-4}$) and for females is equal to $r = 0.71$ ($p_{perm} = 9.99 \times 10^{-4}$). The figure below summarizes LV-I and shows biomarker loadings and brain loadings.

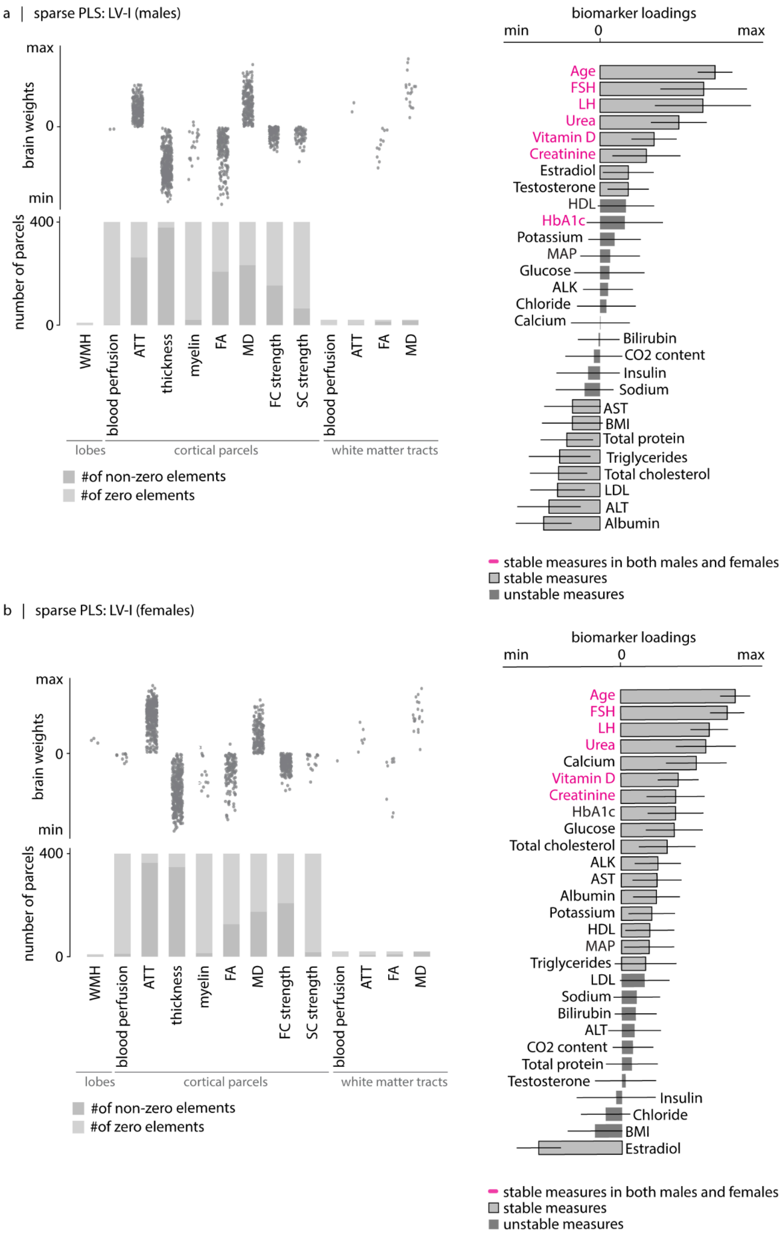

Across both sexes, LV-II captured a metabolic-related axis of the brain–biomarker covariation. The set of non-zero brain weights for LV-II was dominated by features linked to blood perfusion, functional connectivity (FC), and again MD. Correlation between brain and biomarker scores for males is equal to $r = 0.47$ $(p_{perm} = 5.99 \times 10^{-3})$ and for females is equal to $r = 0.46$ $(p_{perm} = 1.19 \times 10^{-2})$. The figure below summarizes LV-II and shows biomarker loadings and brain loadings

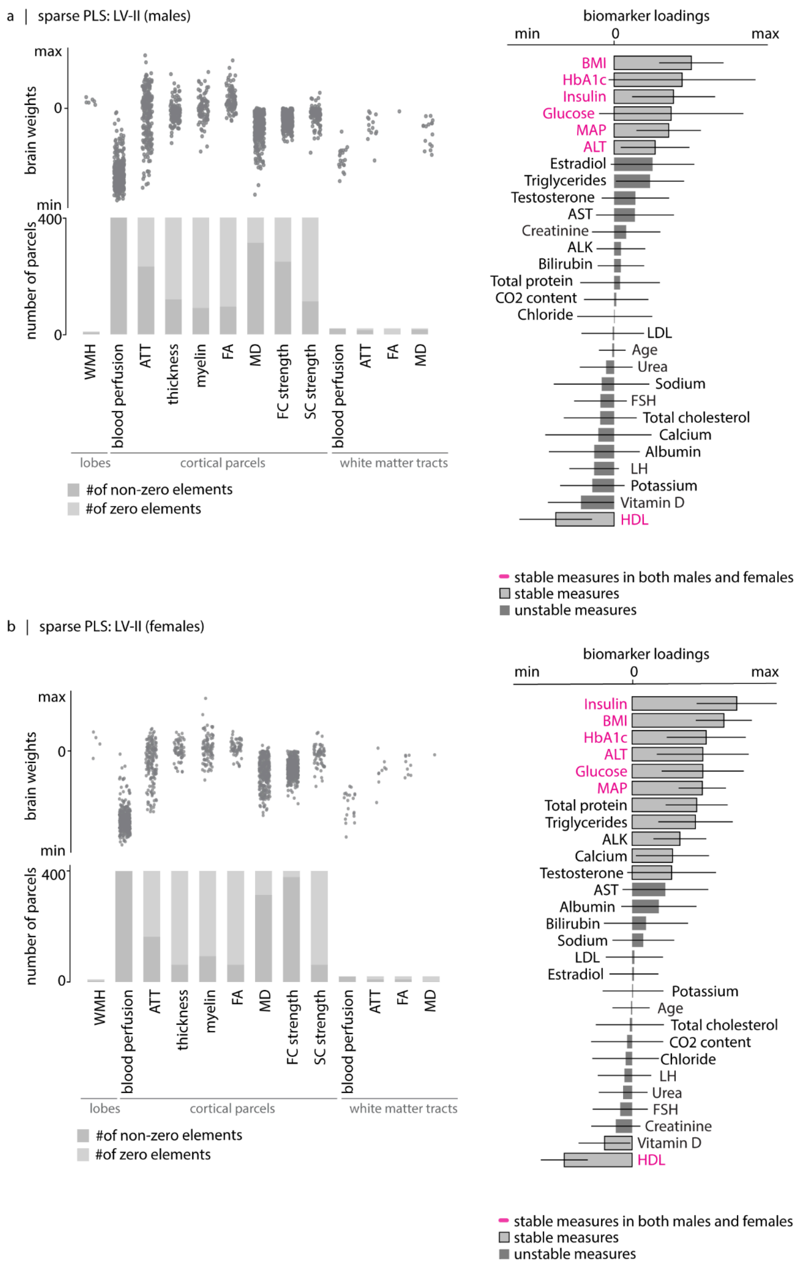

These results confirm that the principal brain–biomarker covariation structure is not driven simply by the large dimensionality of the brain feature space. Even when sparsity is explicitly enforced—so that the model retains only a subset of brain features.

Reference:

Monteiro, J. M., Rao, A., Shawe-Taylor, J., Mourao-Miranda, J., & Alzheimer's Disease Initiative. (2016). A multiple hold-out framework for Sparse Partial Least Squares. *Journal of neuroscience methods*, *271*, 182-194.

- A biological organism is an inherently complex system, and all biomarkers in Y may influence each other in some way. Currently, human capabilities are insufficient to quantify these potential interactions. The problem then arises that the set of these biomarkers will inevitably have some functional bias during analysis. This may introduce a functional bias that could affect the stability of the identified brain-body associations. Could you try randomly removing some features (e.g., 2-10) from Y each time, repeating some times (e.g., 1000), and then ensuring that the relationship between all body biomarkers and the brain imaging score is stable and reliable?

Indeed, biomarkers influence one another through shared physiological and metabolic pathways. We agree with this point and explicitly acknowledge the interdependence among biomarkers in the manuscript. To illustrate this, we include examples of biologically linked peripheral measures. For instance, in the *Results* section we describe a set of known physiological interactions:

> "Excessive weight (high BMI) increases activity of sympathetic nervous system and renin–angiotensin–aldosterone system, leading to elevated blood pressure [citations]. High BMI is also linked to insulin resistance—a biological phenomenon where cells become less responsive to insulin for glucose transport [citation]. Insulin resistance can be indexed by the homeostatic model assessment of insulin resistance (HOMA-IR), derived from fasting insulin and glucose concentration levels (their product divided by a constant) [citation]. At the molecular level, insulin resistance develops through the accumulation of intracellular lipid metabolites, namely diacylglycerol and ceramide, which activate lipid-dependent serine/threonine kinase (including protein kinase C) and impair insulin signaling pathways—through phosphorylation of insulin receptor substrates. Insulin resistance causes impaired insulin-mediated inhibition of lipolysis, leading to increased adipose tissue breakdown and elevated circulating free fatty acids (FFAs) that further inhibit the antilipolytic effect of insulin. Lipotoxic stress and elevated FFAs affect liver function and result in nonalcoholic fatty liver disease, elevated ALT, and systemic inflammation [citations]. Hyperinsulinemia leads to chronic hyperglycemia and increases type 2 diabetes mellitus risk through progressive pancreatic β-cell exhaustion [citation]. Moreover, insulin resistance contributes to endothelial dysfunction and hypertension by impairing the PI3K/Akt pathway responsible for nitric oxide-mediated vasodilation while preserving the MAPK pathway that promotes vasoconstriction through endothelin-1 production. Elevated FFAs further contribute to vasoconstriction through direct effects on vascular smooth muscle cells [citations]. Hyperinsulinemia downregulates muscle lipoprotein lipase, reducing HDL generation from very-low-density lipoprotein processing and impairs insulin's normal suppression of hepatic lipase, leading to increased breakdown of existing HDL particles and their rapid clearance from circulation [citation]."

We have now added two supplementary figures that characterize the similarity among biomarkers across individuals of the same sex. These analyses quantify the extent to which different peripheral measures covary together across subjects and further emphasize the interdependence among biomarkers.

For HCP-A, we have:

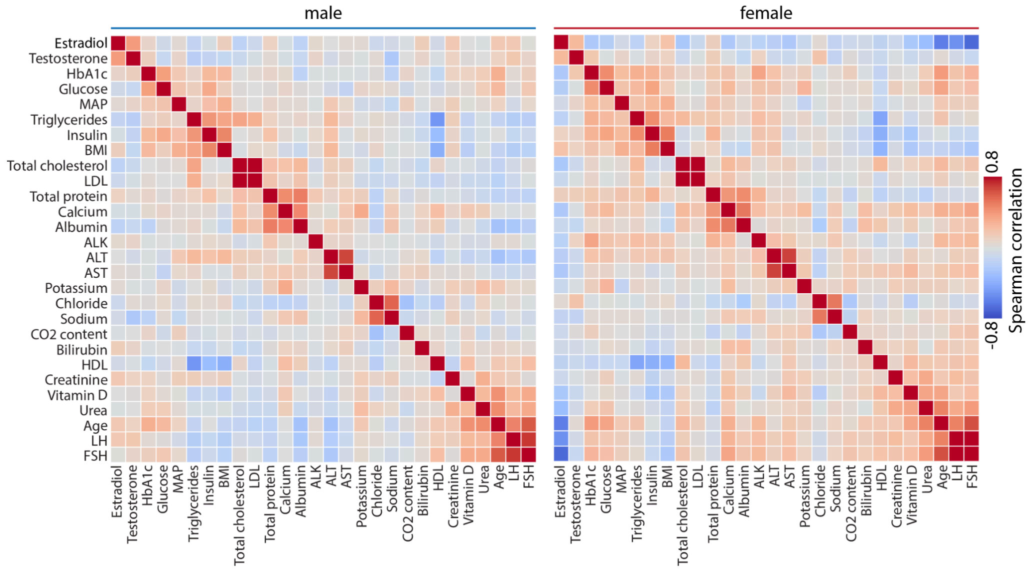

“**Figure S3. Similarity of biomarkers across individuals in the HCP-A dataset | **For each biological sex, we computed pairwise Spearman correlations between biomarkers across individuals.”

And for UK Biobank, we have:

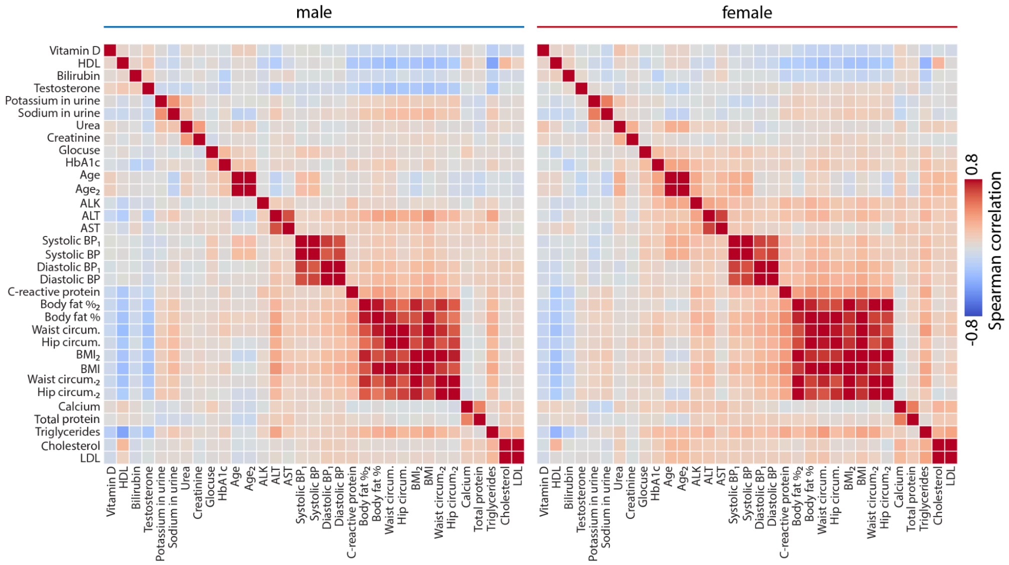

“**Figure S4. Similarity of biomarkers across individuals in the UK Biobank dataset | **For each biological sex, we computed pairwise Spearman correlations between biomarkers across individuals.”

We have added a statement in the *Results* section of the manuscript to refer to these added supplementary figures:

> "We show biomarker intercorrelations for the HCP-A and UK Biobank cohorts in Fig. S3 and S4, respectively."

With regard to the analysis proposed by the reviewer, we agree that in the context of regression models such an approach can be informative, as it can help separate the individual contributions of different biomarkers in shaping a brain outcome. However, in the context of the PLS analysis, adding or removing biomarkers changes the definition of the problem itself and consequently alters the latent variables that are identified. PLS identifies latent variables that maximize the covariance between two datasets (X and Y). In this framework, all variables in the Y matrix jointly contribute to defining the latent axis. If biomarkers are removed from Y, the latent axis itself changes, meaning that the resulting PLS model no longer finds the same brain–body association. Therefore, randomly removing subsets of biomarkers (e.g., 2–10 features) and checking whether the brain score relationship stays stable would not test the stability of the same latent variable. Instead it would create a completely different PLS model each time. As a result, the derived scores would correspond to different biological axes rather than providing a stability assessment of the originally identified association.

In the original manuscript we assessed variable contribution using bootstrap resampling. Following the Reviewer's suggestion, we now also perform dominance analysis (Azen *et al.*) to quantify the relative contribution of individual biomarkers to the prediction of brain scores (for both LV-I and LV-II). As dominance analysis is computationally demanding, we limited the predictors to biomarkers that showed stable contributions to the latent variables of interest (LV-I and LV-II). As shown below, for LV-I (aging axis), we see the dominant contribution of variables including age, urea, FSH, LH, vitamin D, and estradiol in LV-I brain score prediction. When predicting the brain scores for LV-II (metabolic axis), we see the contribution of variables naming BMI, insulin, MAP, HbA1c, glucose, and HDL.

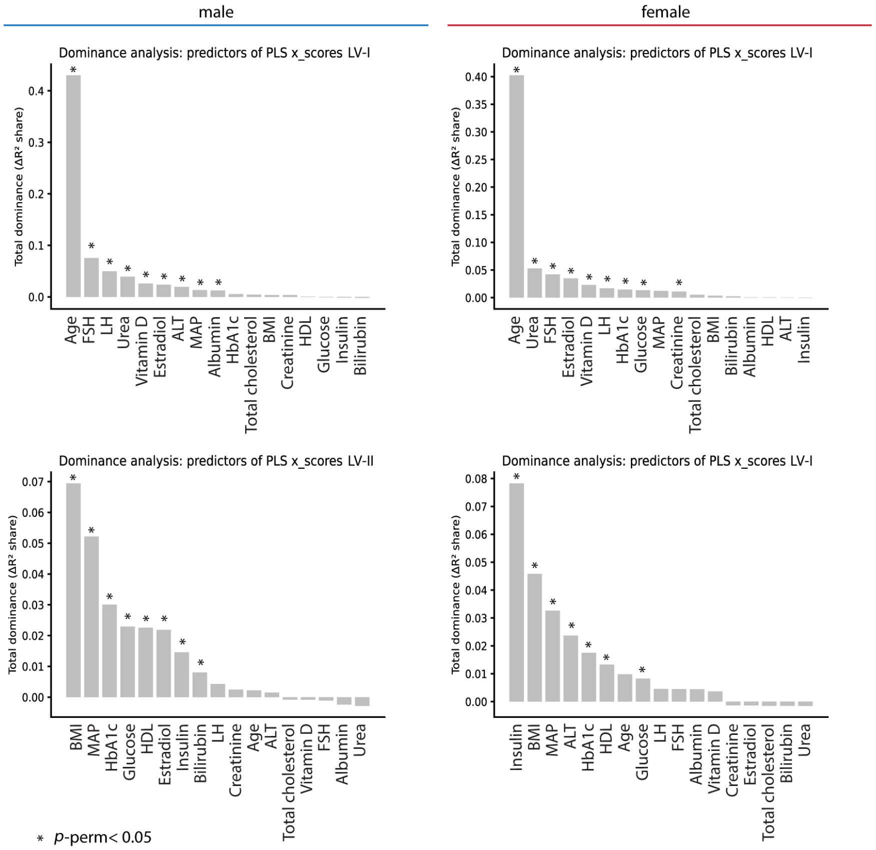

This analysis confirms that when predicting brain patterns associated with LV-I and LV-II, sets of individual biomarkers contribute to the prediction analysis.

Reference:

Azen, R., & Budescu, D. V. (2003). The dominance analysis approach for comparing predictors in multiple regression. *Psychological methods*, *8*(2), 129.

- I noticed that in the UKB data, age also seems to be a significant influencing factor for LV-II. Is it reasonable to use UKB data to validate the conclusion that the second axis is a metabolic axis independent of age? I also noticed that the UKB data analysis considered squared terms for many factors. This is great, and I think it's superior to the approach used in the HCP data. Why didn't the HCP data analysis do the same? If it's due to sample size limitations, could you simulate how large a sample size is needed before including squared terms becomes stable?

To directly address whether the second axis in UKB reflects a metabolic pattern independent of age, we explicitly regressed out age effects from both brain and biomarker features prior to PLS modeling. In this age-regressed analysis, the metabolic pattern is the first latent variable, and age is no longer a significant contributor to the brain–biomarker covariance captured by this latent variable. This analysis is now explained in the manuscript text (*Results* section) and is presented in supplementary materials. In the manuscript we state:

"We further show that the LV-II (metabolic-axis) from UK Biobank is not age-driven. We use GAMLSS models to regress out the nonlinear age effects from all biomarkers and brain features; we then rebuild the PLS model. Fig. S19 shows the first latent variable, which again resembles the biomarker profile associated with metabolic dysfunction and highlights its relevance to blood perfusion...."

The results are shown in the following supplementary figure (Figure S19):

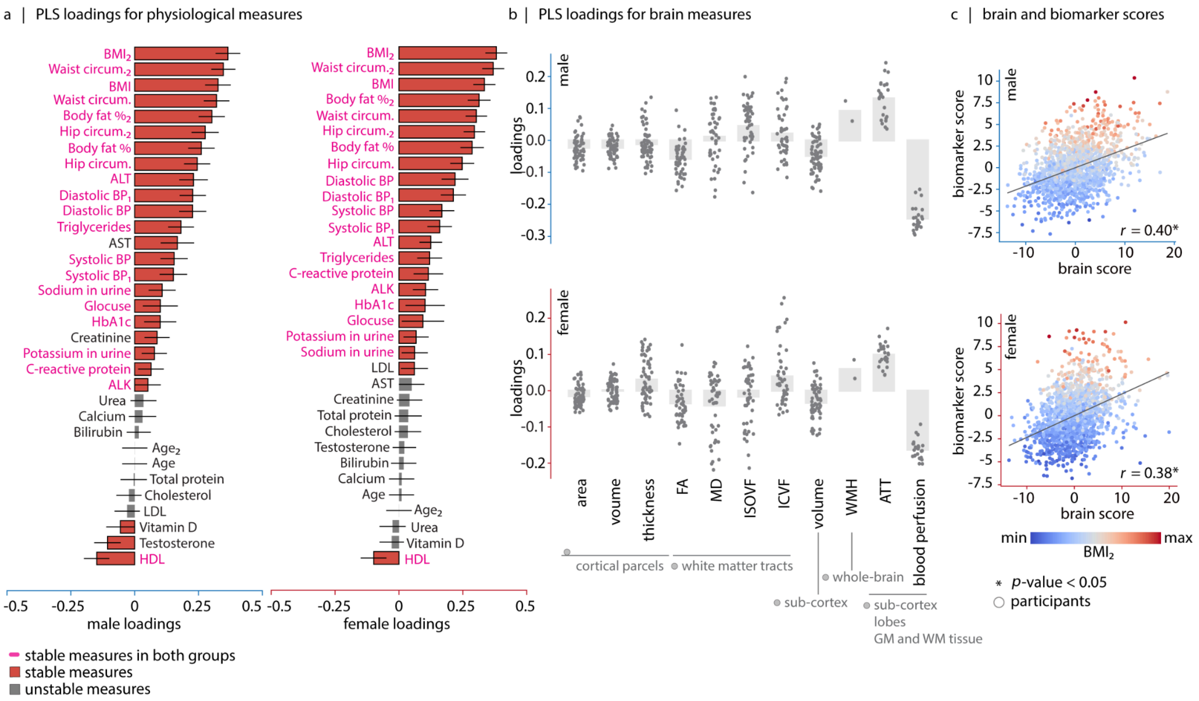

"**Figure S19. Mapping biomarkers to brain features after regressing out the age effect in the UK Biobank dataset: first latent variable (LV-I') captures the metabolic axis |** Using PLS analysis, we identify a significant latent variable that accounts for 65.31% (males) and 60.37% (females) of the covariance in the data. The PLS model includes 33 features on the biomarker side and 490 features on the brain side. (a) Biomarker loadings. Bootstrap resampling is used to estimate the stability of each individual biomarker's contribution to the overall multivariate pattern. Each biomarker loading is divided by its bootstrap-estimated standard error, yielding a measure called "bootstrap ratio". Bootstrap ratio is high for biomarkers with large weights and small standard errors. Stable biomarkers for which the estimated 95\% confidence intervals do not cross zero, are shown in red. BMI, body fat percentage, age, hip and waist circumference were collected at both initial assessment and imaging visits. Variables measured in the imaging visit are denoted with a subscript 2 (e.g., BMI_2), while baseline values are written without subscripts. Subscript 1 is used to indicate repeated

measurements (e.g., $systolic\ blood\ pressure_1$). (b) Brain loadings. Each dot represents a brain region (cortical, subcortical, or a white matter tract). (c) Correlation between brain (x-axis) and biomarker scores (y-axis) for males (top; $r = 0.40$) and females (bottom; $r = 0.38$). Each dot represents an individual participant, colored by their BMI at imaging visit. The score per participant shows the extent to which the participant is expressing the brain-biomarker association captured by LV-I'. Score correlation values passed cross-validation in both sex groups."

We also clarify a potential source of misinterpretation: as stated in the *Methods*, the index "2" in the UK Biobank figures refers to the second data acquisition time point, not to the squared term. In the initial version of the manuscript, the index "2" was formatted as a superscript, which may have led to confusion, we have now revised the notation and present "2" as a subscript throughout all figures to ensure clarity. This point is also added to the relevant figure captions.

That said, we agree with the reviewer that it would be interesting to include squared terms of the input variables (biomarkers) in the analysis framework. We therefore used a "linear + quadratic" biomarker design and re-estimated the PLS model. For this analysis, we incorporated the HCP-A data. The resulting PLS score patterns were highly similar to those obtained from the original model that included only linear terms.

The reason behind this similarity is that quadratic terms are highly collinear with linear terms and do not necessarily change the main covariance axes. In the figure below, we show the correlation between brain and biomarker scores derived from both PLS models, for both latent variables and for each biological sex group. The scores of the two models are highly correlated with one another.

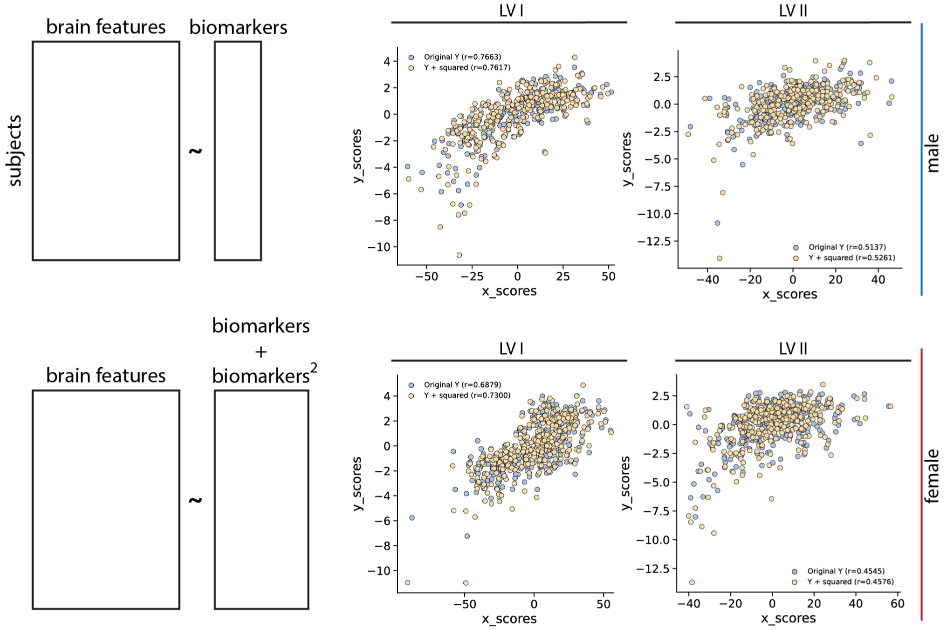

Minor:

- The central premise of using PLS, a fundamentally linear framework, to characterize the interactions within a highly non-linear, complex biological system warrants a critical discussion on model misspecification. Loss of non-linear dynamics: While the methodology is statistically rigorous, linear models may fail to capture essential biological phenomena such as threshold effects, feedback loops, or emergent properties that are characteristic of brain-body health. Questioning mechanistic validity: Does the 'metabolic axis' identified by the model truly represent a distinct biological pathway, or is it a linearized approximation that potentially conflates independent non-linear processes? I suggest the authors explicitly address the limitations of linear assumptions in this context. I won't use this comment as the basis for my review, this is a common problem we face in the early stages of big data mining.

We have added this limitation to the *Discussion* section of the manuscript. We now state that:

> "The present statistical model is sensitive only to linear relationships between brain and body features [1], and does not consider the possibility that multiple such relationships are non-linear. In other words, the latent axes identified in this study do not characterize the full picture of the underlying brain–body associations which may have complex nonlinear nature. As comprehensively phenotyped datasets become larger, it may be possible to fit more complete and complex models that additionally encode non-linear relationships between brain features and systemic biomarkers."

Reference:

[1] McIntosh, A. R., & Lobaugh, N. J. (2004). Partial least squares analysis of neuroimaging data: applications and advances. *Neuroimage*, *23*, S250-S263.

- The decision to perform sex-stratified analysis a priori rather than including biological sex as a feature within the Y matrix is worth further justification. By stratifying the groups, the study presupposes that the entire brain-body covariance structure is fundamentally different between sexes, rather than allowing the model to empirically 'discover' the relative importance of sex compared to other metabolic and demographic factors. Given the paper's emphasis on a 'data-driven' approach, this theory-driven partition feels somewhat unsmooth.

Our decision to perform sex-stratified analyses was motivated by two main reasons listed below:

(1) There exist well-documented and systematic biological differences between males and females in both brain measures and systemic biomarkers. For example, females typically exhibit higher global cerebral blood perfusion than males, and sex differences are also widely reported in circulating biomarkers such as creatinine and lipid profiles, as well as

in hormones. The rationale for stratifying the analyses by biological sex is stated in the *Results* section:

"Throughout this report, we stratify the analysis by biological sex. There exist known differences between males and females in both brain and biomarker measures. For example, females typically exhibit higher cerebral blood perfusion than males [citations], along with distinct hormonal trajectories and notable differences in certain plasma markers (e.g., creatinine, and lipid markers) [citations]."

(2) This choice aligns with emerging best practices in neuroimaging and biomedical research, which increasingly emphasize examining biological sex explicitly rather than treating it solely as a covariate to be adjusted away. A substantial body of evidence demonstrates that sex differences operate across multiple biological scales, from gene expression and cellular signaling to organ-level physiology and disease vulnerability. Interestingly, metabolic and vascular processes have been shown to have meaningful sex differences in physiology and risk profiles. Accordingly, our decision to stratify analyses by biological sex in this manuscript is consistent with the growing recognition that future neuroimaging and biomedical research should move toward sex-stratified designs. Such designs enable us to better capture mechanistic understanding of potential sex differences and can ultimately advance sex-stratified medicine, including improved understanding of women's health [1-7].

References:

[1] Clayton, J. A., & Collins, F. S. (2014). Policy: NIH to balance sex in cell and animal studies. *Nature*, *509*(7500), 282-283.

[2] Clayton, J. A. (2015). Studying both sexes: a guiding principle for biomedicine. *The FASEB Journal*, *30*(2), 519.

[3] Regitz-Zagrosek, V., & Kararigas, G. (2017). Mechanistic pathways of sex differences in cardiovascular disease. *Physiological reviews*, *97*(1), 1-37.

[4] Mauvais-Jarvis, F., Clegg, D. J., & Hevener, A. L. (2013). The role of estrogens in control of energy balance and glucose homeostasis. *Endocrine reviews*, *34*(3), 309.

[5] Shafee, R., Moraczewski, D., Liu, S., Mallard, T., Thomas, A., & Raznahan, A. (2024). A sex-stratified analysis of the genetic architecture of human brain anatomy. *Nature Communications*, *15*(1), 8041.

[6] Yang, M. L., Xu, C., Gupte, T., Hoffmann, T. J., Iribarren, C., Zhou, X., & Ganesh, S. K. (2024). Sex-specific genetic architecture of blood pressure. *Nature medicine*, *30*(3), 818-828.

[7] Rosenzweig, N., Kleemann, K. L., Rust, T., Carpenter, M., Grucci, M., Aronchik, M., ... & Butovsky, O. (2024). Sex-dependent APOE4 neutrophil–microglia interactions drive cognitive impairment in Alzheimer's disease. *Nature Medicine*, *30*(10), 2990-3003.

[8] Westergaard, D., Moseley, P., Sørup, F. K. H., Baldi, P., & Brunak, S. (2019). Population-wide analysis of differences in disease progression patterns in men and women. *Nature communications*, *10*(1), 666.
