# Supplementary material for: Aging and metabolism contribute separately to brain–body health
Source: PLoS Biol. 2026 Jun 15;24(6):e3003856. doi: 10.1371/journal.pbio.3003856 (PMC13293518; doi:10.1371/journal.pbio.3003856)

Dear Dr. Schnell, Dr. Smith, and Reviewers,

We thank the editors and the reviewer for the constructive engagement during the revision process, and for the opportunity to improve the manuscript. Following your suggestions, we have revised the manuscript. In this letter, we respond to each of the reviewers' comments in detail. Reviewer comments are in **bold,** and our responses are in regular font.

Reviewer #1 (Frauke Beyer signed her report):
The authors addressed all of my comments and provided additional informative analyses.
I have one remaining question:
Could you provide the correlation of individual loadings in the UKB derived from the UKB-based PLS and the HCP-A-based PLS. This would give an additional confirmation that really the same constructs are measured. Until now you have shown that the HCP-A based PLS also generates correlated scores in the UKB, but not that these are the same necessarily.

We did not include a direct comparison of brain loadings between the HCP-A and UK Biobank in the original manuscript for two reasons:

First, the parcellation schemes differ substantially between the two datasets. UK Biobank provides preprocessed imaging-derived phenotypes (IDPs) at relatively coarse spatial resolution. For example, white matter hyperintensities are summarized into two measures, and arterial spin labeling-derived metrics, blood perfusion and arterial transit time, are reported as single values per lobe (e.g., one value for the occipital lobe). This coarse spatial resolution is partly a result of large imaging voxel sizes. In contrast, HCP-A offers higher imaging quality and spatial resolution, enabling a more detailed parcellation. These discrepancies limit the direct comparability of loadings across datasets.

Second, the age distributions across the two cohorts are not matched. Age is a key factor influencing metabolic and brain-related measures, and its effects may vary across different life stages. HCP-A spans 36-100 years and the UK Biobank spans 51-83 years. Though we expect the directionality of associations to be consistent across datasets, for instance, cortical thickness decreasing with age or worse metabolic profiles in both datasets, we do not necessarily expect the spatial patterns to overlap completely. How these latent variables evolve across age ranges is an important question that needs further investigation and can help identify age windows in which metabolic changes exert the greatest influence on brain structure.

That said, for modalities where harmonization was feasible, we performed additional correspondence analyses. Specifically, for cortical thickness, we mapped Desikan–Killiany atlas-based loadings to the Schaefer-400 parcellation and observed positive spatial correspondence between datasets for both latent variables and across sexes. Similarly, for white matter measures (FA and MD), we restricted analyses to tracts with consistent naming across datasets and again assessed the correspondence of brain loadings across datasets.

We observe that thickness and MD loadings are positively correlated across cohorts. However, for FA, specifically for the second latent variable, we do not see significant positive associations. This might arise from the fact that MD of white matter tracts is a relatively weak contributor to this latent variable (see Figure 3 and sFigure 17).

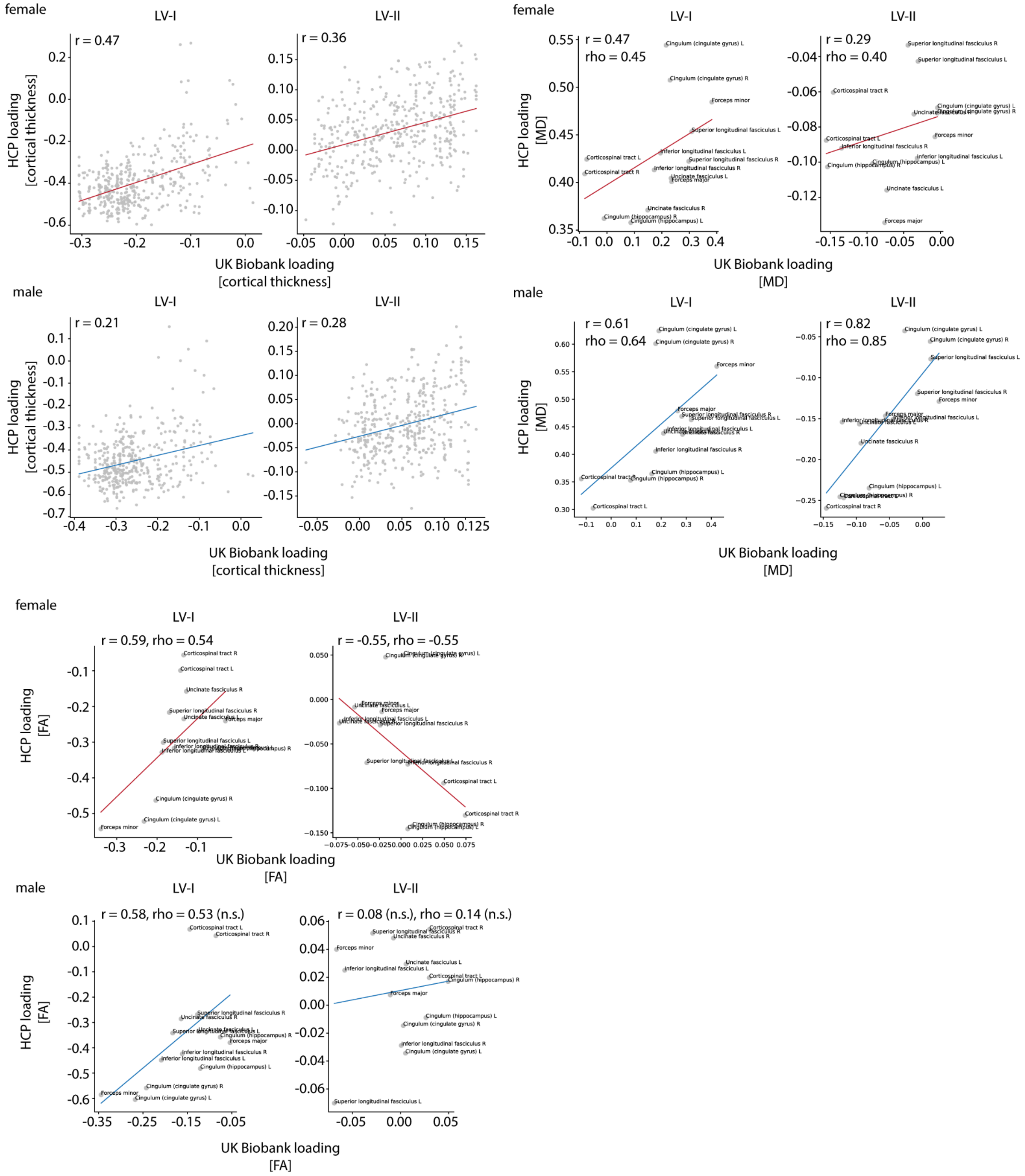

Reviewer #2:
This is a substantially improved revision. My remaining concerns are slightly over strong language. For example, the age-independent metabolic axis appears to replicate, but the out-of-sample effect size in UKB is modest. I would temper the strong language about generalizability. I also think the "resilient/vulnerable phenotype" framing remains stronger than evidence for cross-sectional data.

We have adjusted the tone in the *Results* section accordingly. The revised text is provided below.

Specifically, we change:

> ”We next ask whether a PLS model trained on one dataset (HCP-A) generalizes to explain brain–biomarker relationships in an independent cohort (UK Biobank).”

to:

> “We next ask whether a PLS model trained on one dataset (HCP–A) explains brain–biomarker relationships in an independent cohort (UK Biobank).”

Furthermore, we change:

> ”Together, these results demonstrate that the brain–biomarker covariance structure identified in HCP-A—including both the age-dominated and metabolic latent variables—generalizes to an independent dataset.”

to:

> ”Together, these results demonstrate that the brain–biomarker covariance structure identified in HCP–A—including both the age–dominated and metabolic latent variables—shows correspondence in an independent dataset. Notably, when the model developed on the HCP–A data is used to explain the associations in the UK Biobank data, the resulting score correlation values are modest relative to the discovery sample, which may reflect differences in age range and imaging granularity across datasets.”

We agree with the reviewer that the cross-sectional nature of the study precludes direct assessment of whether an individual is resilient or vulnerable to metabolic burden over their lifespan. Indeed, longitudinal imaging and biomarker acquisition are needed to track whether relative brain preservation or susceptibility persists across repeated measurements. We have now expanded this point in the *Discussion* as follows:

> “This study provides a framework for studying the mechanisms of neuroprotection and vulnerability in metabolic dysfunction. Not all individuals exposed to metabolic dysfunction experience equivalent brain outcomes. Some individuals demonstrate resilience to expected metabolic brain correlates despite

peripheral risk factors, while some show heightened vulnerability (discrepancy between brain and peripheral biomarker profiles; see Fig. S27). While part of this discrepancy may originate from measurement errors, it is also conceivable that genetic predisposition and environmental factors modulate the heterogeneity of interactions between brain and peripheral features (Assis et al., 2025). Our framework quantifies this discrepancy and provides an opportunity for targeted genetic investigations to identify genetic variants that may confer resilience to brain alterations caused by metabolic risks and to test the contribution of behavioral and lifestyle factors—such as smoking, physical activity, socioeconomic status, and pollution exposure—on shaping this discrepancy. One plausible example of how genetics can alter the association between brain and bodily metabolic signature is detailed here: vascular dysfunction and arterial stiffness partially mediate the link between systemic metabolic conditions and brain blood flow, function and structure (Pasha et al., 2018), meanwhile, specific genes are known to contribute to individuals' propensity to develop arterial stiffening (Lacolley et al., 2009; Cecelja et al., 2012), which may, in return, amplify the downstream effects of metabolic abnormalities on the brains of these vulnerable individuals. It is important to note that in the present report resilience and vulnerability are defined with respect to normative brain-body associations estimated across the full cohort. These cross-sectional findings should therefore be replicated in longitudinal studies to assess individual vulnerability and resilience over time."

Reviewer #3 (Fanhua Guo signed his report):
The author has addressed most of my concerns. Once the last few minor issues are resolved, I believe the work will be ready for publication.
Comments:
- The additional analysis using spatially averaged brain features (Fig. S22) is helpful and alleviates concerns regarding extreme high dimensionality. However, this raises a more fundamental issue: if a reduced and more balanced representation of the brain features yields similar results, it is unclear why the primary conclusions continue to rely on the original high-dimensional, parcel-wise model, which is more susceptible to biases related to feature redundancy and spatial autocorrelation. In other words, the consistency between models demonstrates robustness of the findings, but does not justify the continued use of a potentially less well-posed model as the primary analytical framework.

Including the high-dimensional, parcel-wise analysis in the main figures serves two purposes that the spatially averaged model cannot fulfill. First, it confirms that the observed loading patterns are not driven by a small number of regions with extreme values (outliers). Second, this

spatially resolved approach enables the comparison of loading patterns across various brain measures (Figures 2 and 4).

To ensure that the high dimensionality of the model input data does not lead to model instability, we also replicated the PLS analysis using spatially averaged brain maps (reduced input dimensionality). We observe highly consistent latent variables across both datasets and both biological sexes, regardless of whether the model is fit using the full high-dimensional or the reduced feature set. This convergence confirms that the identified brain–body associations are not artifacts of input high dimensionality.

In the manuscript, we have acknowledged the potential limitation of high-dimensional PLS models and have cited the relevant literature discussing this concern (Helmer et al., 2024). We have also provided detailed analyses addressing this issue in the main text of the manuscript (part of which is included below), with corresponding figures included in the supplementary material. Readers now have access to both sets of analyses for reference.

> "We also assess the potential instability of PLS results arising from the high dimensionality of the brain feature space relative to sample size (Helmer et al., 2024). To this end, each brain feature is averaged across all parcels, yielding a single global value per brain measure per participant and substantially reducing the PLS input dimensionality. Rebuilding the PLS models using this reduced feature set yields latent variables that are highly consistent with those obtained from the original analysis. Across both biological sexes and both datasets (HCP–A and UK Biobank), LV–I captures brain–body covariation related to aging and LV–II captures covariation related to metabolic dysfunction, confirming that the main findings are not artifacts of the high dimensionality of the PLS model input."

- I strongly agree with a point raised by Reviewer 2: the primary metrics within the data are inherently derived from metabolic processes. The metabolic latent variable aggregates a diverse set of physiological measures, including lipid markers, glucose metabolism, adiposity, and liver enzymes, which reflect distinct biological processes. While these variables are correlated, it remains unclear whether the identified axis represents a unified metabolic mechanism or a statistical amalgamation of heterogeneous processes. The manuscript would benefit from a more fine-grained analysis to disentangle whether specific metabolic domains differentially relate to brain measures, particularly cerebral perfusion.

We agree with the reviewer that the composition of the biomarker set is biased toward measures that reflect metabolic processes; this bias can contribute to the prominence of the metabolic latent variable reported in this study. At the same time, we note that these measures (e.g., lipid profiles, BMI, glucose-related markers) are routinely assessed in clinical practice due to their established relevance to general health. In the manuscript, we have acknowledged that expanding the biomarker space can uncover additional dimensions of brain–body interaction. As

datasets with both brain imaging and peripheral bodily measures become increasingly available, this represents an important direction for future work. We have now added this point to the *Discussion* in the first set of revisions:

> "Last, many of the biomarkers included in this study are directly linked to the metabolic status of the body and are highly interconnected (e.g., HDL, LDL, BMI, total cholesterol). Indeed, the choice of biomarkers shapes the reported axes of brain–body covariance in this report and contributes to the prominence of the metabolic latent variable. Future work incorporating more diverse and comprehensive plasma and urine measures may reveal latent brain–body dimensions that extend beyond the metabolic axis."

Many of the included physiological measures are intercorrelated. However, fully disentangling their individual mechanistic contributions is not within the scope of the present study; yet, it is an avenue for future investigations. We are not convinced that the diverse set of physiological measures (lipid markers, glucose metabolism, adiposity, and liver enzymes) can be considered fully "distinct" biological processes, as these features all affect and are affected by one another.

To ensure that the latent variables we report here are not derived by an amalgamation of related physiological measures, we have added a new univariate analysis to the response letter, showing the association between cerebral perfusion and selected physiological measures in the HCP-A cohort, separately in males and females. Specifically, we examined BMI and HDL as representative metabolic markers, and Urea as a negative control. These analyses show that even when the field of view is narrowed from the full multivariate pattern to individual markers, cerebral perfusion remains associated with key metabolic measures such as BMI and HDL, whereas this relationship is not observed for Urea. This pattern supports our interpretation that the identified multivariate latent variable is not merely an arbitrary amalgamation of correlated physiological measures, but is meaningfully anchored in individual metabolic markers that show clear correspondence with brain perfusion (see figure below).

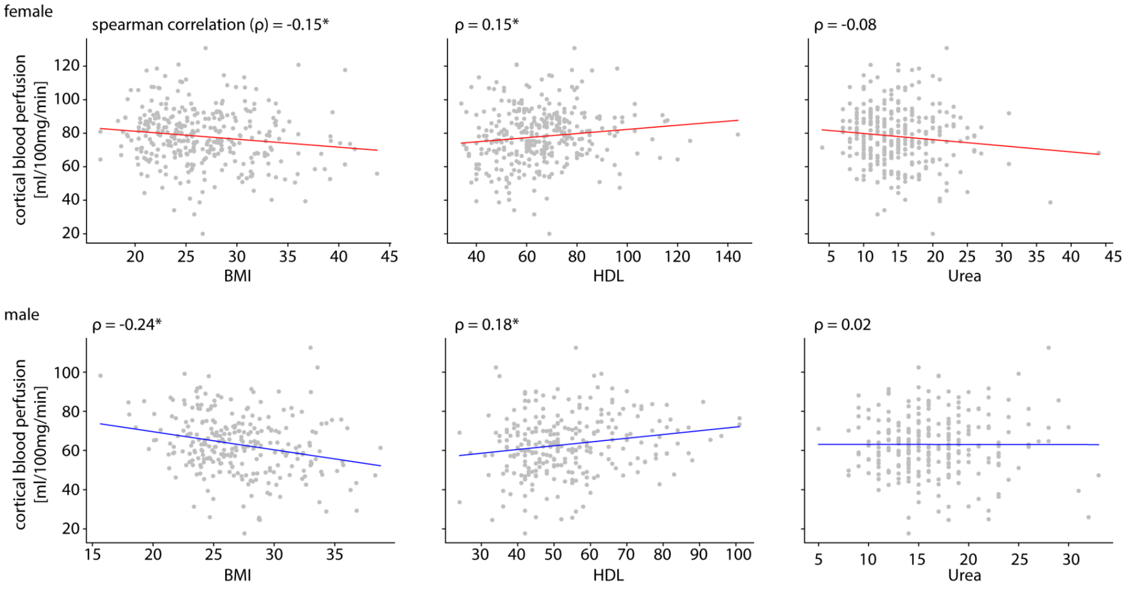

Supplement: Attachment — Submitted filename: Response_letter_auresp_3.pdf [file pbio.3003856.s031.pdf]
