# Supplementary material for: Aging and metabolism contribute separately to brain–body health
Source: PLoS Biol. 2026 Jun 15;24(6):e3003856. doi: 10.1371/journal.pbio.3003856 (PMC13293518; doi:10.1371/journal.pbio.3003856)
Supplement: Peer review history

# Author comment: Author response to Decision Letter 0

**DOI:** 10.1371/journal.pbio.3003856.r001

**Transfer Alert**

This paper was transferred from another journal. As a result, its full editorial history (including decision letters, peer reviews and author responses) may not be present.

20 Nov 2025

# Editor report: Decision Letter 0

**DOI:** 10.1371/journal.pbio.3003856.r002

**Authors:** Lucas Smith

2 Dec 2025

Dear Bratislav,

Thank you for submitting your manuscript entitled "Separable contributions of aging and metabolism to brain-body health" for consideration as a Research Article by PLOS Biology and please accept my apologies for the delay in sending you an initial decision. The assessment process for your study took a bit longer than normal because of the Thanksgiving break last week.

Your manuscript has now been evaluated by the PLOS Biology editorial staff and I am writing to let you know that we would like to send your submission out for external peer review.

However, before we can send your manuscript to reviewers, we need you to complete your submission by providing the metadata that is required for full assessment. To this end, please login to Editorial Manager where you will find the paper in the 'Submissions Needing Revisions' folder on your homepage. Please click 'Revise Submission' from the Action Links and complete all additional questions in the submission questionnaire.

Once your full submission is complete, your paper will undergo a series of checks in preparation for peer review. After your manuscript has passed the checks it will be sent out for review. To provide the metadata for your submission, please Login to Editorial Manager (https://www.editorialmanager.com/pbiology) within two working days, i.e. by Dec 04 2025 11:59PM.

If your manuscript has been previously peer-reviewed at another journal, PLOS Biology is willing to work with those reviews in order to avoid re-starting the process. Submission of the previous reviews is entirely optional and our ability to use them effectively will depend on the willingness of the previous journal to confirm the content of the reports and share the reviewer identities. Please note that we reserve the right to invite additional reviewers if we consider that additional/independent reviewers are needed, although we aim to avoid this as far as possible. In our experience, working with previous reviews does save time.

If you would like us to consider previous reviewer reports, please edit your cover letter to let us know and include the name of the journal where the work was previously considered and the manuscript ID it was given. In addition, please upload a response to the reviews as a 'Prior Peer Review' file type, which should include the reports in full and a point-by-point reply detailing how you have or plan to address the reviewers' concerns.

During the process of completing your manuscript submission, you will be invited to opt-in to posting your pre-review manuscript as a bioRxiv preprint. Visit http://journals.plos.org/plosbiology/s/preprints for full details. If you consent to posting your current manuscript as a preprint, please upload a single Preprint PDF.

Feel free to email us at plosbiology@plos.org if you have any queries relating to your submission.

Kind regards,

Luke

Lucas Smith, Ph.D.

Senior Editor

PLOS Biology

lsmith@plos.org

# Peer review: Decision Letter 1

**DOI:** 10.1371/journal.pbio.3003856.r003

**Authors:** Lucas Smith

13 Feb 2026

Dear Bratislav,

Thank you for your patience while your manuscript "Separable contributions of aging and metabolism to brain-body health" was peer-reviewed at PLOS Biology and I apologize again for the protracted review process. Your study has now been evaluated by the PLOS Biology editors, an Academic Editor with relevant expertise, and by several independent reviewers.

In light of the reviews, which you will find at the end of this email, we would like to invite you to revise the work to thoroughly address the reviewers' reports.

As you will see below, while the reviewers note several strengths in the study, reviewers 2 and 3 have identified a number of important concerns, including with the methodology and modeling choices, and some of the interpretations. After discussion with the team and cross-reviewer commenting, we think these issues will need to be thoroughly addressed with additional analyses and clearer framing of the conclusions, before we can consider your study for publication. Addressing the reviews may require you to rethink aspects of your modeling approach, to specifically address the role of age in the PLS model, stability for high dimensionality, the replication versus validation distinction, and some statistical choices such as spatial null models, as suggested by the reviewers. We also think it would also be appropriate to tone down some of the novelty claims.

Given the extent of revision needed, we cannot make a decision about publication until we have seen the revised manuscript and your response to the reviewers' comments. Note, that given the nature and scope of the reviewer concerns, we will necessarily have a high bar for publication - and will only move forward with the study if the reviewers are satisfied that the methodological issues have been adequately addressed.

In addition to these revisions, you will need to complete some formatting changes, which you will receive in a follow up email. A member of our team will be in touch with a set of requests shortly.

We expect to receive your revised manuscript within 3 months, but are happy to provide an extension if needed. Please email us (plosbiology@plos.org) if you have any questions or concerns, or would like to request an extension.

At this stage, your manuscript remains formally under active consideration at our journal; please notify us by email if you do not intend to submit a revision so that we may withdraw it.

**IMPORTANT - SUBMITTING YOUR REVISION**

Your revisions should address the specific points made by each reviewer. Please submit the following files along with your revised manuscript:

1. A 'Response to Reviewers' file - this should detail your responses to the editorial requests, present a point-by-point response to all of the reviewers' comments, and indicate the changes made to the manuscript.

*NOTE: In your point-by-point response to the reviewers, please provide the full context of each review. Do not selectively quote paragraphs or sentences to reply to. The entire set of reviewer comments should be present in full and each specific point should be responded to individually, point by point.

You should also cite any additional relevant literature that has been published since the original submission and mention any additional citations in your response.

2. In addition to a clean copy of the manuscript, please also upload a 'track-changes' version of your manuscript that specifies the edits made. This should be uploaded as a "Revised Article with Changes Highlighted" file type.

*Re-submission Checklist*

When you are ready to resubmit your revised manuscript, please refer to this re-submission checklist: https://plos.io/Biology_Checklist

To submit a revised version of your manuscript, please go to https://www.editorialmanager.com/pbiology/ and log in as an Author. Click the link labelled 'Submissions Needing Revision' where you will find your submission record.

Please make sure to read the following important policies and guidelines while preparing your revision:

*Published Peer Review*

Please note while forming your response, if your article is accepted, you may have the opportunity to make the peer review history publicly available. The record will include editor decision letters (with reviews) and your responses to reviewer comments. If eligible, we will contact you to opt in or out. Please see here for more details:

https://blogs.plos.org/plos/2019/05/plos-journals-now-open-for-published-peer-review/

*PLOS Data Policy*

Please note that as a condition of publication PLOS' data policy (http://journals.plos.org/plosbiology/s/data-availability) requires that you make available all data used to draw the conclusions arrived at in your manuscript. If you have not already done so, you must include any data used in your manuscript either in appropriate repositories, within the body of the manuscript, or as supporting information (N.B. this includes any numerical values that were used to generate graphs, histograms etc.). For an example see here: http://www.plosbiology.org/article/info%3Adoi%2F10.1371%2Fjournal.pbio.1001908#s5

*Blot and Gel Data Policy*

We require the original, uncropped and minimally adjusted images supporting all blot and gel results reported in an article's figures or Supporting Information files. We will require these files before a manuscript can be accepted so please prepare them now, if you have not already uploaded them. Please carefully read our guidelines for how to prepare and upload this data: https://journals.plos.org/plosbiology/s/figures#loc-blot-and-gel-reporting-requirements

*Protocols deposition*

To enhance the reproducibility of your results, we recommend that if applicable you deposit your laboratory protocols in protocols.io, where a protocol can be assigned its own identifier (DOI) such that it can be cited independently in the future. Additionally, PLOS ONE offers an option for publishing peer-reviewed Lab Protocol articles, which describe protocols hosted on protocols.io. Read more information on sharing protocols at https://plos.org/protocols?utm_medium=editorial-email&utm_source=authorletters&utm_campaign=protocols

Thank you again for your submission to our journal. We hope that our editorial process has been constructive thus far, and we welcome your feedback at any time. Please don't hesitate to contact us if you have any questions or comments.

Sincerely,

Luke

Lucas Smith, Ph.D.

Senior Editor

PLOS Biology

lsmith@plos.org

------------------------------------

REVIEWS:

Reviewer #1, Frauke Beyer (note, reviewer 1 has signed this review): In this manuscript, Farahani et al. derive latent components describing the association of anthropometric and metabolic measures and brain imaging phenotypes. The first component pair is dominated by age and age-related metabolic changes relating to decreased structural brain integrity. The second component, independent of age, shows an association of metabolic features like HDL, obesity, mean arterial pressure, HbA1c with primarily cerebral perfusion. The patterns are consistent in men and women and across two population-based cohorts.

The manuscript is well written, the analyses are clear and can be thoroughly followed in the provided code. The results are clearly presented and discussed. I also liked that all analyses are performed sex-stratified.

I only have a few comments which may help to improve this nice manuscript.

Results:

- throughout the manuscript, there sometimes is confusion about the direction of the axis, e.g. in the abstract you write about reduced perfusion, and what is described in this paragraph: - "LV-II biomarker profile is highlight-

ing low HDL and age, along with high BMI, hip and

waist circumferences, body fat percentage, diastolic

blood pressure, ALT, aspartate aminotransferase

(AST), triglyceride, c-reactive protein—previously

linked to metabolic syndrome and inflammatory dis-

orders [227]—and sodium in urine. This metabolic

profile is dominantly associated with lower brain

blood perfusion (Fig. S14b and Fig. S15)"

is shown inversely in Figure S14 (i.e. low BMI, high age..). Maybe all directions could be swapped so that higher scores in the metabolic component are associated with higher metabolic risk ?

- regarding the validation in UKB: maybe it would be feasible to use the HCP-A decomposition (potentially without indicators missing in UKB) and apply it to the UKB data to see agreement instead of performing completely new analysis? I am wondering whether you could even more clearly say that the same profiles are found if you followed this approach.

- It is not clear to me what Figures S6 etc. are supposed to show, could you make it more visible (that highest loadings are for perfusion, I guess). Could you provide a comparison between sexes for the metabolic factors too, to convince readers the components are really similar between sexes?

Discussion:

"Some individuals demonstrate resilience to expected metabolic

brain correlates despite peripheral risk factors, while

some show heightened vulnerability (discrepancy

between brain and peripheral biomarker profiles;

see Fig. 3c, 5c, S14c and S16c)."

→ explain in more detail, and highlight two examples in the figure.

I don't fully understand why you included this (interesting) paragraph

"The present study is part of a wider effort to

understand links between the brain and the body.

These bidirectional interactions are increasingly ex-

plored in the context of the gut-brain axis [142]

and the proteomics-brain axis [93, 155]. The gut-

brain axis consists of two communication mecha-

nisms: one mediated based on chemicals including,

neurotransmitters, neuropeptides, hormones, and

gut microbiome [159], and the other mediated by

the physical connections via the vagus nerve. This

axis is increasingly recognized as important for un-

derstanding the spread of proteinopathies in neu-

rodegenerative diseases [181, 229]. Furthermore,

there is evidence that this axis of interaction plays

a role in obesity and metabolic health via seroton-

ergic signaling [92, 98]. Concurrently, proteomics

can also help to monitor brain health, and specif-

ically to define protein signatures associated with

obesity, type 2 diabetes, and neurodegenerative dis-

eases. The identified protein profiles enable disease

vulnerability predictions and identification of poten-

tial target molecules for effective intervention plans

[93, 119, 155, 176, 203, 236]" - maybe shorten if you want to make a point that other "-brain axis" exist?

- I don't agree with the term "powerful" here: "the present report shows that

a composite metabolic brain-body profile may be a

powerful way to identify links between physiologi-

cal measurements and cognitive outcome." as the associations are fairly weak (especially in the UKBB) and you don' show that the physiological scores derived from PLS are more associated with cognition than e.g. BMI alone.

- in the interpretation of the sex differences, you mention "Our results point to possible sex differences in metabolic-cognitive associations." I miss a statement about the similarity of the especially metabolic component as such (which are surprisingly similar, I find). It does not seem that body fat % or any hormonal measure (e.g. estradiol) has a significantly different weight in males vs females. Is this because differences would be rather expected within gender (i.e. women with higher levels of estradiol are protected against metabolic risk factors)? I would recommend to extend the discussion on this point.

Methods:

How did you select the biomarkers used in the UKBB? (mention somewhere these were all biomarkers you used for HCP-A & available in UKBB if that's accurate)

"We also performed

an additional PLS analysis in which the effect of age

effect was regressed out from all brain imaging and

physiological features." → remove one "effect", which age (initial or imaging assessment) was regressed?

Reviewer #2: The paper by Farahani and co authors, titled " Separable contributions of aging and metabolism to brain-body health" and submitted to the journal PLOS Biology, presents a partial least square analyses to relate various physiological variables to various brain variables.

Age is a difficult variable to include in the analyses. The age regressed partial least square analyses should be used for the main results instead as a sensitive analyses. First, it is the dominant source of variance in the analyses. Second, it is conceptually different from any other measure used in the analyses. The other data on that side of the equation are physiological measurements. Age is not that. It is deterministic, continuously increasing during life, and absolute. You can only get older. But you can change all other measures (whether by lifestyle or drugs). Finally, given how much age impacts all aspect of life (from development to aging), it is likely a cause factor of changes in both brain and body. Finally, when age is removed, the 1st component looks like the 2nd component of the uncorrected model, again suggesting that the first component is just age.

Some of the results are circular in the sense that most of the non brain variables use are metabolic measures. Thus I'm not surprised the largest component is a metabolic factor. Though in numerous places in the text, it is suggested that it is novel that a latent factor of metabolism was found through data driven methods.

Was age regressed from the cognitive variables?

The validation analyses is not "validation". It is replication. The authors repeated their analytical approach in a second dataset. Validation would be more along the lines how using latent factors found in the HPC dataset in the UK biobank dataset.

It would be helpful to state the number of features on each side of the plsr analyses in the main text. It is unclear at the start of the paper how many imaging features are being used.

Along those lines, why was the HCP aging dataset used as the discovery dataset? The dimensions are very high compared to the sample size: 300 subjects (since males and females had separate models) compared to 28 biological features and 3000 brain features. This leads to unstable loading estimates (see helmer et al). It would be more logical to use the larger UKB as the main dataset with the HCP as the replication dataset.

Helmer M, Warrington S, Mohammadi-Nejad AR, Ji JL, Howell A, Rosand B, Anticevic A, Sotiropoulos SN, Murray JD. On the stability of canonical correlation analysis and partial least squares with application to brain-behavior associations. Commun Biol. 2024 Feb 21;7(1):217. doi: 10.1038/s42003-024-05869-4. PMID: 38383808; PMCID: PMC11245620.

The spin test has numerous statistical issues centered around exchangeability violation. Something like Brain Smash has much better statistical properties and should be used.

Burt JB, Helmer M, Shinn M, Anticevic A, Murray JD. Generative modeling of brain maps with spatial autocorrelation. Neuroimage. 2020 Oct 15;220:117038. doi: 10.1016/j.neuroimage.2020.117038. Epub 2020 Jun 22. PMID: 32585343.

Reviewer #3, Fanhua Guo (note, reviewer 3 has signed this review): This study uses PLS to identify aging- and metabolism-related axes linking multimodal neuroimaging with peripheral biomarkers in HCP-A and UK Biobank. While the results are compelling, I have several major methodological concerns regarding feature dimensionality imbalance, spatial autocorrelation, and model robustness that warrant substantial revision.

Major:

--The author attempted to expand the number of features in the X matrix in several ways, but this expansion was uneven. For example, the number of features for perfusion, WMH, and myelin differed significantly. Could this introduce a systematic bias into the results? While it might not change the overall trend, could it exaggerate the influence of features like perfusion?

--Due to the strong spatial autocorrelation between brain regions (e.g., the cortical thickness of adjacent brain regions is extremely similar), does this mean that among the 3000 features in X, there may only be a few dozen effective independent pieces of information? Furthermore, this spatial autocorrelation might be detected by the model and confound some of the results?

--The huge dimensional imbalance between brain features (X > 3000) and peripheral biomarkers (Y ~ 20) is a significant concern. While the authors employed standard validation techniques (100 train-test splits and permutation tests) to quantify the overfitting risk, these post-hoc measures do not fundamentally address the inherent problem of too many model parameters in standard PLS under this high-dimensional setting. Standard PLS tends to assign non-zero weights to all features, which may introduce substantial spatial noise and autocorrelation artifacts. Why not use regularization methods, such as sparse PLS? sPLS performs feature selection through L1 penalty during the model fitting process, which not only mitigates the risk of overfitting by design but also yields more concise and biologically meaningful brain-body associations. I suggest the authors compare their current results with those obtained using a sparse modeling framework to demonstrate that the "axes" they identified are not driven by the cumulative effect of low signal-to-noise ratio features.

--A biological organism is an inherently complex system, and all biomarkers in Y may influence each other in some way. Currently, human capabilities are insufficient to quantify these potential interactions. The problem then arises that the set of these biomarkers will inevitably have some functional bias during analysis. This may introduce a functional bias that could affect the stability of the identified brain-body associations. Could you try randomly removing some features (e.g., 2-10) from Y each time, repeating some times (e.g., 1000), and then ensuring that the relationship between all body biomarkers and the brain imaging score is stable and reliable?

--I noticed that in the UKB data, age also seems to be a significant influencing factor for LV-II. Is it reasonable to use UKB data to validate the conclusion that the second axis is a metabolic axis independent of age? I also noticed that the UKB data analysis considered squared terms for many factors. This is great, and I think it's superior to the approach used in the HCP data. Why didn't the HCP data analysis do the same? If it's due to sample size limitations, could you simulate how large a sample size is needed before including squared terms becomes stable?

Minor:

--The central premise of using PLS, a fundamentally linear framework, to characterize the interactions within a highly non-linear, complex biological system warrants a critical discussion on model misspecification. Loss of non-linear dynamics: While the methodology is statistically rigorous, linear models may fail to capture essential biological phenomena such as threshold effects, feedback loops, or emergent properties that are characteristic of brain-body health. Questioning mechanistic validity: Does the 'metabolic axis' identified by the model truly represent a distinct biological pathway, or is it a linearized approximation that potentially conflates independent non-linear processes? I suggest the authors explicitly address the limitations of linear assumptions in this context. I won't use this comment as the basis for my review, this is a common problem we face in the early stages of big data mining.

--The decision to perform sex-stratified analysis a priori rather than including biological sex as a feature within the Y matrix is worth further justification. By stratifying the groups, the study presupposes that the entire brain-body covariance structure is fundamentally different between sexes, rather than allowing the model to empirically 'discover' the relative importance of sex compared to other metabolic and demographic factors. Given the paper's emphasis on a 'data-driven' approach, this theory-driven partition feels somewhat unsmooth.

# Author comment: Author response to Decision Letter 2

**DOI:** 10.1371/journal.pbio.3003856.r004

18 Mar 2026

Attachment Submitted filename: Response_letter.pdf

# Peer review: Decision Letter 2

**DOI:** 10.1371/journal.pbio.3003856.r005

**Authors:** Lucas Smith

22 Apr 2026

Dear Dr Misic,

Thank you for your patience while we considered your revised manuscript "Separable contributions of aging and metabolism to brain-body health" for consideration as a Research Article at PLOS Biology. Your revised study has now been evaluated by the PLOS Biology editors, the Academic Editor and the original reviewers.

In light of the reviews, which you will find at the end of this email, we are pleased to offer you the opportunity to address the remaining points from the reviewers in a revision that we anticipate should not take you very long. We will then assess your revised manuscript and your response to the reviewers' comments with our Academic Editor aiming to avoid further rounds of peer-review, although we might need to consult with the reviewers, depending on the nature of the revisions.

In addition to these revisions, you may need to complete some formatting changes, which you will receive in a follow up email. A member of our team will be in touch with a set of requests shortly. If you do not receive a separate email within a few days, please assume that checks have been completed, and no additional changes are required.

We expect to receive your revised manuscript within 1 month. Please email us (plosbiology@plos.org) if you have any questions or concerns, or would like to request an extension.

At this stage, your manuscript remains formally under active consideration at our journal; please notify us by email if you do not intend to submit a revision so that we withdraw the manuscript.

**IMPORTANT - SUBMITTING YOUR REVISION**

Your revisions should address the specific points made by each reviewer. Please submit the following files along with your revised manuscript:

1. A 'Response to Reviewers' file - this should detail your responses to the editorial requests, present a point-by-point response to all of the reviewers' comments, and indicate the changes made to the manuscript.

*NOTE: In your point-by-point response to the reviewers, please provide the full context of each review. Do not selectively quote paragraphs or sentences to reply to. The entire set of reviewer comments should be present in full and each specific point should be responded to individually.

You should also cite any additional relevant literature that has been published since the original submission and mention any additional citations in your response.

2. In addition to a clean copy of the manuscript, please also upload a 'track-changes' version of your manuscript that specifies the edits made. This should be uploaded as a "Revised Article with Changes Highlighted " file type.

*Resubmission Checklist*

When you are ready to resubmit your revised manuscript, please refer to this resubmission checklist: https://plos.io/Biology_Checklist

To submit a revised version of your manuscript, please go to https://www.editorialmanager.com/pbiology/ and log in as an Author. Click the link labelled 'Submissions Needing Revision' where you will find your submission record.

Please make sure to read the following important policies and guidelines while preparing your revision:

*Published Peer Review*

Please note while forming your response, if your article is accepted, you may have the opportunity to make the peer review history publicly available. The record will include editor decision letters (with reviews) and your responses to reviewer comments. If eligible, we will contact you to opt in or out. Please see here for more details:

https://blogs.plos.org/plos/2019/05/plos-journals-now-open-for-published-peer-review/

*PLOS Data Policy*

Please note that as a condition of publication PLOS' data policy (http://journals.plos.org/plosbiology/s/data-availability) requires that you make available all data used to draw the conclusions arrived at in your manuscript. If you have not already done so, you must include any data used in your manuscript either in appropriate repositories, within the body of the manuscript, or as supporting information (N.B. this includes any numerical values that were used to generate graphs, histograms etc.). For an example see here: http://www.plosbiology.org/article/info%3Adoi%2F10.1371%2Fjournal.pbio.1001908#s5

*Blot and Gel Data Policy*

We require the original, uncropped and minimally adjusted images supporting all blot and gel results reported in an article's figures or Supporting Information files. We will require these files before a manuscript can be accepted so please prepare them now, if you have not already uploaded them. Please carefully read our guidelines for how to prepare and upload this data: https://journals.plos.org/plosbiology/s/figures#loc-blot-and-gel-reporting-requirements

*Protocols deposition*

To enhance the reproducibility of your results, we recommend that if applicable you deposit your laboratory protocols in protocols.io, where a protocol can be assigned its own identifier (DOI) such that it can be cited independently in the future. Additionally, PLOS ONE offers an option for publishing peer-reviewed Lab Protocol articles, which describe protocols hosted on protocols.io. Read more information on sharing protocols at https://plos.org/protocols?utm_medium=editorial-email&utm_source=authorletters&utm_campaign=protocols

Thank you again for your submission to our journal. We hope that our editorial process has been constructive thus far, and we welcome your feedback at any time. Please don't hesitate to contact us if you have any questions or comments.

Sincerely,

Christian

Christian Schnell, Ph.D.

Senior Editor

PLOS Biology

cschnell@plos.org

on behalf of

Lucas Smith, Ph.D.

Senior Editor

PLOS Biology

lsmith@plos.org

----------------------------------------------------------------

REVIEWS:

Reviewer #1 (Frauke Beyer signed her report): The authors adressed all of my comments and provided additional informative analyses.

I have one remaining question:

Could you provide the correlation of individual loadings in the UKB derived from the UKB-based PLS and the HCP-A-based PLS. This would give an additional confirmation that really the same constructs are measured. Until now you have shown that the HCP-A based PLS also generates correlated scores in the UKB, but not that these are the same necessarily.

Reviewer #2: This is a substantially improved revision. My remaining concerns are slightly over strong language. For example, the age-independent metabolic axis appears to replicate, but the out-of-sample effect size in UKB is modest. I would temper the strong language about generalizability. I also think the "resilient/vulnerable phenotype" framing remains stronger than evidence for cross-sectional data.

Reviewer #3 (Fanhua Guo signed his report): The author has addressed most of my concerns. Once the last few minor issues are resolved, I believe the work will be ready for publication.

Comments:

- The additional analysis using spatially averaged brain features (Fig. S22) is helpful and alleviates concerns regarding extreme high dimensionality. However, this raises a more fundamental issue: if a reduced and more balanced representation of the brain features yields similar results, it is unclear why the primary conclusions continue to rely on the original high-dimensional, parcel-wise model, which is more susceptible to biases related to feature redundancy and spatial autocorrelation. In other words, the consistency between models demonstrates robustness of the findings, but does not justify the continued use of a potentially less well-posed model as the primary analytical framework.

- I strongly agree with a point raised by Reviewer 2: the primary metrics within the data are inherently derived from metabolic processes. The metabolic latent variable aggregates a diverse set of physiological measures, including lipid markers, glucose metabolism, adiposity, and liver enzymes, which reflect distinct biological processes. While these variables are correlated, it remains unclear whether the identified axis represents a unified metabolic mechanism or a statistical amalgamation of heterogeneous processes. The manuscript would benefit from a more fine-grained analysis to disentangle whether specific metabolic domains differentially relate to brain measures, particularly cerebral perfusion.

# Author comment: Author response to Decision Letter 3

**DOI:** 10.1371/journal.pbio.3003856.r006

27 Apr 2026

Attachment Submitted filename: Response_letter_auresp_3.pdf

# Peer review: Decision Letter 3

**DOI:** 10.1371/journal.pbio.3003856.r007

**Authors:** Lucas Smith

21 May 2026

Dear Bratislav,

Thank you for your patience while we considered your revised manuscript "Separable contributions of aging and metabolism to brain-body health" for publication as a Research Article at PLOS Biology. This revised version of your manuscript has been evaluated by the PLOS Biology editors, the Academic Editor and two of the original reviewers.

As you will see in their comments, below, both reviewers are now fully satisfied with your revision. Based on their feedback, we are likely to accept this manuscript for publication. However before we can do so, we need you to address a few last data and other policy-related requests, in a revision that we anticipate will not take very long. These requests are detailed below.

----

**IMPORTANT - please address the following editorial requests:

1) TITLE: We would like to propose a tweak to the title, to make it more declarative. If you agree, we suggest you change it to something like:

"Aging and metabolism contribute separately to brain-body health"

2) FINANCIAL DISCLOSURES STATEMENT: Please update your financial disclosures statement in our online system, to include the funder websites.

3) ETHICS STATMENTS: Thank you for providing ethics statements for the data from the HCP and UK Biobank.

a. for the HCP - if possible, please update this to include the protocol number(s). Please also indicate whether participants gave informed written consent to participate in the study.

b. for the UK Biobank - please indicate whether the data was collected in accordance with the principles expressed in the Declaration of Helsinki. Please add the protocol number(s) as well, if possible.

c. please also update the methods to include the ethics committee, and approval number that approved the research conducted in this current manuscript, and indicate whether your study was conducted in accordance with the principles expressed in the Declarations of Helsinki. (My understanding is that, to use HCP data that you need approval from your own Institutional Review Board or Ethics Committee - but if you are exempt from this, please indicate which IRB/Ethics committee has deemed this research exempt).

4) DATA AND CODE STATEMENT:

a. Thank you for providing a link to your code deposition on GitHub. Please note that we cannot accept sole deposition of code in GitHub, as this could be changed after publication. However, you can archive this version of your publicly available GitHub code to Zenodo. Once you do this, it will generate a DOI number, which you will need to provide in the Data Accessibility Statement (you are welcome to also provide the GitHub access information). See the process for doing this here: https://docs.github.com/en/repositories/archiving-a-github-repository/referencing-and-citing-content

b. please update your Data and code availability statement, in our online system, to explain why you have not shared the raw underlying data for your study (ie. that the sharing of these data are restricted by the UK Biobank and HCP), and to provide more details about how researchers might gain access to it. I

Ex. you could say something like: Due to the UKB/HCP's data sharing policy, the data used in this study are available upon application to the UKB/HCP (https://www.ukbiobank.ac.uk/use-our-data/apply-for-access/ ___HCP website__).

^ (please edit for accuracy, etc)

c. While we understand that you are not able to legally share the raw data underlying your study, if possible, we ask that you provide the processed, numerical data underlying the graphs presented in your study (including supplemental figures). These data can be provided as an excel file called 'S1_data', or as a deposition to a public repository - and this file should be referenced in every relevant figure legend.

Regardless of the method selected, please ensure that you provide the individual numerical values that underlie the summary data displayed in your figures. NOTE: the numerical data provided should include all replicates AND the way in which the plotted mean and errors were derived (it should not present only the mean/average values).

Please ensure that your Data Statement in the submission system accurately describes where your data can be found.

----

As you address these items, please take this last chance to review your reference list to ensure that it is complete and correct. If you have cited papers that have been retracted, please include the rationale for doing so in the manuscript text, or remove these references and replace them with relevant current references. Any changes to the reference list should be mentioned in the cover letter that accompanies your revised manuscript.

In addition to these revisions, you may need to complete some formatting changes, which you will receive in a follow up email. A member of our team will be in touch with a set of requests shortly. If you do not receive a separate email within a few days, please assume that checks have been completed, and no additional changes are required.

We expect to receive your revised manuscript within two weeks.

To submit your revision, please go to https://www.editorialmanager.com/pbiology/ and log in as an Author. Click the link labelled 'Submissions Needing Revision' to find your submission record. Your revised submission must include the following:

- a cover letter that should detail your responses to any editorial requests, if applicable, and whether changes have been made to the reference list

- a Response to Reviewers file that provides a detailed response to the reviewers' comments (if applicable, if not applicable please do not delete your existing 'Response to Reviewers' file.)

- a track-changes file indicating any changes that you have made to the manuscript.

NOTE: If Supporting Information files are included with your article, note that these are not copyedited and will be published as they are submitted. Please ensure that these files are legible and of high quality (at least 300 dpi) in an easily accessible file format. For this reason, please be aware that any references listed in an SI file will not be indexed. For more information, see our Supporting Information guidelines:

https://journals.plos.org/plosbiology/s/supporting-information

*Published Peer Review History*

Please note that you may have the opportunity to make the peer review history publicly available. The record will include editor decision letters (with reviews) and your responses to reviewer comments. If eligible, we will contact you to opt in or out. Please see here for more details:

https://plos.org/published-peer-review-history/

*Press*

Should you, your institution's press office or the journal office choose to press release your paper, please ensure you have opted out of Early Article Posting on the submission form. We ask that you notify us as soon as possible if you or your institution is planning to press release the article.

*Protocols deposition*

To enhance the reproducibility of your results, we recommend that if applicable you deposit your laboratory protocols in protocols.io, where a protocol can be assigned its own identifier (DOI) such that it can be cited independently in the future. Additionally, PLOS ONE offers an option for publishing peer-reviewed Lab Protocol articles, which describe protocols hosted on protocols.io. Read more information on sharing protocols at https://plos.org/protocols?utm_medium=editorial-email&utm_source=authorletters&utm_campaign=protocols

Please do not hesitate to contact me should you have any questions.

Sincerely,

Luke

Lucas Smith, Ph.D.

Senior Editor

lsmith@plos.org

PLOS Biology

------------------------------------------------------------------------

Reviewer remarks:

Reviewer #1, Frauke Beyer (note, reviewer 1 has signed this review): I am satisfied with the answer and recommend publication of the manuscript.

Reviewer #3, Fanhua Guo (note, reviewer 1 has signed this review): The author addressed my concerns, and I recommend accepting it.

# Author comment: Author response to Decision Letter 4

**DOI:** 10.1371/journal.pbio.3003856.r008

27 May 2026

Attachment Submitted filename: Response_letter_auresp_4.pdf

# Editor report: Decision Letter 4

**DOI:** 10.1371/journal.pbio.3003856.r009

**Authors:** Lucas Smith

1 Jun 2026

Dear Bratislav,

Thank you for the submission of your revised Research Article "Aging and metabolism contribute separately to brain-body health" for publication in PLOS Biology and thank you for addressing our last editorial requests in this revision. On behalf of my colleagues and the Academic Editor, Claus C. Hilgetag, I am pleased to say that we can in principle accept your manuscript for publication, provided you address any remaining formatting and reporting issues. These will be detailed in an email you should receive within 2-3 business days from our colleagues in the journal operations team; no action is required from you until then. Please note that we will not be able to formally accept your manuscript and schedule it for publication until you have completed any requested changes.

**IMPORTANT - as you address any production requests, to come, please also address the following editorial requests.

1 - Thank you for providing me with an updated financial disclosures statement with the funder URLs over email. Please be sure to update the statement in our online editorial manager system to include these details.

2 - Thank you for providing me with your data access request ID numbers for your approved use of the HCP and UKBB datasets. Please do update the ethics statements contained within your methods section to include the details that you supplied over email.

3 - Lastly, thank you for providing me with the link to your Github deposition, containing the processed numerical data for your study (that you are allowed to share). As mentioned over email, please be sure to update your data and code availability statement to include an up-to-date Zenodo DOI.

Please take a minute to log into Editorial Manager at http://www.editorialmanager.com/pbiology/, click the "Update My Information" link at the top of the page, and update your user information to ensure an efficient production process.

PRESS

We frequently collaborate with press offices. If your institution or institutions have a press office, please notify them about your upcoming paper at this point, to enable them to help maximise its impact. If the press office is planning to promote your findings, we would be grateful if they could coordinate with biologypress@plos.org. If you have previously opted in to the early version process, we ask that you notify us immediately of any press plans so that we may opt out on your behalf.

We also ask that you take this opportunity to read our Embargo Policy regarding the discussion, promotion and media coverage of work that is yet to be published by PLOS. As your manuscript is not yet published, it is bound by the conditions of our Embargo Policy. Please be aware that this policy is in place both to ensure that any press coverage of your article is fully substantiated and to provide a direct link between such coverage and the published work. For full details of our Embargo Policy, please visit http://www.plos.org/about/media-inquiries/embargo-policy/.

Thank you again for choosing PLOS Biology for publication and supporting Open Access publishing. We look forward to publishing your study.

Sincerely,

Luke

Lucas Smith, Ph.D.

Senior Editor

PLOS Biology

lsmith@plos.org
